# Discriminative performance of externally validated dementia risk prediction models: a systematic review and meta-analysis

**DOI:** 10.1186/s12916-026-04652-y

**Published:** 2026-02-02

**Authors:** Blossom C. M. Stephan, Jacob Brain, Kaarin J. Anstey, Tanya Buchanan, Claire V. Burley, Elissa Burton, Jennifer Dunne, Linda Errington, Matthew Gorringe, Zhongyang Guan, Bronwyn Myers, Serena Sabatini, Marc Sim, William Stephan, Eugene Yee Hing Tang, Narelle Warren, Mario Siervo

**Affiliations:** 1 Dementia Centre of Excellence, Curtin enAble Institute, Curtin University, Perth, WA Australia; 2 Institute of Mental Health, School of Medicine, University of Nottingham, Jubilee Campus, Nottingham, UK; 3 Freemasons Foundation Centre for Men’s Health, Discipline of Medicine, School of Psychology, The University of Adelaide, Adelaide, SA Australia; 4 School of Psychology, University of New South Wales, Sydney, Australia; 5 UNSW Ageing Futures Institute, Sydney, Australia; 6 Neuroscience Research Australia, Margarete Ainsworth Building, Randwick, NSW 2031 Australia; 7 Dementia Australia, Parkville, VIC Australia; 8 Curtin School of Allied Health, Curtin University, Kent Street, Bentley, WA Australia; 9 Brightwater Research Centre, Brightwater Care Group, Inglewood, WA Australia; 10 Walton Library, Faculty of Medical Sciences, Newcastle University, Newcastle Upon Tyne, UK; 11 Sax Institute, Sydney, Australia; 12 School of Population Health, Curtin University, Kent Street, Bentley, WA Australia; 13 Curtin enAble Institute, Curtin University, Kent Street, Bentley, WA Australia; 14 Mental Health, Alcohol, Substance Use, and Tobacco Research Unit, South African Medical Research Council, Parow, Cape Town South Africa; 15 West Australian Country Health Service and Curtin University Research and Innovation Alliance, Perth, WA Australia; 16 Department of Clinical Psychology and Psychobiology, University of Barcelona, Barcelona, Spain; 17 School of Psychology, University of Surrey, Guildford, UK; 18 Nutrition & Health Innovation Research Institute, Edith Cowan University, Perth, Australia; 19 Medical School, The University of Western Australia, Perth, Australia; 20 Population Health Sciences Institute, Newcastle University, Newcastle Upon Tyne, UK; 21 School of Social Sciences, Monash University, Melbourne, Australia

**Keywords:** Dementia, Risk prediction, Prevention, External validation, Systematic review

## Abstract

**Background:**

Data on the external validation of current dementia risk prediction models has not yet been systematically synthesised. This systematic review and meta-analysis collated results from three previous reviews to evaluate the predictive discriminative performance of dementia risk models when validated in population-based settings.

**Methods:**

Embase (via Ovid), Medline (via Ovid), Scopus, and Web of Science were searched from inception to June 2022 with an updated search conducted up to November 2024. Included studies (1) had a population-based cohort design; (2) assessed incident late-life (i.e. ≥ 60 years) dementia; and (3) reported predictive performance of at least one dementia risk prediction model in an independent validation sample. Information on study characteristics, dementia outcomes, prediction models (including whether they were fully validated [all original variables available and mapped] or partially validated [one or more variables missing or substituted]), and their discriminative performance were extracted in duplicate. Discrimination, quantified by the area under the receiver operating characteristic curve (AUC) or *c*-statistic, was pooled across studies using a random-effects model. Models were stratified by validation type: fully versus partially validated.

**Results:**

Thirty-six studies were included. Seventeen studies undertook full validation (14 unique prediction models) and were included in the meta-analysis. Predictor count ranged from one to 57. For all-cause dementia, RADaR showed the highest performance (*c*-statistic = 0.83, 95%CI: 0.80–0.86; *n* = 2 validations), followed by eRADAR (*c*-statistic = 0.81, 95%CI: 0.75–0.85; *n* = 2 validations). The BDSI model had the most validations (all-cause dementia *c*-statistic = 0.72, 95%CI: 0.69–0.75; *n* = 13 validations; and Alzheimer’s disease *c*-statistic = 0.74, 95%CI: 0.61–0.87; *n* = 2 validations) and performed similarly across high- and middle-income counties. Most validations (76%) were conducted in high-income countries, with 24% in upper-middle income countries. Considerable variation in heterogeneity was observed across models (*I*^2^ values ranging from 0 to 99%).

**Conclusions:**

Several dementia risk prediction models demonstrate moderate to high external validity. The BDSI model, tested across multiple settings and dementia outcomes, showed promising generalisability. However, the limited number of fully validated models and scarcity of studies in low-income country settings highlight the need for further research on feasibility, resource requirements, and cost-effectiveness before clinical adoption.

**Supplementary Information:**

The online version contains supplementary material available at 10.1186/s12916-026-04652-y.

## Background

Dementia is a global public health priority affecting over 57 million people worldwide [1]. Without a cure, effective prevention requires understanding dementia risk factors across diverse populations, demographics, and environments to develop targeted interventions that reduce global disease burden [2]. This knowledge could inform clinical decision support tools and guide healthcare providers, consumers, and policymakers in developing effective brain health promotion strategies.

Over 100 different dementia risk prediction models exist [3–5]. However, most models have often low (*c*-statistic < 0.60) and heterogenous predictive accuracy (*c*-statistic range: < 0.50 to 0.90) and have almost exclusively been developed in high-income White populations. Further, they show mixed transportability (i.e. external validity) and calibration results [4–6]. This makes recommending robust models for diverse healthcare, community, or research settings challenging.

Comprehensive validation across diverse populations is essential to improve model applicability. These studies can identify whether models need refinement (adjusting risk weightings depending on sample characteristics such as age) or re-development (e.g. creation of new predictive models in settings where current models are found to have insufficient performance). This systematic review and meta-analysis evaluates performance of externally validated dementia risk models. We present findings from all validation studies but focus our analysis on fully validated models (where all original variables were available and mapped) rather than partially validated ones (where one or more variables were missing or substituted). This approach ensures reliability, comparability, and minimises bias when drawing conclusions about model performance. External validation assessment is critical for determining which models can generalise across populations, a pre-requisite for implementation decisions. Understanding which models maintain discriminative performance and external validity across settings provides the foundation for evidence-based risk reduction strategies. This analysis helps identify which models warrant further testing for implementation and which ones require modification before clinical adoption.

## Methods

This study was conducted and reported according to Preferred Reporting Items for Systematic Review and Meta-Analysis (PRISMA) guidelines [7]. The data analysed has been collated from three published systematic reviews conducted by the same research team covering all dementia risk model development and validation literature from database inception to 22nd June 2022 [3–5]. The protocol was registered on PROSPERO (Reference CRD42022320630). An updated search was run on 16th November 2024 using the same search strategy and methodology to capture recent validation studies published after our original systematic reviews were completed.

### Literature search and study selection

Embase (via Ovid), Medline (via Ovid), Scopus, and Web of Science were searched using terms related to dementia, prediction, and model performance metrics (see Additional file 1: Table S1 for full strategy). No language or publication type restrictions were applied. Search results were de-duplicated in Endnote. We supplemented electronic searches with citation chasing by hand-searching reference lists of included studies. Title, abstract, and full-text screening was conducted independently by multiple authors (BS, JB, SS, CB) using the Covidence platform, with discrepancies resolved through discussion until consensus was reached.

### Eligibility criteria

#### Inclusion criteria

In all reviews, articles were included if the study sample was population-based and a predictive model for late-life (i.e. ≥ 60 years) incident dementia (all-cause and/or dementia subtypes) was reported as the outcome. We included population-based studies of late-life (≥ 60 years) incident dementia that met four criteria: (1) conducted external validation of a previously published risk prediction model in a different dataset from that used for model development; (2) explicitly specified which model variables were mapped, clearly indicating whether validation was full (all original variables available and mapped) or partial (one or more variables missing or substituted); (3) reported discriminative accuracy using the AUC or *c*-statistic; (4) studies that stratified by ethnicity were included. Studies that tested discriminative performance in general populations without health status restrictions were also included. A dementia risk prediction model was defined as a statistical algorithm specifically designed and validated to estimate the probability of developing an event (e.g. dementia including all-cause and/or its subtypes) within a defined time, incorporating risk factors with assigned weights or coefficients. Models originally developed for predicting non-dementia outcomes (e.g. cardiovascular disease, diabetes) and that were subsequently adapted and validated for dementia risk prediction were also included. Models that were not explicitly developed for risk prediction purposes were excluded.

#### Exclusion criteria

Studies validating models in specific disease populations (diabetes, stroke, heart failure) were excluded as these have been previously published [8]. Studies that had only undertaken internal validation (e.g. developed and tested a model in the same data resource using techniques such as random split-sampling, bootstrapping, or standard cross-validation [i.e. using k-folds]) were excluded. In addition, studies were excluded if 95% confidence intervals for *c*-statistics were not reported or if only partial validation was performed (one or more predictor variables missing). We also excluded studies that undertook full validation with additional variables (e.g. hybrid models combining original model variables with new predictors such as mapping the Cardiovascular Risk Factors, Ageing, and Incidence of Dementia (CAIDE) score with the addition of traumatic brain injury [9] or inflammation-related biomarkers [10]) as this approach may create hybrid models that differ from the original published versions and make it challenging to assess the original model’s transportability. When multiple publications validated the same model using identical data sources, only the validation with the largest sample size was included. For studies reporting multiple follow-up periods, 5-year follow-up estimates were selected as these were consistently presented across studies, enabling better *c*-statistic comparability. Studies that undertook temporal validation (e.g. tested a model’s performance and predictive discriminative performance over time such as developing a model in a historical dataset and undertaking validation in a dataset collected after the development data collection period) were included. Further, studies that undertook geographically based cross-site validation in multi-site datasets (e.g. model developed in one city and tested in another within the same cohort study) were excluded.

### Data extraction

Key information was extracted from each study: author, publication year, data sources, country, sample size, dementia cases, follow-up duration, models evaluated, and performance metrics. For multiple-model validations, separate extractions were performed. Data were extracted by two authors (BS, MS) and independently verified by four others (EB, JB, SS, CB). Whether validations were full or partial was documented, as this distinction affects performance interpretation. Discrepancies were resolved via discussion.

### Risk of bias assessment

The methodological quality and risk of bias of included studies were assessed using the Prediction Model Risk of Bias Assessment Tool (PROBAST). PROBAST evaluates four key domains: participants, predictors, outcome, and analysis, with each domain assessed for risk of bias (ROB) and concerns regarding applicability. One reviewer assessed each study (MS), which was and independently checked by a second reviewer (BS), with discrepancies resolved through discussion. Studies were classified as having low (+), high (−), or unclear (?) risk of bias [11].

### Statistical analysis

A narrative synthesis was initially conducted to describe the study populations and risk models validated, along with their estimated *c*-statistic/AUC values. Using the *c*-statistic/AUC values, model predictive performance was classified as poor (0.50–0.69), acceptable (0.70–0.79), or excellent (≥ 0.80) [12].

For meta-analysis, results were pooled when a risk model was fully validated (all component predictors mapped) by two or more unique studies. Sensitivity analyses were conducted where sufficient data existed, including only studies with characteristics closely matching validation samples (age, follow-up time, and sex distribution). The analysis was performed using the Meta-Analysis module of IBM SPSS Statistics for Windows, version 28.0 (IBM Corp., Armonk, NY, 2021). Pooled *c*-statistics with 95%CIs were calculated using random-effect models. Separate meta-analyses were conducted for each dementia risk model, stratified by dementia type (all-cause, Alzheimer’s disease (AD), and vascular dementia (VaD)) and country income level (high vs. middle vs. low income; as defined by World Bank Group country classifications by income level [13]). Income level subgroup differences were evaluated using the *Q*-test. Forest plots were generated for all models, stratified by dementia type. Heterogeneity was assessed via chi-squared test and quantified using *I*^2^ statistic. Publication bias was examined with funnel plots and Egger’s regression test. Significance was set at *p* < 0.05.

## Results

Across the three published reviews, 7322 articles (*n* = 1255 from the first review [4], *n* = 1234 from the second review [5], and *n* = 4833 from the third review [3]) were identified after excluding duplicates of which *n* = 112 met the inclusion criteria. From these articles, *n* = 19 had undertaken external validation analyses and were included in this review (two studies from our second review [14, 15] and 17 studies from our third review [16–32]). The updated literature search identified a further *n* = 3786 articles of which 17 [9, 10, 33–47] met the eligibility criteria (including one study identified from backward citation chasing). Therefore, this analysis is based on *n* = 36 studies (see Additional file 1: Fig. S1 for the PRISMA flow diagram).

### Study characteristics

Table 1 presents the dataset characteristics and prediction models that were validated across the included studies. Studies were published between 2014 and 2024, almost exclusively (87%) in high-income countries including the United Kingdom (UK), America (USA), Sweden, France, Iceland, Netherlands, Finland, Canada, Germany, and Italy. Five studies were undertaken in upper-middle income countries, and no studies were conducted in low- or lower-middle income countries (LMICs). One study was conducted in men only [39], and one in women only [34].

**Table 1:** Characteristics of datasets used to validate the prediction models in the included studies (alphabetical order)

Reference	Study (country)	Validation sample size	*N* female (%)	Age statistics at baseline (e.g. mean [SD], median [IQR])	Baseline sample age range	Outcome	Follow-up in years (range)	Models validated	Validation type
Anatürk 2023 [33]	UKB (UK) WHII (UK)	UKB test: 44,151 WHII: 2934	UKB test: not reported WHII: 834 (28.4%)	UKB: not reported for test sample WHII: median = 57 (IQR = 10)	UKB: 40–73 WHII: 35–55	All-cause	UKB: 14 years WHII: 16 years	ANU-ADRI [48] CAIDE [49] DRS-young [31] UKBDRS [33] UKBDRS + APOE [33]	External
Anstey 2014 [14]	MAP (USA) KP (Sweden) CVHS (USA)	MAP: 903 KP: 905 CVHS: 2496	MAP: 75.2% KP: 75% CVHS: 59.1%	MAP: 79.8 (7.4) KP: 81.5 (5.0) CVHS: 72.3 (4.9)	MAP: 54–100 KP: 74–100 CVHS: 62–95	All-cause and AD	MAP: mean = 3.5 years (SD = 3.0) KP: mean = 6.0 years (SD = 5.7) CVHS: median = 6.0 years	ANU-ADRI [48] CAIDE [49]	External
Ben-Hassen 2022 [16]	3 C (France)	3953	2354 (59.5%)	Mean = 73.2 (SD = 5.0)	Not reported	All-cause	Median = 9.7 years (IQR: 5.6, 15.4)	CTSD [16]	External
Capuano 2022 [17]	ROS (USA) MARS (USA)	ROS: 1308 MARS: 998	ROS: 942 (72.0%) MARS: 780 (78.2%)	ROS: mean = 75.4 (SD = 7.2) MARS: mean = 73.2 (SD = 6.3)	Not reported	All-cause	Median = 10 years (IQR: 5, 16)	RADaR (full model) [17]	External
Capuano 2022 [17]	Combined data: MAP, ROS, and MARS (USA)	2357	Not reported for combined sample	Not reported for combined sample	< 80	All-cause	3 years	BDSI [50]	External
Casanova 2016 [18]	BLSA (USA) AGES-RS (Iceland)	BLSA: 200 AGES-RS: 192	BLSA: 94 (49.0%) AGES-RS: 109 (54.5%)	BLSA: 77.2 (SD = 6.6) AGES-RS: 78.2 (SD = 4.4)	Not reported	AD	Mean = 5.2 (SD = 0.25) for both cohorts	Plasma phospholipids (10-metabolite panel) [51]	External
Chen 2023 [34]	CLHLS [2014–2018] (China)	1060	100%	Mean = 83.0 (SD = 8.5)	62–112	Severe cognitive impairment like dementia (Chinese MMSE < 18)	Median = 4.1 (IQR: 3.5, 4.3)	CLHLS Risk Model-Illiterate Women [34]	Temporal
Chouraki 2016 [19]	3 C (France) ACT (USA) AGES (Iceland) CHS (USA) FHS (USA) ROSMAP (USA) Rotterdam (Netherlands) WHICAP (USA)	3C: 6079 ACT: 2110 AGES: 2553 CHS: 1998 FHS: 1757 ROSMAP: 1262 Rotterdam: 3334 WHICAP: 594	3C: 60.8% ACT: 56.3% AGES: 59.6% CHS: 62.2% FHS: 57.3% ROSMAP: 69.5% Rotterdam: 60.3% WHICAP: 61.6%	3C: mean = 74.2 (SD = 5.5) ACT: mean = 75.6 (SD = 6.5) AGES: mean = 75.5 (SD = 5.1) CHS: mean = 74.8 (SD = 4.7) FHS: mean = 76.1 (SD = 7.4) ROSMAP: mean = 78.4 (SD = 7.1) Rotterdam: mean = 74.0 (SD = 6.5) WHICAP: mean = 76.7 (SD = 6.8)	≥ 65	AD	5, 6, 7, and 8 years 3C: mean = 6.3 (SD = 3.1) ACT: mean = 8.0 (SD = 5.0) AGES: mean = 5.9 (SD = 2.2) CHS: mean = 6.5 (SD = 3.5) FHS: mean = 7.6 (SD = 4.5) ROSMAP: mean = 8.0 (SD = 4.7) Rotterdam: mean = 10.9 (SD = 5.8) WHICAP: mean = 6.2 (SD = 4.6)	Demographic + APOE [19] GRS-19 [19]	External
Coley 2023 [35]	KPWA (USA) UCSF (USA)	KPWA: 129,315 UCSF: 13,444	KPWA: 9721 (60.2%) UCSF: 27,686 (58.5%)	KPWA: mean = 73.5 (SD = 7.3) UCSF: mean = 73.7 (SD = 7.2)	≥ 65	All-cause	KPWA: 10 years UCSF: 5 years	eRADAR (full model) [52]	External
Deckers 2020 [20]	CAIDE (Finland)	1024 (midlife) 604 (late life)	Not reported for the baseline sample combined	Not reported for the baseline sample combined	Midlife (40–50) Late life (65–79)	All-cause	30 years	LIBRA [53]	External
Dhana 2024 [42]	CHAP (USA)	2130 (1159 Black or African American; 971 White)	Black or African American: 734 (63.3%) White: 602 (62.0%)	Black or African American: 71.7 (SD = 5.1) White: 74.8 (SD = 6.3)	≥ 65	AD	5, 6, 10, 15, and 20 years	ANU-ADRI [48] BDSI [50] CAIDE [49] CAIDE + APOE [49] DRS-young [31] DRS-old [31]	External
Downer 2016 [21]	MHAS (Mexico)	3002	1753 (58.4%)	Not reported	≥ 60	All-cause	11 years	BDSI [50]	External
Exalto 2014 [15]	KPNC	9480	5226 (55.1%)	Mean = 46.1 (SD = 4.3)	40–55	All-cause	Mean = 36.1 years	CAIDE [49]	External
Fayosse 2020 [22]	WHII (UK)	7553	2323 (30.8%)	Mean = 50 (no SD)	39–63	ICD-10	Mean = 23.5 years	CAIDE [49] FINSRISC [54] FRS [55]	External
Fisher 2021 [23]	Ontario CCHS [2009/2010 and 2011/2012] (Canada)	27,721	15,930 (57.5%)	Male: median = 66.0 (IQR: 60.0, 74.0) Female: median = 67.0 (IQR = 61.0, 76.0)	≥ 55	All-cause	5 years	DemPoRT [23]	Temporal
Fung 2024 [43]	HK-MAPS (Hong Kong)	383	191 (49.9%)	Mean = 69.9 (SD = 6.4)	≥ 60	All-cause (cognitive *z*-score criteria)	Mean = 5.4 (SD = 0.3)	CARS [43]	External
Hu 2022 [24]	CLHLS [2002–2008] (China)	9240	4849 (52.5%)	Not reported for validation sample separately	≥ 65	Severe cognitive impairment like dementia (Chinese MMSE < 18)	6 years	CLHLS Risk Model [24]	Temporal
Huque 2023 [9]	MAP (USA) CHS-CS (USA) HRS-ADAMS (USA)	MAP: 2184 HRS-ADAMS: 548 CHS-CS: 3375	MAP: 1606 (73.5%) HRS-ADAMS: 288 (52.5%) CHS-CS: 1994 (59.1%)	Mean age (SD) MAP: mean = 80.0 (SD = 7.6) HRS-ADAMS: mean = 79.5 (SD = 6.3) CHS-CS: mean = 74.8 (SD = 4.9)	MAP: 54–100 HRS-ADAMS: 70–103 CHS-CS: 65–97	All-cause and AD	MAP: median = 5.0 years (IQR: 1.0, 2.0) HRS-ADAMS: median = 5 years (IQR: 2.0, 6.0) CHS-CS: 6.0 years (IQR: 0.2, 7.7)	ANU-ADRI [48] CAIDE [49] CogDrisk [56] CogDrisk-AD [56] LIBRA [53] LIBRA-Modified	External
John 2022 [25]	MDCR (USA) IQGER (Germany) OPSES (USA) OPEHR (USA) CPRD (UK) IPCI (Netherlands) IMRD (UK)	MDCR: 10 million IQGER: 30 million OPSES: 85 million OPEHR: 94 million CPRD: 13 million IPCI: 2.5 million IMRD: 18 million	MDCR: not reported IQGER: not reported OPSES: not reported OPEHR: not reported CPRD: not reported IPCI: not reported IMRD: not reported	MDCR: not reported IQGER not reported OPSES: not reported OPEHR: not reported CPRD: not reported IPCI: not reported IMRD: not reported	DRS-young (sample restricted to age 60–79 years) RxDx-Dementia Risk Index (sample restricted to 45–89 years) Nori-ADRD Score (sample restricted to ≥ 45 years)	All-cause	5 years (all datasets)	DRS-young [31] RxDx-Dementia Risk Index [57] Nori-ADRD Score [58]	External
John 2024 [44]	MDCR (USA) IQGER (Germany) OPSES (USA) IPCI (Netherlands)	MDCR: 999,480 IQGER: 946,900 OPSES: 999,439 IPCI: 186,767	MDCR: 533,879 (53.4%) IQGER: 534,014 (56.4%) OPSES: 537,146 (53.7%) IPCI: 100,439 (53.8%)	Not reported	55–84	All-cause	5 years (all datasets)	OPERH [44]	External and temporal
Kivimäki 2023 [36]	UKB (UK) WHII (UK)	UKB: 465,929 WHII 4865	UKB: 252,778 (54.3%) WHII: 1342 (27.6%)	UKB: mean = 56.5 (SD = 8.1) WHII: mean = 54.9 (SD = 5.9)	UKB: 38–73 years Whitehall: 45–69 years	All-cause	UKB: 10 years Whitehall: 20 years	CAIDE [49] CAIDE + APOE [49] BDSI [50] ANU-ADRI [48]	External
Kootar 2023 [37]	SNAC-K (Sweden) HRS-ADAMS (USA) CHS-CS (USA) MAP (USA)	SNAC-K: 3122 HRS-ADAMS: 856 CHS-CS: 3375 MAP: 2184	SNAC-K: 63.4% HRS-ADAMS: 58.5% CHS-CS: 59.1% MAP: 73.5%	SNAC-K: mean = 73.6 (SD = 10.7) HRS-ADAMS: mean = 81.6 (SD = 7.1) CHS-CS: mean = 74.8 (SD = 4.9) MAP: mean = 80.0 (SD = 7.6)	SNAC-K: 60–104 HRS-ADAMS: 70–110 CHS-CS: 65–97 MAP: 54–100	All-cause and AD	SNAC-K: 9 years HRS-ADAMS: 7 years CHS-CS: 8 years MAP: 22 years	CogDrisk [56] CogDrisk-AD [56]	External
Licher 2018 [27]	Rotterdam Study (Netherlands)	6667	3787 (56.8%)	Mean = 69.1 (SD = 8.2)	≥ 55	All-cause and AD	10 years	CAIDE [49] BDSI [50] ANU-ADRI [48] DRS-young [31] DRS-old [31]	External
Licher 2019 [26]	EPOZ (Netherlands)	514	274 (53.3%)	Mean = 70.8 (SD = 6.5)	60–90	All-cause	Median = 9.5 years (IQR 7.6, 11.4)	Basic-DRM [26] Basic-DRM (Extended) [26]	External
Mura 2017 [46]	3C-Montpellier centre (France)	Not reported for Montpellier centre only	Not reported for Montpellier centre only	Not reported for Montpellier centre only	≥ 65	All-cause and AD	3 and 5 years	Demographic-Cognition Model_1 [46] Demographic-Cognition Model_2 [46] Demographic-Cognition Model_3 [46] Demographic-Cognition Model_4 [46]	Cross-validation
Reeves 2024 [45]	CPRD Gold (158 GP practices)	419,126	215,802 (51.5%)	Mean = 67.9 (SD = 6.4)	60–89	All-cause	5 years	DEMRisk-young [45] DEMRisk-old [45] DRS-young [31] DRS-old [31]	External and cross-validation
Schiepers 2018 [28]	MAAS (Netherlands)	949	466 (49.1%)	Mean = 65.0 (SD = 8.7)	50–81	All-cause	16 years	LIBRA [53]	External
Shang 2022 [38]	UKB (UK)	471,485	54.5%	Mean = 56.8 (SD = 8.0)	38–73	All-cause	Median = 11.9 years	CAIDE [49] FRS [55]	External
Stephan 2020 [32]	10/66 Study (China, Cuba, Dominican Republic, Mexico, Peru, Puerto Rico, and Venezuela)	11,143	6973 (62.6%)	Mean age: 73.8 (SD = 6.6)	≥ 65	All-cause	Mean = 3.8 (SD = 1.3)	AgeCoDe [59] ANU-ADRI [48] Basic-DRM [26] BDSI [50] CAIDE [49]	External
Stephan 2023 [39]	AGES-Reykjavik Study (Iceland)	4733	0% (males only)	Mean = 51.4 (SD = 7.0)	45–68	All-cause, AD, and VaD	~ 30 years (range: 26–4499 days; mean = 2484 days [SD = 992])	HAAS (NIA-Reagan) [39] HAAS (NP) [39] HAAS (NFT) [39] HAAS (MVL) [39] HAAS (LI) [39] HAAS (MIXED) [39]	External
Trares 2024 [47]	ESTHER Study (Germany)	5360	Not reported for whole baseline sample	Not reported for whole baseline sample	50–75	All-cause, AD, and VaD	Mean = 14.8 (SD = 3.5)	CAIDE [49] CAIDE + APOE [49]	External
Trares 2024 [10]	ESTHER Study (Germany)	1918	Not reported for whole baseline sample	Not reported for whole baseline sample	50–75	All-cause, AD, and VaD	Median = 16.3 (IQR: 13.5, 17.0)	CAIDE [49] CAIDE + APOE [49]	External
Vonk 2021 [29]	AGES-Reykjavik Study (Iceland)	5343	3097 (58.0%)	Mean = 76.6 (SD = 5.7)	66–98	All-cause and AD	All-cause mean = 8.4 AD mean = 8.6	ANU-ADRI [48] Basic-DRM [26] BDSI [50] CSHA Derived Score [60] Demographic-Cognition Model_4 [29] FDRS [61] LLDRI [62] MADeN [63] Tierney 2005 [64] Tierney 2010 [65] Verhaaren-2 [66] Verhaaren-3 [66]	External
Vos 2017 [30]	DESCRIPA Study using 6 cohorts: (1) GPPSW (Sweden); (2) GH85 (Sweden); (3) ILSA (Italy); (4) LASA (Netherlands); (5) MAAS (Netherlands); and (6) PAQUID (France)	9387	5141 (54.8%)	Mean = 72.9 (SD = 7.3)	55–97	All-cause	Mean = 7.2 years (SD = 3.6; range: 1–16 years)	LIBRA [53]	
Walters 2016 [31]	THIN (UK)	264,224 (226,140 were 60–79 years and 38,084 were 80–95 years)	60–79: 117,032 (51.8%) 80–95: 25,067 (65.8%)	60–79: mean = 65.6 (SD = 6.1) 80–95: mean = 84.9 (SD = 4.0)	60–95	All-cause	5 years	DRS-young DRS-old	Cross-validation
Yang 2022 [40]	ROS (USA)	1103	Not reported for ROS separately	Not reported for ROS separately	≥ 65	AD	3 and 5 years [median = 8 years (SD = 5.4; range: 2–26 years)]	Model A [40] Model B [40] Model C [40] Model D [40]	External
Zheng 2023 [41]	UKB (UK)	429,033	53.8%	Mean = 57.1 (SD = 8.1)	38–79	All-cause	Median = 12.8 years	CAIDE [49] SCORE [67] SCORE2 [68]/SCORE-OP [69]	External

Acronyms: * 3 C *French Three City Study, *ACT *Adult Changes in Thought, *AD *Alzheimer’s disease, *AGES *Age, Gene/Environment Susceptibility study, *AGES-RS *Gene/Environment Susceptibility-Reykjavik Study, *ANU-ADRI *Australian National University Alzheimer’s Disease Risk Index, *APOE *apolipoprotein gene, *BDSI *Brief Dementia Screening Indicator, *BLSA *Baltimore Longitudinal Study of Aging, *CAIDE *Cardiovascular Risk Factors, Aging and Dementia Study Risk Score, *CCHS *Canadian Community Health Survey, *CHS *Cardiovascular Health Study, *CHAP *Chicago Health and Aging Project, *CHS-CS *Cardiovascular Health Study Cognition Study, *CLHLS *Chinese Longitudinal Healthy Longevity Survey, *CPRD *Clinical Practice Research Datalink, *CTDS *Cognitive Tests and Dependency Scale, *CVHS *Cardiovascular Health Cognition Study, *DemPoRT *Dementia Population Risk Tool, *DRS *Dementia Risk Score, *EPOZ *Epidemiological Prevention Study of Zoetermeer, *eRADAR *Electronic health record (EHR) Risk of Alzheimer’s and Dementia Assessment Rule, *FHS *Framingham Heart Study, *FINDRISC *Finnish Diabetes Risk Score, *FRS *Framingham Cardiovascular Risk Score, *GH85 *Gerontological and Geriatric Population Study of 85-year-olds, *GPPSW *Swedish Prospective Population Study of Women, *HAAS *Honolulu Asia Aging Study, *HRS-ADAMS *Health and Retirement Study–Aging, Demographics and Memory Study; *ILSA *The Italian Longitudinal Study of Aging, *IMRD *Iqvia Medical Research Database (incorporating The Health Improvement Network: THIN), *IPCI* Integrated Primary Care Information, *IQGER *Iqvia Germany DA, *IQR *interquartile range, *KP *Kungsholmen Project, *KPNC *Kaiser Permanente Medical Care Program of Northern California, *KPWA *Kaiser Permanente Washington, *LASA *Dutch Longitudinal Aging Study Amsterdam, *LI *lacunar infarcts, *LIBRA *Lifestyle for BRAin Health score, *MAAS *Maastricht Ageing Study, *MAP *Rush Memory and Aging Study, *MARS *Minority Aging Research Study, *MDCR *IBM MarketScan® Medicare Supplemental Database, *MHAS *Mexican Health and Aging Study, *MVL *microvascular lesions, *MIXED *combined Alzheimer’s and vascular neuropathology, *NAVD *non-Alzheimer non-vascular dementia, *NFT *neurofibrillary tangles, *NHIRD *National Health Insurance Research Database, *NIA-Reagan *National Institute on Aging (NIA) and the Ronald and Nancy Reagan Institute of the Alzheimer’s Association neuropathology criteria for Alzheimer’s disease, *NP *neuritic plaques, *OPEHR *External: Optum® de-identified Electronic Health Record dataset, *OPSES *Optum’s de-identified Clinformatics® Data Mart Database, *PAQUID *French Personnes Agees QUID study, *RADaR *Rapid Assessment of Dementia Risk, *ROS *Religious Order Study, *ROSMAP *Religious Order Study/Memory and Aging Project, *SCORE *Systematic COronary Risk Evaluation model, *SCORE2 *Updated Systematic COronary Risk Evaluation model, *SCORE2-OP *Systematic COronary Risk Evaluation (SCORE2)-Older Persons (≥70 years), *SNAC-K *The Swedish National study on Aging and Care in Kungsholmen, *THIN *The Health Improvement Network, *UCSF *University of California San Francisco Health, *UK *United Kingdom, *UKB *UK Biobank, *UKBDRS *UK Biobank Dementia Risk Score, *UKBDRS *UK Biobank Dementia Risk Score + Apolipoprotein e4 allele status, *USA *United States of America, *VaD *vascular dementia, *WHICAP *Washington Heights-Inwood Community Aging Project, *WHII *Whitehall II Study

### Risk of bias assessment

Risk of bias assessment revealed variable methodological quality across the 36 included studies. Most studies demonstrated low risk of bias for participant selection and predictor measurement domains. However, concerns were more frequent in the analysis domain (7 studies) [16, 22, 25, 28, 32, 35, 36, 43] and outcome measurement (2 studies) [20, 31]. Overall risk of bias was high in 5 studies [16, 25, 28, 32, 36] and unclear in 6 studies [20, 22, 31, 35, 37, 43], while applicability concerns were generally lower across all domains (Additional file 1: Table S2).

#### Model performance

Full details of the validated models and their performance in the development data set are in Additional file 1: Table S3. Overall, 56 models have been externally validated either fully or partially. Models incorporated a variety of predictors (e.g. demographic, health, lifestyle, genetic) ranging from a single predictor model (i.e. composite cognition score) [40] to a model incorporating 57 variables (i.e. 55 non-cognitive covariates, Mini Mental State Examination [MMSE] plus a composite cognition score) [40]. Most models (*n* = 47 out of 56) were originally developed for predicting all-cause dementia and its subtypes (AD and VaD), with the remaining originally developed to predict cardiovascular events (including fatal events and atherosclerotic CVD; *n* = 4 models [55, 67–69]), dementia-related brain neuropathology (*n* = 6 models [39]), or type II diabetes (*n* = 1 model [54]). Predictive discriminative performance of the models in the development sample ranged from poor (*c*-statistic = 0.49; 95%CI: 0.44–0.54 [39]) to excellent (*c*-statistic = 0.96 [16]). Full details of the performance of each model in the validation samples (both full and partial) are in Additional file 1: Table S4.

#### Meta-analysis

Seventeen studies reporting full validation data for 14 unique risk prediction models (validated in at least two independent studies) were included in meta-analyses. These comprised 98 individual validation analyses: 87 for all-cause dementia, seven for AD, and four for VaD. Most analyses (80) were conducted in high-income countries, with 23 from upper-middle income countries. No analyses from low-income countries were available (Table 2).

**Table 2:** Meta-analysis estimates of the predictive performance of risk models for all-cause dementia, Alzheimer’s disease (AD), and vascular dementia (VaD). The analysis provided overall estimates across all studies and further stratified results by country income classification (high or middle) based on global economic rankings

Type	Prediction model	N*	*c*-statistic	95%CI		*p* value	*I*^2^
				Lower	Upper		
All-cause	AgeCoDe [59]	7	0.66	0.62	0.71	< 0.001	0.80
	High	1	0.71	0.66	0.75	< 0.001	–
	Middle	6	0.65	0.61	0.70	< 0.001	0.80
	Basic-DRM [26]	9	0.72	0.70	0.75	< 0.001	0.72
	High	3	0.74	0.72	0.75	< 0.001	0
	Middle	6	0.72	0.68	0.75	< 0.001	0.77
	BDSI [50]	13	0.72	0.69	0.75	< 0.001	0.87
	High	7	0.74	0.70	0.77	< 0.001	0.93
	Middle	6	0.68	0.64	0.72	< 0.001	0.66
	CAIDE [49]	12	0.60	0.55	0.64	< 0.001	0.95
	High	7	0.62	0.55	0.69	< 0.001	0.98
	Middle	5	0.56	0.53	0.58	< 0.001	0.20
	CAIDE + APOE [49]	5	0.68	0.60	0.76	< 0.001	0.97
	Demographic + APOE [19]	8	0.79	0.76	0.81	< 0.001	0.74
	DRS-young (60–79) [31]	5	0.79	0.75	0.82	< 0.001	0.96
	DRS-old (80–95) [31]	3	0.58	0.54	0.61	< 0.001	0.93
	eRADAR [52]	2	0.81	0.75	0.85	< 0.001	0.92
	FRS [55]	2	0.72	0.71	0.73	< 0.001	0
	GRS-19 [19]	5	0.78	0.75	0.80	< 0.001	0.52
	Nori-ADRD [58]	7	0.66	0.64	0.68	< 0.001	0.94
	RADaR [17]	2	0.83	0.80	0.86	< 0.001	0
	RxDx-DRI [57]	7	0.74	0.71	0.77	< 0.001	0.99
AD	BDSI [50]	2	0.74	0.61	0.87	< 0.001	0.97
	CAIDE [49]	3	0.66	0.53	0.78	< 0.001	0.98
	CAIDE + APOE [49]	2	0.77	0.70	0.83	< 0.001	0.72
VaD	CAIDE [49]	2	0.72	0.68	0.78	< 0.001	0.75
	CAIDE + APOE [49]	2	0.76	0.74	0.78	< 0.001	0

Acronyms: *N *number of validations, *95%CI *95% confidence intervals, *ADRD *Alzheimer’s disease related dementias, *AgeCoDe *Ageing, Cognition, and Dementia, *DRM *dementia risk model, *BDSI *Brief Dementia Screening Index, *CAIDE *Cardiovascular Risk Factors, Aging, and Incidence of Dementia, *DRS *Dementia Risk Score, *eRADAR *electronic Risk of Alzheimer’s and Dementia Assessment Rule, *GRS-19 *Genetic Risk Score, *RxDx-DRI *disease conditions (Dx), prescription drugs (Rx) Dementia Risk Index, *I*^*2*^ heterogeneity index. Between-group comparison of the *c*-statistic estimates from studies conducted in countries with different economic development (high vs. middle) was performed (no significant differences between groups were observed)

*Please see Appendix 4 for which studies were included in the meta-analysis

For all-cause dementia, five models had a pooled *c*-statistic > 0.75 including the GRS-19 [19], DRS-young [31], Demographic + APOE [19], RADaR [17], and eRADAR [52]. The RADaR model demonstrated the highest predictive performance (pooled *c*-statistic = 0.83, 95%CI: 0.80–0.86; *n* = 2 validations), followed by the eRADAR model (pooled *c*-statistic = 0.81, 95%CI: 0.75–0.85; *n* = 2 validations). The greatest number of validations was performed for the BDSI model (pooled *c*-statistic = 0.72, 95%CI: 0.69–0.75; *n* = 13 validations). In contrast, the CAIDE model had the lowest performance (pooled *c*-statistic = 0.60, 95%CI: 0.55–0.64; *n* = 12 validations). However, the degree of heterogeneity across models varied from low (0% for RADaR [17], FRS [55]) to high (99% for RxDx-DRI [57]) (Table 2 and Additional file 1: Fig. S2).

For AD specific predictions, the pooled *c*-statistic estimates ranged from 0.66 (95%CI: 0.53–0.78; *n* = 3 validations) for the CAIDE [49] score to 0.77 (95%CI: 0.70–0.83; *n* = 2 validations) for the CAIDE + APOE [49] score, with moderate to high heterogeneity (*I*^2^ of 97% and 72%, respectively). For VaD specific predictions, the pooled *c*-statistic estimates ranged from 0.72 (95%CI: 0.68–0.78; *n* = 2 validations; *I*^2^ = 75%) for the CAIDE score to 0.76 (95%CI: 0.74–0.78; *n* = 2 validations; *I*^2^ = 0) for the CAIDE + APOE score (Table 2 and Additional file 1: Fig. S3). No publication bias was observed for the all-cause dementia and AD analyses; this could not be calculated for the VaD analysis as there were insufficient studies (Additional file 1: Fig. S4). Strong alignment in geographic region, age ranges, follow-up periods, and outcome measures was observed in DRS models (young and old variants), RADaR, and eRADAR. Moderate to significant mismatches in demographics, follow-up duration, or outcome definitions were found in other models between development and validation samples (Additional file 1: Table S5).

#### Country income level

External validation of four models (AgeCoDe [59], BASIC-DRM [26], BDSI [50], and CAIDE [49]) across different country income levels generally showed higher predictive discriminative performance in high-income vs. middle-income countries, though the difference was not significant for any model (Table 2).

## Discussion

This systematic review and meta-analysis examined the external validity of current dementia risk prediction models. Most models were found to have undergone only partial validation (> 50%), with none externally validated in low-income settings. Mixed external validity was observed among fully validated models, with 10 showing acceptable pooled discriminative performance (*c*-statistic > 0.70) for all-cause dementia. Modest performance was noted for the CAIDE [49] and DRS-old [31] models. Model discriminative performance was generally consistent across socioeconomic settings, with comparable performance in high- and middle-income countries.

This review focused primarily on objective assessment of discriminative performance (*c*-statistics) and calibration (i.e. how well predicted risks align with observed outcomes) but findings are also important for clinical implementation of risk prediction models. Calibration was assessed variably across included studies using calibration plots, Hosmer–Lemeshow tests, or other calibration statistics. High-performing models, such as RADaR, showed excellent discrimination but had limited calibration evidence, which requires careful consideration in the evaluation of model readiness for clinical implementation. The relatively short follow-up periods in some studies may overestimate model discriminative performance for dementia prediction. Longer follow-up periods allow for more incident cases and better assessment of model calibration over time. Models incorporating primarily non-modifiable risk factors (age, genetics, family history) may have limited utility for prevention-focused interventions, despite potentially high discriminative performance. In contrast, models incorporating modifiable risk factors (lifestyle, cardiovascular health, depression) may offer greater potential to inform public health prevention strategies.

The top four performing models, with the highest external validity (i.e. pooled *c*-statistic range 0.79–0.83) for predicting all-cause dementia, included the Demographic + APOE [19] (sociodemographic/genetic factors), DRS-young (60–79) (comprehensive health indicators) [31], eRADAR [52] (multidomain health and healthcare utilisation metrics), and RADaR [17] (cognitive/functional assessment) models. These models were categorised into three groups: (1) basic demographic/genetic models (Demographic + APOE); (2) comprehensive medical models (DRS-young, eRADAR); and (3) cognitive-focused models (RADaR), with some bridging multiple categories. Variation was observed in complexity (1–57 variables), predictor types (health status, genetics, cognition, healthcare utilisation), data requirements (routine versus specialised assessments), modifiable risk factors, and validation extent (2–13 validations). Demographic variables were most consistently utilised, with age included in all models and sex in all except RADaR. Further research is needed to evaluate implementation feasibility, resource requirements, and cost-effectiveness across diverse healthcare settings, particularly in resource-limited systems.

While the CAIDE [49] model demonstrated acceptable predictive discriminative performance in its development cohort (*c*-statistic = 0.77; 95%CI: 0.71–0.83), comprising middle-aged Finnish participants (*N* = 1409; age 39–64 years; mean follow-up = 20 years), validation studies report divergent results. External validation was poor in the 10/66 Study comprising data from six non-European middle-income countries: Cuba, the Dominican Republic, Mexico, Peru, Puerto Rico, and Venezuela [32]. Several methodological and contextual factors may explain model discriminative performance differences: (1) age distribution disparities between development and validation cohorts (middle-aged vs. ≥ 65 years), affecting risk factor prevalence and baseline hazards; (2) follow-up duration variations (20 years vs. 3–5 years), impacting event ascertainment and calibration; (3) differences in dementia diagnostic criteria, causing variability in case identification and outcome classification; and (4) population-specific risk factor associations due to environmental, healthcare, cultural, genetic, and lifestyle factors, affecting model transportability across diverse settings. Similar low performance (*c*-statistic = 0.54; 95%CI: 0.50–0.58) was observed in the Rotterdam Study [27], potentially attributable to disparities in the age structure (participants aged ≥ 55 years at baseline) and follow-up duration (5 years) between the development and validation samples. In contrast, external validation in comparable populations (i.e. UK Biobank and the Whitehall II Study), characterised by similar age distributions, follow-up periods, and sociocultural contexts, maintained robust predictive discriminative performance for both the CAIDE (pooled *c*-statistics ≥ 0.70 [22, 38]) and CAIDE + APOE (pooled *c*-statistic = 0.73 [36]) models. As such, the CAIDE model may need population-specific recalibration or modification to perform accurately in diverse settings, particularly in LMICs and populations outside the development sample age range (e.g. people aged ≥ 65 years, which is older than the 39–64 years age range of the development sample).

The DRS-old [31] model also performed poorly, with predictive discriminative performance just above chance even when validated in similar populations to the development cohort (i.e. Western-European, majority White participants, aged 80–95 years). These findings suggested that traditional risk factor-based prediction models may not be suitable for the oldest-old population [31]. Given the high baseline risk of dementia in this age group and complex interplay between age-related factors, a more immediate approach focused on identifying early signs of cognitive/functional decline, rather than long-term risk prediction may be more beneficial.

Limited studies have undertaken model external validation focused on dementia subtypes including AD and VaD, with sufficient data for meta-analyses for only three models (CAIDE [49], CAIDE + APOE [49], and BDSI [50]). The results suggest that the BDSI incorporating sociodemographic, clinical, and functional predictors maintains high predictive discriminative performance for both AD (pooled *c*-statistic = 0.74; 95%CI: 0.61–0.87) and all-cause dementia (pooled *c*-statistic = 0.77; 95%CI: 0.68–0.76 in high-income countries and pooled *c*-statistic = 0.69; 95%CI: 0.65–0.72 in middle-income countries). Performance comparable to development samples (*c*-statistic range: 0.68–0.78) was observed. The robust generalisability of the BDSI across samples (including varying income settings) and dementia outcomes raises questions about its potential for clinical implementation in people aged ≥ 65 years. Its predictor variables (age, education, weight, diabetes, stroke, functional status, depression) are typically available and inexpensive to collect in routine healthcare. Modifiable risk factors within the model provide potential intervention targets. However, clinical impact assessment across diverse healthcare settings would be needed before adaptation recommendations are made. High heterogeneity (*I*^2^ values) in validation results suggests significant performance variability across populations, requiring further research to determine underlying key factors.

The study has several strengths. It provides the first comprehensive systematic assessment of the external validity of multiple dementia risk prediction models across diverse populations, settings, and outcomes (all-cause dementia and AD). Further, sufficient data enabled meta-analysis of 14 unique risk prediction models. With over 100 different dementia risk models available [3–5], there is an urgent need to focus on testing generalisability and comparing model performance, rather than continuing to develop new models, to guide recommendations on selection for use in routine clinical practice and research settings across different populations. Several limitations were identified. First, few studies undertook full model validation, excluding many from meta-analyses, including the ANU-ADRI model (partially validated < 20 times). Selective validation of certain components may lead to incorrect performance assessment and limited cross-study comparison. Fully mapped models showed substantial heterogeneity in sample characteristics, outcomes, predictive variable measurement, and follow-up times between development and validation samples, which may influence whether they have meaningful generalisability. Partial validation models were excluded from main analyses as the substitution and/or omission of original variables may produce unreliable performance estimates and compromise cross-study comparability, which may prevent valid conclusions about the true external validity of the original prediction models [11, 70]. Second, despite moderate to high predictive performance in some models, significant performance variability across populations, contexts, and measurement protocols was indicated by substantial heterogeneity, warranting caution when generalising findings. Third, external validation in low-income countries was absent, with limited validation in middle-income settings, restricting understanding of model performance in resource-constrained healthcare systems with different risk profiles and healthcare access. Given high dementia rates in LMIC settings, research to identify at-risk individuals is urgently needed. Limited data allowed meta-analysis to be conducted for only three models for AD and two for VaD, limiting conclusions about performance across dementia subtypes. Fourth, generalisability may be limited by mismatches between development and validation samples. Only DRS models (young/old variants), RADaR, and eRADAR demonstrated strong alignment in key characteristics (geographic region, age ranges, follow-up duration, outcome measures).

The risk of bias assessment revealed methodological heterogeneity between the models that may explain some of the observed performance variability. Studies with high risk of bias in the analysis domain often lacked appropriate handling of missing data or used not appropriate statistical methods, which may have affected model performance estimates. Applicability concerns were less frequent than methodological issues but still highlight challenges in translating models across different healthcare settings and populations. These quality variations underscore the importance of standardised validation protocols and robust statistical methods in development and validation of dementia risk prediction models, which can impact on recommendations for clinical implementation across diverse populations and healthcare systems.

## Conclusions

To date, while dementia risk assessment tools are recommended for use in Brain Health clinics and used in research settings, several challenges remain to validation and widescale implementation in diverse settings. Despite promising results for some models, others exhibited only modest external validity, with consistently low *c*-statistics across different settings and population characteristics. Overall, of the models evaluated in the meta-analyses, the BDSI showed promising generalisability across different outcomes (all-cause and AD) and country income levels, incorporating readily available and modifiable clinical predictors. While other risk prediction models such as GRS-19 demonstrated higher AUC values comparable to the BDSI, their complex nature, requiring extensive genetic testing and specialised analysis, makes them substantially more difficult to incorporate into routine clinical practice, especially in low-resource LMICs. However, high heterogeneity in the validation results highlights the importance of context-specific testing and possible model recalibration to ensure applicability and reliability in diverse populations. Future research should aim to determine the sources of heterogeneity in model performance, establish standardised methods for model development and evaluation to facilitate meaningful comparisons, and address gaps, particularly in model development and validation in LMICs. The development of models that can be computed quickly and easily incorporated within routine healthcare consultations will be key. As the global burden of dementia continues to rise, accurate risk prediction models will be essential public health tools to enable early identification of high-risk individuals and drive the development of both individualised and population-based risk reduction and prevention strategies.

## Supplementary Information

Additional file 1: Figure S1. PRISMA flow diagram for updated systematic reviews which include searches of databases. Figure S2. Forest plots describing the performanceof fully externally validated risk models for predicting all-cause dementia. Figure S3. Forest plots describing the performanceof fully externally validated risk models for predicting Alzheimer’s disease and vascular dementia. Figure S4. Publication bias. Table S1. Example of the electronic search strategy. Table S2. Risk of bias and applicability assessment using the Prediction model Risk Of Bias Assessment Tool . Table S3. Details of risk models that have been externally validated. Table S4. Dementia risk prediction model external validation results. Table S5. Comparison of the development and external validation study characteristics and methods for variable mapping.

## Data Availability

The data that support the findings of this study are available from the corresponding author upon reasonable request.

## Abbreviations

3MS: Modified Mini-Mental State Examination
ACT: Adult Changes in Thought
AD: Alzheimer’s disease
ADRD: Alzheimer’s Disease and Related Disorders score
AF: Atrial fibrillation
AFR: African American
aMCI: Amnestic mild cognitive impairment
ANU-ADRI: Australian National University-Alzheimer’s Disease Risk Index
APOE: Apolipoprotein e4 allele status
ASCVD: Atherosclerotic cardiovascular disease
BDSI: Brief dementia screening indicator
BMI: Body mass index
*c*-statistic: Concordance statistic
CAIDE: Cardiovascular Risk Factors, Aging and Dementia Study
CARS: Cognitive ageing risk score
CHAP: Chicago Health and Aging Project
CHCS: Cardiovascular Health Cognition Study
CHD: Coronary heart disease
CHS: Cardiovascular Health Study
CSHA: Canadian Study of Health and Aging
CTDS: Cognitive Tests and Dependency Scale
CVD: Cardiovascular disease
DSM-III-R: Diagnostic and Statistical Manual (3rd Edition, revised)
DSM-IV: Diagnostic and Statistical Manual (4th Edition)
DSST: Digit Symbol Substitution
ESTHER Study: Epidemiologische Studie zu Chancen der Verhütung, Früherkennung und optimierten Therapie chronischer Erkrankungen in der älteren Bevölkerung
HER: Electronic health record
HK-MAPS: Hong Kong Memory and Ageing Prospective Study
FHS: Framingham Heart Study
FINDRISC: Finnish diabetes risk score
FRS: Framingham cardiovascular risk score
GRS-19: Genetic risk score including 19 SNPs
H-EPESE: Hispanic Established Populations for the Epidemiologic Study of the Elderly
HDL: High-density lipoprotein
HLA: Hispanic Latino American
HRS: Health and Retirement Study
IADL: Instrumental Activities of Daily Living
ICD-9-CM: International Classification of Diseases, Ninth Revision, Clinical Modification
IGAP: International Genomics of Alzheimer’s Project
IST: Isaac Set Test
KPNC: Kaiser Permanente Northern California
LI: Lacuna infarcts
LIBRA: LIfestyle for BRAin Health score
LLDRI: Late-life dementia risk index
LR: Logistic regression
MAP: Memory and Aging Project
MADeN: Mexican-American Dementia Nomogram
MMSE: Mini Mental State Examination
MRI: Magnetic resonance imaging
MVL: Microvascular lesions
N/A: Not applicable
NAVD: Non-AD or vascular dementia
NINDS-ADRDA: National Institute of Neurological and Communicative Disorders and Stroke and Alzheimer’s Disease and Related Disorders Association
NSAID: Non-steroidal anti-inflammatory drugs
OLDW: OptumLabs Data Warehouse
PAQUID: Personnes Agees QUID
PRS: Polygenic Risk Score
PVD: Peripheral vascular disease
RADaR: Rapid Assessment of Dementia Risk
RAVLT: Rey Auditory Verbal Learning Test
RMAP: Rush Memory an Aging Project
RxDx: Dementia Risk Index
SALSA: Sacramento Area Latino Study on Aging
SBP: Systolic blood pressure
SCORE2-OP: Systematic COronary Risk Evaluation (SCORE2)-Older Persons (≥ 70 years)
SNP: Single-nucleotide polymorphism
TBI: Traumatic brain injury
TE: Thromboembolism
THIN: The Health Improvement Network
TIA: Transient ischaemic attack
UK: United Kingdom
UKBDRS: UK Biobank Dementia Risk Score
USA: United States of America
VaD: Vascular dementia
WAISR: Wechsler Adult Intelligence Test Revised
WC: Waist circumference
WMS: Wechsler Memory Scale
yrs: Years
