# Supplementary material for: Discriminative performance of externally validated dementia risk prediction models: a systematic review and meta-analysis
Source: BMC Med. 2026 Feb 2;24:132. doi: 10.1186/s12916-026-04652-y (PMC12952151; doi:10.1186/s12916-026-04652-y)
Supplement: Supplementary file 1 — Additional file 1: Figure S1. PRISMA flow diagram for updated systematic reviews which include searches of databases. Figure S2. Forest plots describing the performanceof fully externally validated risk models for predicting all-cause dementia. Figure S3. Forest plots describing the performanceof fully externally validated risk models for predicting Alzheimer’s disease and vascular dementia. Figure S4. Publication bias. Table S1. Example of the electronic search strategy. Table S2. Risk of bias and applicability assessment using the Prediction model Risk Of Bias Assessment Tool . Table S3. Details of risk models that have been externally validated. Table S4. Dementia risk prediction model external validation results. Table S5. Comparison of the development and external validation study characteristics and methods for variable mapping. [file 12916_2026_4652_MOESM1_ESM.doc]

**Additional File**

**Title** Accuracy of Externally Validated Dementia Risk Prediction Models: An Umbrella Review of Systematic Reviews and Meta-Analysis

**Authors** Stephan, Brain et al

Table of Contents

[Acronyms 3](#__RefHeading___Toc213231726)

[Table S1 Example of the electronic search strategy (Ovid) 5](#__RefHeading___Toc213231727)

[Figure S1 PRISMA flow diagram for updated systematic reviews which include searches of databases 6](#__RefHeading___Toc213231728)

[Table S2 Risk of bias and applicability assessment using the Prediction model Risk Of Bias ASsessment Tool (PROBAST) [11] 7](#__RefHeading___Toc213231729)

[Table S3 Details of risk models that have been externally validated (alphabetical order by first author) 9](#__RefHeading___Toc213231730)

[Table S4 Dementia risk prediction model external validation results (alphabetical order by risk model) 16](#__RefHeading___Toc213231731)

[Figure S2 Forest-Plots describing the performance (i.e., c-statistic) of fully externally validated risk models for predicting all-cause dementia 43](#__RefHeading___Toc213231732)

[Figure S3 Forest-Plots describing the performance (i.e., c-statistic) of fully externally validated risk models for predicting Alzheimer’s disease and Vascular Dementia 49](#__RefHeading___Toc213231733)

[Figure S4 Publication Bias 51](#__RefHeading___Toc213231734)

[Table S5 Comparison of the development and external validation study characteristics and methods for variable mapping 58](#__RefHeading___Toc213231735)

# Acronyms

3MS = Modified Mini-Mental State Examination

ACT = Adult Changes in Thought

AD = Alzheimer’s disease

ADRD = Alzheimer’s Disease and Related Disorders score

AF = Atrial fibrillation

AFR = African American

aMCI = Amnestic mild cognitive impairment

ANU-ADRI = Australian National University- Alzheimer’s Disease Risk Index

APOE = Apolipoprotein e4 allele status

ASCVD = Atherosclerotic cardiovascular disease

BDSI = Brief dementia screening indicator

BMI = Body mass index

c-statistic = Concordance statistic

CAIDE = Cardiovascular risk factors, aging and dementia study

CARS = Cognitive ageing risk score

CHAP = Chicago Health and Aging Project

CHCS = Cardiovascular Health Cognition Study

CHD = Coronary heart disease

CHS = Cardiovascular Health Study

CSHA = Canadian Study of Health and Aging

CTDS = Cognitive Tests and Dependency Scale

CVD = Cardiovascular disease

DSM-III-R = Diagnostic and Statistical Manual (3^rd^ Edition, revised)

DSM-IV = Diagnostic and Statistical Manual (4^th^ Edition)

DSST = Digit Symbol Substitution

ESTHER Study = Epidemiologische Studie zu Chancen der Verhütung, Früherkennung und optimierten Therapie chronischer Erkrankungen in der älteren Bevölkerung

HER = Electronic health record

HK-MAPS = Hong Kong Memory and Ageing Prospective Study

FHS = Framingham Heart Study

FINDRISC = Finnish diabetes risk score

FRS = Framingham cardiovascular risk score

GRS-19 = Genetic risk score including 19 SNPs

H-EPESE = Hispanic Established Populations for the Epidemiologic Study of the Elderly

HDL=high-density lipoprotein

HLA = Hispanic Latino American

HRS = Health and Retirement Study

IADL = Instrumental Activities of Daily Living

ICD-9-CM = International Classification of Diseases, Ninth Revision, Clinical Modification

IGAP = International Genomics of Alzheimer’s Project

IST = Isaac Set Test

KPNC = Kaiser Permanente Northern California

LI = Lacuna infarcts

LIBRA = LIfestyle for BRAin Health score

LLDRI = Late-life dementia risk index

LR = Logistic regression
MAP = Memory and Aging Project

MADeN = Mexican-American Dementia Nomogram

MMSE = Mini Mental State Examination

MRI = Magnetic Resonance Imaging

MVL = Micro vascular lesions

N/A = Not applicable

NAVD = Non-AD or vascular dementia

NINDS-ADRDA = National Institute of Neurological and Communicative Disorders and Stroke and Alzheimer's Disease and Related Disorders Association

NSAID = Non-steroidal anti-inflammatory drugs

OLDW = OptumLabs Data Warehouse

PAQUID = Personnes Agees QUID

PRS = Polygenic Risk Score

PVD = Peripheral vascular disease

RADaR = Rapid Assessment of Dementia Risk

RAVLT = Rey Auditory Verbal Learning Test

RMAP = Rush Memory an Aging Project

RxDx = Dementia Risk Index

SALSA = Sacramento Area Latino Study on Aging

SBP = Systolic blood pressure

SCORE2-OP = Systematic COronary Risk Evaluation (SCORE2)-Older Persons (≥70 years)

SNP = Single-Nucleotide Polymorphism

TBI = Traumatic brain injury

TE = Thromboembolism

THIN = The Health Improvement Network

TIA = Transient ischemic attack

UK = United Kingdom

UKBDRS = UK Biobank Dementia Risk Score

USA = United States of America

VaD = Vascular dementia

WAISR = Wechsler Adult Intelligence Test Revised

WC = Waist circumference

WMS = Wechsler Memory Scale

yrs = Years

# Table S1 Example of the electronic search strategy (Ovid)

Database: Ovid MEDLINE(R)

Search Strategy:

--------------------------------------------------------------------------------

1 exp Dementia/

2 predict*.ti,ab,kf.

3 develop*.ti,ab,kf.

4 inciden*.ti,ab,kf.

5 sensitivity.ti,ab,kf.

6 specificity.ti,ab,kf.

7 exp "sensitivity and specificity"/

8 2 or 3 or 4 or 5 or 6 or 7

9 roc curve/

10 ROC.ti,ab,kf.

11 area under curve/

12 AUC.ti,ab,kf.

13 concordance statistic.ti,ab,kf.

14 c statistic.ti,ab,kf.

15 9 or 10 or 11 or 12 or 13 or 14

16 1 and 8 and 15

***************************

# Figure S1 PRISMA flow diagram for updated systematic reviews which include searches of databases

**Identification of new studies via databases**

**Previous studies**

**Identification of studies by other methods databases**

Records identified from databases (n = 3,786)

Records removed before screening; duplicate records removed (n = 791)

Studies included in previous versions of review i.e., met the inclusion criteria (n = 112)

Reports retrieved from citation chasing (n = 19)

**Identification**

Records screened

(n = 2,995)

Records excluded**

(n = 2,874)

Reports sought for retrieval

(n = 121)

Reports not retrieved

(n = 0)

Reports sought for retrieval

(n = 19)

Reports assessed for eligibility

(n = 1)

**Screening**

Reports assessed for eligibility

(n = 121)

Reports excluded (n = 105):

No external validation (n = 39)

Not population-based (n = 29)

Cross-sectional study (n = 11)

Outcome not dementia (n = 8)

Conference abstract (n = 8)

Review article (n = 3)

Original validation study not a risk prediction model (n=2)

Book chapter (n=2)

MCI sample (n = 1)

Protocol paper (n = 1)

Overlap in development & validation samples (n = 1)

New studies included in review

(n = 16)

External validation studies

(n = 19)

**Included**

Total studies included in review

(n = 36)

Total studies included in meta-analysis

(n = 17)

# Table S2 Risk of bias and applicability assessment using the Prediction model Risk Of Bias ASsessment Tool (PROBAST) [11]

| **Reference** | **ROB** | | | | **Applicability** | | | **Overall** | |
| --- | --- | --- | --- | --- | --- | --- | --- | --- | --- |
|  | **Participants** | **Predictors** | **Outcome** | **Analysis** | **Participants** | **Predictors** | **Outcome** | **ROB** | **Applicability** |
| Anatürk 2023 [33] | + | + | + | + | + | + | + | + | + |
| Anstey 2014 [14] | + | + | + | + | + | + | + | + | + |
| Ben-Hassen 2022 [16] | + | + | + | - | + | + | + | - | + |
| Capuano 2022 [17] | + | + | + | + | + | + | + | + | + |
| Casanova 2016 [18] | + | + | + | + | ? | + | + | + | ? |
| Chen 2023 [34] | + | + | + | + | - | + | + | + | - |
| Chouraki 2016 [19] | + | + | + | + | + | + | + | + | + |
| Coley 2023 [35] | + | + | + | ? | + | + | + | ? | + |
| Deckers 2020 [20] | + | + | ? | + | + | + | + | ? | + |
| Dhana 2024 [42] | + | + | + | + | + | + | + | + | + |
| Downer 2016 [68] | + | + | + | + | ? | + | ? | + | ? |
| Exalto 2014 [15] | + | + | + | + | ? | + | + | + | ? |
| Fayosse 2020 [22] | + | + | + | ? | ? | + | ? | ? | ? |
| Fisher 2021 [23] | + | + | + | + | + | + | + | + | + |
| Fung 2024 [43] | + | + | + | ? | + | + | + | ? | + |
| Hu 2022 [24] | + | + | + | + | + | + | + | + | + |
| Huque 2023 [9] | + | + | + | + | + | + | + | + | + |
| John 2022 [25] | + | ? | + | - | + | - | + | - | - |
| John 2024 [44] | + | + | + | + | + | + | + | + | + |
| Kivimäki 2023 [36] | + | + | + | - | + | + | + | - | + |
| Kootar 2023 [37] | + | ? | + | + | + | + | + | ? | + |
| Licher 2018 [27] | + | + | + | + | + | + | + | + | + |
| Licher 2019 [26] | + | + | + | + | + | + | + | + | + |
| Reeves 2024 [45] | + | + | + | + | + | + | + | + | + |
| Schiepers 2018 [28] | + | + | + | - | + | ? | + | - | ? |
| Shang 2022 [38] | + | + | + | + | + | + | + | + | + |
| Stephan 2020 [32] | + | + | + | - | - | + | + | - | - |
| Stephan 2023 [39] | + | + | + | + | + | + | + | + | + |
| Trares 2024 [10] | + | + | + | + | + | + | + | + | + |
| Vonk 2021 [29] | + | + | + | + | ? | + | + | + | ? |
| Vos 2017 [30] | + | + | + | + | + | + | + | + | + |
| Walters 2016 [31] | + | + | ? | + | + | + | + | ? | + |
| Yang 2022 [40] | + | + | + | + | - | ? | + | + | - |
| Yang 2024 [47] | + | + | + | + | ? | + | + | + | ? |
| Wang 2024 [46] | + | + | + | + | + | + | + | + | + |
| Zheng 2023 [41] | + | + | + | + | ? | + | + | + | ? |

PROBAST = Prediction model Risk Of Bias ASsessment Tool; ROB = risk of bias.* + indicates low ROB/low concern regarding applicability; − indicates high ROB/high concern regarding applicability; and ? indicates unclear ROB/unclear concern regarding applicability.

# Table S3 Details of risk models that have been externally validated (alphabetical order by first author)

| **Reference** | **Model** | **Development sample** | **Country (Representative Y/N)** | **Sample size** | **Baseline age** | **Follow-up (years)** | **Number of variables: Predictor variables (model)** | **Method used to develop model** | **Outcome** | **Incident Dementia case number or %** | **c-statistic (95%CI)** | **Calibration results** |
| --- | --- | --- | --- | --- | --- | --- | --- | --- | --- | --- | --- | --- |
| Anstey (2013) [59] | ANU-ADRI | N/A | N/A | N/A | N/A | N/A | 15: Age, sex, education, obesity (BMI), diabetes, depression, cholesterol, TBI, smoking, alcohol, social engagement, physical activity, cognitive activity, fish intake & pesticide exposure | Evidence synthesis | AD | N/A | N/A | N/A |
| Anstey (2022) [62] | CogDrisk | N/A | N/A | N/A | N/A | N/A | 17: Age, sex, education, hypertension, midlife obesity, high cholesterol (<60 years), diabetes, insufficient physical activity, depression, TBI, atrial fibrillation, smoking, social engagement, cognitive engagement, fish consumption (diet), stroke & insomnia | Evidence synthesis | Any dementia | N/A | N/A | N/A |
| Anstey (2022) [62] | CogDrisk-AD | N/A | N/A | N/A | N/A | N/A | 16: Age, sex, education, hypertension, midlife obesity, high cholesterol (<60 years), diabetes, insufficient physical activity, depression, TBI, smoking, social engagement, cognitive engagement, fish consumption (diet), stroke & pesticide exposure | Evidence synthesis | AD | N/A | N/A | N/A |
| Anaturk (2023) [33] | UKBDRS | UKB | UK (No) | 176,611 | 50-73 | 14 | 11: Age, sex, education, parental history of dementia, material deprivation, diabetes, stroke, depression, hypertension, high cholesterol & household occupancy | Cox and Fine-Gray | All-cause (ICD-9 and ICD-10) | Not reported | 0.79 (0.78 to 0.79) | Not reported |
| Anaturk (2023) [33] | UKBDRS+APOE | UKB | UK (No) | 157,090 | 50-73 | 14 | 12: Age, sex, education, parental history of dementia, material deprivation, diabetes, stroke, depression, hypertension, high cholesterol, household occupancy & APOE | Cox and Fine-Gray | All-cause (ICD-9 and ICD-10) | Not reported | 0.81 (0.81–0.81) | Not reported |
| Barnes (2009) [72] | LLDRI | CHCS | USA (No) | 3,375 | 65-100 | 6 | 11: Age (75-79; 80-100), low 3MS, low DSST, underweight (BMI), APOE, MRI white matter disease, MRI enlarged ventricles, internal carotid artery thickness on ultrasound, coronary bypass surgery, slow physical performance (time to put on and button shirt >45 seconds) & lack of alcohol consumption | LR | Consensus diagnosis of all-cause and subtypes e.g., AD (NINCDS-ADRD), VaD (State of California Alzheimer’s  Disease Diagnostic and Treatment Centres criteria), mixed or other | 14% | 0.81 (0.79–0.83) | Good |
| Barnes (2014) [67] | BDSI | Combined data from: CHS, FHS, HRS, and SALSA | USA (No) | Range: 1,125 to 13,889 | ≥65 | 6 | 7: Age, education (<12 years), underweight (BMI), diabetes, stroke, IADL (needs help with money or medications) & composite depressive symptom variable (antidepressant medication use or depressive symptoms i.e., reporting that “everything was an effort” for 3 or more days/week during the past week) | Cox | All-cause (study-specific criteria, DSM-IV, or a cut-off on a brief cognitive battery) | Range 7.1-15.6% | CHS: 0.68 (0.65–0.72)  FHS: 0.77 (0.73–0.82)  HRS: 0.76 (0.74–0.77)  SALSA: 0.78 (0.72–0.83) | Good |
| Barnes (2020) [53] | eRADAR (Full model) | Kaiser Permanente Washington (ACT Study) | USA (No) | 4,330 | ≥65 | Biennial | 30: Age, sex, congestive heart failure, cerebrovascular disease, diabetes (complex), chronic pulmonary disease, hypothyroidism, renal failure, solid tumour without metastases, rheumatoid arthritis, weight loss, fluid and electrocyte disorders, blood loss anaemia, psychoses, TBI, tobacco use disorder, AF, gait abnormality, underweight, obese, high blood pressure, ≥1 outpatient visit, ≥ emergency department visit, ≥1 physical therapy visit, ≥1 speech, language and learning visit, fill for non-tricyclic antidepressant, ≥1 clinic “no show”, ≥1 ACS hospitalizations, ≥1 ACS emergency visits & medication non-adherence (hypoglycemic, antihypertensive or statin medications) | LR | Dementia (EHR diagnosis codes and medication fills) | 23.4% | 0.79 (0.77-0.81) | Reasonable |
| Ben-Hassen (2021) [16] | CTDS | PAQUID | France (No) | 2,880 | ≥65 | Mean 9.3 | 3: IST, MMSE & IADL (using the phone, using means of transport, taking medication as prescribed & managing money) | Joint models | All-cause (DSM-III-R) | 28.2% | Range 0.93-0.96 (over 10-year prediction frame) | Well calibrated at 5-years |
| Capuano (2022) [17] | RADaR | RMAP | USA (No) | 1,780 | ≥65 | Median 9 | 6: Age, memory complaints, difficulty with finances, recall of the month, recall of the room & 3-word delayed recall | Fine-Gray | All-cause (NINDS-ADRDA) | Not reported | 0.81 (0.78-0.85) | Not reported |
| Chen (2023) [34] | CLHLS Risk Model-Illiterate Women | CLHLS (2011-2014 cohort) | China (Yes) | 1,864 (1,304 training and 560 internal validation) | ≥60 | Median 2.8 (IQR 2.3-2.9) | 7: Age, MMSE, waist-to-height ratio, psychological score, ADL, IADL and frequency of tooth brushing | Machine learning (Random survival forest) and Cox | Chinese MMSE≤17 | n=460 (24.7%) | 0.77 (training) and 0.80 (internal validation) | Not reported |
| Chouraki (2016) [19] | Demographic + APOE | N/A | N/A | N/A | N/A | N/A | 4: Age, sex, education & APOE status | Proposed in study with no specific modelling | N/A | N/A | N/A | N/A |
| Chouraki (2016) [19] | GRS-19 | N/A | N/A | N/A | N/A | N/A | 19: 19 SNPs^a^ (controlling for age, sex, education & APOE e4 status) | Meta-analysis from IGAP | N/A | N/A | N/A | N/A |
| Conroy (2003) [48] | SCORE | SCORE project | Multi-countries in Europe (No) | 205, 178 | 45–64 | 10 | 4: Age, sex, SBP & total cholesterol | Weibull model | Not developed for predicting dementia but fatal CVD | N/A | 0.71 to 0.84 | Not reported |
| D’Agostino (2008) [49] | FRS | Framingham study | USA (No) | 8,491 | 30-74 | 12 | 7: Age, smoker, diabetic, SBP, BP treatment, HDL & total cholesterol [Scores stratified by sex] | Cox | Atherosclerotic CVD events: CHD, cerebrovascular disease, PVD, and heart failure | N/A | **Male** 0.76  **Female** 0.79 | Excellent (both sexes) |
| Deckers (2015) [61] | LIBRA | N/A | N/A | N/A | N/A | N/A | 12^b^: Low/moderate alcohol consumption, coronary heart disease, physical inactivity, renal dysfunction, diabetes, cholesterol, smoking, obesity, hypertension, depression, cognitive activity (high) & Mediterranean diet | Evidence Synthesis and Delphi consensus | N/A | N/A | N/A | N/A |
| Downer (2016) [21] | MADeN | H-EPESE | USA (No) | 1,739 | ≥65 | 10 | 10: Age, sex, education, not having friends to count on, not attending community events, diabetes, feeling the blues, pain, impairment in IADL & unable to walk a half mile | Fine-Gray | All-cause (AA-NIA) | n=229 | 0.74 (0.70-0.78) | Well calibrated |
| Fisher (2021) [23] | Dementia Population Risk Tool (DemPoRT) | Canadian Community Health Survey (CCHS) Ontario respondents (2001, 2003, 2005 and 2007/2008) | Canada (No) | 47,739 | ≥55 | 5 | 28 [Full model, by sex]: Age, education, ethnicity, marital status, neighborhood deprivation, immigrant status, multilingualism, stress, sense of belonging, self-rated health, smoking status, packet years of smoking, former drinker, drinks last week, daily fruit and vegetable consumption, daily potato consumption, daily juice consumption, leisure physical activity, function (combined score including personal hygiene and care, locomotion in home, meal preparation, running errands, ordinary housework, finances), heart disease, stroke, diabetes, mood disorder, hypertension, COPD, epilepsy, BMI &survey year + interaction terms (n=24)  **NOTE** Reduced model was tested in the combined development and validation samples and is not reported here | Fine-Gray | All-cause (Health records) | n=6,734 | **Male** 0.82 (0.80-0.83)  **Female** 0.82 (0.81-0.83) | Well calibrated |
| Fung (2024) [43] | CARS | Community dwelling older adults (No specific name reported) | Hong Kong (No) | 289 | ≥60 | 6 | 9: Age, sex, education, poorly controlled diabetes, prolonged sleep latency, mind body exercise, light exercise, loneliness & APOE | LR | All-cause (Cognitive z-score criteria; Scores of the CMMSE, ADAS-cog, 10-minute delayed recall, digit span, and CVFT of the CDR 0.5 participants were expressed as z-scores with reference to the mean and SD of CDR) | n=72 (24.9%) | 0.83 (0.80-0.90) | Not reported |
| Hogan (2000) [65] | CSHA Derived Score | CSHA | Canada (Yes) | 892 | ≥65 | 5 | 3: Age, 3MS & informant’s report if the presence of memory problems | LR | All-cause (DSM-III-R) and AD (NINCDS-ADRDA) | 26.9% | **All-cause** 0.78  **AD** 0.81 | Not reported |
| Hu (2022) [24] | CLHLS Risk Model | Chinese Longitudinal Healthy Longevity Survey (CLHLS) 2011-2018 cohort | China (Yes) | 10,053 | ≥65 | Median 5.7 | 8: Age, sex, education, marital status, activity duration, leisure activity (i.e., playing cards or mah-jongg, watching TV or listening to the radio), stroke & cardiovascular disease | Cox and Fine-Gray | Chinese MMSE≤17 | 17.4% | **Cox** 0.71 (0.70-0.72)  **Fine-Gray** 0.71 (0.70-0.73) | Not reported |
| Jessen (2011) [56] | AgeCoDe | Ageing, Cognition and Dementia Study | Germany (No) | 3,055 | ≥75 | Mean 3.8 (Max 6.1) | 6: Age, subjective memory impairment, verbal fluency, delayed recall, MMSE & IADL impairment | Cox | AD (DSM-IV, NINCDS  -ADRDA) | 6.3% | 0·84 (0·80–0·88) | Not reported |
| John (2024) [44] | OPERH Phenotype Model | OPEHR | USA (Yes) | 971,999 | 55-84 | 5 | NOTE: Unique phenotype models were developed across the five datasets and two analysis (regularization) methods. The model reported here is the final (best) phenotype model reported by in the publication.  19: Age, sex, history of acetaminophen exposures, antiepileptic medication exposures, seizures, anemia, cancer, major depressive disorder, chronic obstructive pulmonary disease, heart failure, chronic kidney disease, hyperlipidemia, hypertension, obesity, alcoholism, anxiety, Type 2 diabetes, smoking, & urinary tract infections | LR (with two types of regularization: LASSO & Broken Adaptive Ridge) | All-cause (Concept codes from OMOP CDM) | n=37,978 (3.9%) | 0.81 | E_ave_=0.0018 |
| Kivipelto (2006) [61] | CAIDE | Cardiovascular Risk Factors,  Aging, and Dementia Study | Finland (No) | 1,409 | 39-64 | Mean 20.0 | 7: Age, sex, education, SBP, BMI, total cholesterol & physical activity | LR | Dementia (DSM-IV) | 4% | 0.77 (0.71–0.83) | Not reported |
| Kivipelto (2006) [55] | CAIDE+APOE | Cardiovascular Risk Factors,  Aging, and Dementia Study | Finland (No) | 1,409 | 39-64 | Mean 20.0 | 8: Age, sex, education, SBP, BMI, total cholesterol, physical activity & APOE | LR | Dementia (DSM-IV) | 4% | 0.78 (0.72-0.84) | Not reported |
| Lindström (2003) [52] | FINDRISC | The National FINRISK Study | Finland (unclear) | 4,746 | 35-64 | 10 | 8: Age, BMI, WC, physical activity, food (daily consumption of vegetables, fruit, or berries), blood pressure medication, history of high blood glucose & family history of diabetes | LR | Not developed for predicting dementia but individuals at high risk of type 2 diabetes | N/A | 0.85 to 0.87 | Not reported |
| Li (2018) [66] | FDRS | FHS | USA (No) | 2,383 | ≥60 | 5, 10 and 20 | 7: Age, marital status, BMI, stroke, diabetes, ischemic attacks & cancer | Cox | Dementia (Consensus criteria) | 32.7% | 0.72 (all three time points) | Good |
| Licher (2019) [26] | Basic Dementia Risk Model (Basic-DRM) | Rotterdam Study | Netherlands (No) | 2,710 | 60–96 | Median 7 | 4: Age, stroke, subjective memory decline & IADL (needs help with finances or medication) | Fine-Gray | Dementia and AD (DSM-III-R and NINCDS-ADRD) | 4.8% | 0.78 (0.75–0.81) | Not reported |
| Licher (2019) [27] | Basic-DRM (Extended) | Rotterdam Study | Netherlands (No) | 2,710 | 60–96 | Median 7 | 12: Age, age-squared, sex, education, SBP, current smoking, depressive symptoms, diabetes, symptomatic stroke, parental history of dementia, subjective memory decline & need for assistance with medication and finances | Fine-Gray | Dementia and AD (DSM-III-R and NINCDS-ADRD) | 4.8% | 0.79 (0.76-0.83) | Not reported |
| Mapstone (2014) [60] | Plasma phospholipids (10-metabolite panel) | Rochester/Orange County Aging Study | USA (No) | 101 | ≥70 | 2-3 | 10: 10-metabolite panel in plasma including Propionyl AC (C3), LysoPC a C18:2, PC aa C36:6, C16:1-OH, PC aa C38:0, PC aa C38:6, PC aa C40:1, PC aa C40:2, PC aa C40:6 & PC ae C40:6 | LR | aMCI/AD combined | n=28 | 0.92 (0.87-0.98) | Not reported |
| Mehta (2016) [54] | RxDx-DRI (Dementia Risk Index) | CPRD (2003-2012); patients with hypertension and diabetes | UK (Yes) | 133,176 | ≥60 | Not clear | 26: Age, sex, myocardial infarction, congestive heart failure, coronary and peripheral vascular disease, cerebrovascular disease, chronic pulmonary disease, rheumatologic disease, peptic ulcer disease, renal disease/end stage renal disease, mild liver disease/moderate or severe liver disease, any malignancy (including lymphoma, leukaemia, and metastatic solid tumour), epilepsy, hyperlipidaemia, Parkinson’s disease,  cardiac disease ASCVD, glaucoma, transplantation, thyroid disorder, gout, Crohn’s and ulcerative disease, pain and inflammation/pain, depression, psychotic illness, bipolar disorders & anxiety and tension | Cox | All-cause | 3.4% | 0.81 (0.80–0.81) | Poor |
| Nori (2019) [63] | Nori-ADRD Score | OLDW | USA (No) | 215,196 | ≥45 | 3-8 years | 50^c^: Demographic measures, medical diagnosis, prescription medication & medical procedures | Machine Learning (development) and LR (final model) | ADRD & MCI combined | n=35,866 | 0.64 (0.64-0.65) | Not reported |
| Reeves (2024) [45] | DemRisk-young | CPRD GOLD (2005-17) – 235 GP practices selected | UK (Yes) | 616,366 | 60-79 | 5 years (Median 2.7) | 22: Age, age-squared, deprivation, depression, stroke, diabetes, epilepsy, gait problems, major head injury, selective serotonin reuptake inhibitor use, tricyclic antidepressant use, other antidepressant use, mood stabilisers, NSAIDs (excluding aspirin), anticholinergic burden over last year (square-root), ever received social services, number of A&E visits in last year (square root), number of GP consultations involving third party in last year (square root), number of GP home visits in last year (square root), number of missed GP appointments in last year (square root), most recent BMI (square root), and most recent mean systolic blood pressure value/20 | Cox | All-cause (Primary Care record or ICD-10) | n=10,841 | 0.79 (0.78-0.79) | Not reported |
| Reeves (2024) [45] | DemRisk-old | CPRD GOLD (2005-17) – 235 GP practices selected | UK (Yes) | 175,131 | 80-89 | 5 years (Median 1.8) | 20: Sex, age-80, age-80 squared, deprivation, stroke, diabetes, gait problems, major head injury, selective serotonin reuptake inhibitor use, tricyclic antidepressant use, other antidepressant use, NSAIDs (excluding aspirin), anticholinergic burden over last year (square-root), ever received social services, number of A&E visits in last year (square root), number of GP consultations involving third party in last year (square root), number of GP home visits in last year (square root), number of missed GP appointments in last year (square root), most recent BMI (square root), and most recent mean systolic blood pressure value/20 | Cox | All-cause (Primary Care record or ICD-10) | n=15,994 | 0.65 (0.64-0.64) | Not reported |
| Schiepers (2018) [28] | LIBRA-Modified | Maastricht Ageing Study (MAAS) | Netherlands (No) | 949 | ≥50 | 12 years | 15: Age, sex, education, low/moderate alcohol consumption, coronary heart disease, physical inactivity, renal dysfunction, diabetes, cholesterol, smoking, obesity, hypertension, depression, cognitive activity (high) & Mediterranean diet | Cox | All-cause (DSM-IV) | 6.4% (n=61) | 0.75 (0.69-0.80)  NOTE: 0.59 (0.52-0.66) excluding age and sex | Not reported |
| SCORE2 (2021) [50] | SCORE2 | Emerging Risk Factor Collaboration (ERFC) and  the UKB | Multi-country (No) | 677,684 | 40-69 | 10 years | 7: Age (40-69 years), sex, smoking, SBP, diabetes, total cholesterol & HDL cholesterol + interactions between risk factors & age | Fine-Gray | Not originally developed for dementia but cardiovascular disease | N/A | 0.74 (0.74–0.74) | Reasonable |
| SCORE2-OP (2021) [51] | SCORE2-OP | Cohort of Norway (CONOR)  study | Norway (Yes) | 28,503 | ≥65 | 10 years | 7: Age (≥70 years), sex, smoking, SBP, type 2 diabetes mellitus, total cholesterol & HDL cholesterol + interaction terms between risk factors & age | Fine-Gray | Not originally developed for dementia but cardiovascular disease | N/A | 0.66 (0.65–0.66) | Good |
| Stephan (2023) [39] | HAAS (NIA-Reagan) | HAAS | USA (No) | 2,960 (non-autopsied; all male) | 45-68 | 30 (Mean 27.8; SD 2.9) | 4: Age, hypertension treatment, smoking & APOE | LR | All-cause (DSM-III-R), AD (NINCDS-ADRDA), VaD (State of California AD and Diagnostic Treatment Centres criteria) | 11.5% | **All-cause** 0.62 (0.59-0.65)  **AD** 0.64 (0.59-0.68)  **VaD** 0.58 (0.53-0.63) | **All-cause** Poor  **AD** Poor  **VaD** Poor |
| Stephan (2023) [39] | HAAS (NP) | HAAS | USA (No) | 2,960 (non-autopsied; all male) | 45-68 | 30 (Mean 27.8; SD 2.9) | 3: Age, smoking & APOE | LR | As above | 11.5% | **All-cause** 0.62 (0.59-0.65)  **AD** 0.65 (0.61-0.69)  **VaD** 0.55 (0.50-0.61) | **All-cause** Poor  **AD** Poor  **VaD** Poor |
| Stephan (2023) [41] | HAAS (NFT) | HAAS | USA (No) | 2,960 (non-autopsied; all male) | 45-68 | 30 (Mean 27.8; SD 2.9) | 4: Age, stroke, alcohol use & APOE | LR | As above | 11.5% | **All-cause** 0.65 (0.62-0.68)  **AD** 0.64 (0.60-0.69)  **VaD** 0.66 (0.61-0.71) | **All-cause** Poor  **AD** Poor  **VaD** Poor |
| Stephan (2023) [39] | HAAS (MVL) | HAAS | USA (No) | 2,960 (non-autopsied; all male) | 45-68 | 30 (Mean 27.8; SD 2.9) | 5: Age, education, SBP, stroke & APOE | LR | As above | 11.5% | **All-cause** 0.58 (0.54-0.61)  **AD** 0.49 (0.44-0.54)  **VaD** 0.71 (0.64-0.77) | **All-cause** Poor  **AD** Poor  **VaD** Poor |
| Stephan (2023) [39] | HAAS (LI) | HAAS | USA (No) | 2,960 (non-autopsied; all male) | 45-68 | 30 (Mean 27.8; SD 2.9) | 4: Age, cholesterol treatment, SBP & diabetes | LR | As above | 11.5% | **All-cause** 0.62 (0.59-0.65)  **AD** 0.57 (0.53-0.62)  **VaD** 0.68 (0.62-0.73) | **All-cause** Poor  **AD** Poor  **VaD** Good |
| Stephan (2023) [39] | HAAS (MIXED) | HAAS | USA (No) | 2,960 (non-autopsied; all male) | 45-68 | 30 (Mean 27.8; SD 2.9) | 3: Age, SBP & APOE | LR | As above | 11.5% | **All-cause** 0.61 (0.58-0.64)  **AD** 0.60 (0.56-0.64)  **VaD** 0.58 (0.53-0.64) | **All-cause** Poor  **AD** Poor  **VaD** Poor |
| Tierney (2005) [70] | Tierney (2005) | CSHA | Canada (Yes) | 263 | ≥65 | 10 | 3: Age, education & RAVLT short delayed verbal recall score | LR | AD (DSM-IV and NINCDS-ADRDA) | n=47 | 0.77 (R^2^=0.22) | Not reported |
| Tierney (2010) [71] | Tierney (2010) | CSHA | Canada (Yes) | 284 | ≥65 | 10 | 5: Age, sex, education, RAVLT short delayed verbal recall score & WAISR Digit Symbol | LR | Dementia (DSM-III-R and DSN-IV) | n=75 | 0.79 (R^2^=30%) | Not reported |
| Verhaaren (2013) [72] | Verhaaren-2 variable | Rotterdam Study | Netherlands (No) | 5,171 | 45-99 | 10 | 2: Age & sex | LR | AD (DSM-III-R and National Institute of Neurological and Communicative  Diseases and Stroke/Alzheimer’s Disease and Related Disorders Association) | n=359 | 0.79 (0.77-0.81) | Not reported |
| Verhaaren (2013) [72] | Verhaaren-3 variable | Rotterdam Study | Netherlands (No) | 5,171 | 45-99 | 10 | 3: Age, sex & APOE | LR | AD (DSM-III-R and National Institute of Neurological and Communicative  Diseases and Stroke/Alzheimer’s Disease and Related Disorders Association) | n=359 | 0.81 (0.80-0.83) | Not reported |
| Verhaaren (2013) [72] | Verhaaren-4 variable | Rotterdam Study | Netherlands (No) | 5,507 | 45-99 | 10 | 4: Age, sex, APOE & Genetic Risk Score (full genetic risk score including CLU, PICALM, BIN1, CR1, ABCA7, MS4A6A, MS4A4E, CD2AP, EPHA1, CD33) | LR | AD (DSM-III-R and National Institute of Neurological and Communicative  Diseases and Stroke/Alzheimer’s Disease and Related Disorders Association) | n=359 | 0.82 (0.80-0.83) | Not reported |
| Walters (2016) [30] | Dementia Risk Score (DRS)-young | THIN | UK (No) | 800,013 | 60-79 | 5 | 15: Age, age-squared, sex, calendar year, local area deprivation, BMI, BMI-squared, current anti-hypertensive use, smoking status, alcohol misuse, depression/anti-depressant use, aspirin use, history of diabetes, history of stroke or TIA & history of AF | Cox | All-cause including AD, VaD, and unspecified or mixed dementia (ICD-10) | n=6,017 | Not reported | Not reported |
| Walters (2016) [31] | Dementia Risk Score (DRS)-old | THIN | UK (No) | 130,382 | 80-95 | 5 | 17: Age, age-squared, sex, calendar year, BMI, current anti-hypertensive use, SBP, lipid ratio, smoking status, hazardous/harmful alcohol drinking, current depression/anti-depressants, current anxiety/anxiolytics, current aspirin use, current NSAID use (not aspirin), diabetes, stroke or TIA & AF | Cox | All cause including AD, vascular dementia, and unspecified or mixed dementia (ICD-10) | n=1,483 | Not reported | Not reported |
| Yang (2022) [40] | Model A | MAP | USA (No) | 1,179 | Not reported | 3 & 5 | 55: Non-cognitive covariates | Cox | AD | Not reported | Not reported | Not reported |
| Yang (2022) [40] | Model B | MAP | USA (No) | 1,179 | Not reported | 3 & 5 | 56: 55 non-cognitive covariates + MMSE | Cox | AD | Not reported | Not reported | Not reported |
| Yang (2022) [40] | Model C | MAP | USA (No) | 1,179 | Not reported | 3 & 5 | 57: 55 non-cognitive covariates+ MMSE+ Composite cognition | Cox | AD | Not reported | Not reported | Not reported |
| Yang (2022) [40] | Model D | MAP | USA (No) | 1,179 | Not reported | 3 & 5 | 1: Composite cognition score | Cox | AD | Not reported | Not reported | Not reported |

**Notes**

**- Green boxes indicate models that were not developed for predicting an outcome of dementia.**

a. 19 SNPS include: rs6656401 (CR1), rs6733839 (BIN1), rs35349669 (INPP5D), rs190982 (MEF2C), rs9271192 (HLA-DRB5/ HLA-DRB1), rs10948363 (CD2AP), rs2718 058 (NME8), rs1476679 (ZCWPW1), rs11771145 (EPHA1), rs28834970 (PTK2B), rs9331896 (CLU), rs10838725 (CELF1), rs983392 (MS4A6A), rs10792 832 (PICALM), rs11218343 (SORL1), rs17125944 (FERMT2), rs10498633 (SLC24A4/RIN3), rs4147 929 (ABCA7), and rs7274581 (CASS4).Final model was adjusted for age, sex, education and APOE e4 status

b. Some risk factors were identified that need further validation (few studies, inconsistent results) including coronary heart disease, renal dysfunction, diet, and cognitive activity

c. List of n=50 variables includes: (1) memory loss; (2) paralysis agitans;, (3) mild cognitive impairment, so stated; (4) bipolar disorder, unspecified; (5) unspecified psychosis; (6) loss of weight; (7) depressive disorder, not elsewhere classified; (8) altered mental status; (9) personal history of fall; (10) other convulsions; (11) unspecified fall; (12) other chronic pain; (13) acute, but ill-defined, cerebrovascular disease; (14) urge incontinence; (15) other alteration of consciousness; (16) unspecified constipation; (17) unspecified urinary incontinence; (18) encounter for long-term (current) use of other medications; (19) lack of coordination; (20) other malaise and fatigue; (21) diabetes mellitus; (22) abnormality of gait; (23) dizziness and giddiness; (24) unspecified cerebral artery occlusion with cerebral infarction; (25) diabetes mellitus; (26) edema; (27) muscle weakness (generalized); (28) urinary tract infection, site not specified; (29) cpt screening mammography; computer-aided detection; (30) computed tomography, head or brain; (31) radiologic examination, chest; single view, frontal; (32) venlafaxine hcl; (33) duloxetine hcl; (34) tolterodine tartrate; (35) sertraline; (36) citalopram hydrobromide; (37) potassium chloride; (38) oxybutynin chloride; (39) hydrocodone bit/acetaminophen; (40) propoxyphene/acetaminophen; (41) sulfamethoxazole/trimethoprim; (42) metformin; (43) blood sugar diagnostic; (44) lisinopril; (45) cephalexin monohydrate; (46) simvastatin; (47) clopidogrel bisulfate; (48) tramadol; (49) gabapentin; and , (50) furosemide.

# Table S4 Dementia risk prediction model external validation results (alphabetical order by risk model)

| **Included in Meta-Analysis** | **Author (year)** | **Validation Data Source** | **Country (Nationally Representative)** | **Sample size** | **Baseline age** | **Follow-up (years)** | **Full or partial validation** | **Predictor variables** | **Missed variables** | **Outcome** | **Incident Dementia, %** | **Statistical Model** | **c-statistic (95%CI)** | **Calibration Result** |
| --- | --- | --- | --- | --- | --- | --- | --- | --- | --- | --- | --- | --- | --- | --- |
|  | **Model Validated** AgeCoDe |  |  |  |  |  |  |  |  |  |  |  |  |  |
| Y | Stephan (2020) [32] | 10/66 Study | China (No) | 637 | ≥65 | 3-5 | Full (6) | Age, subjective memory impairment, verbal fluency, delayed recall, MMSE & IADL impairment | None | All-cause (10/66 Algorithm) | 19.9% | Cox | 0.57 (0.52-0.62) | Good |
| Y | Stephan (2020) [32] | 10/66 Study | Cuba (No) | 1,007 | As above | As above | Full (6) | As above | As above | As above | 11.4% | As above | 0.68 (0.63-0.73) | As above |
| Y | Stephan (2020) [32] | 10/66 Study | Dominican Republic (No) | 634 | As above | As above | Full (6) | As above | As above | As above | 16.6% | As above | 0.61 (0.55-0.66) | As above |
| Y | Stephan (2020) [32] | 10/66 Study | Mexico (No) | 610 | As above | As above | Full (6) | As above | As above | As above | 13.9% | As above | 0.64 (0.59-0.70) | As above |
| Y | Stephan (2020) [32] | 10/66 Study | Peru (No) | 563 | As above | As above | Full (6) | As above | As above | As above | 11.2% | As above | 0.69 (0.63-0.76) | As above |
| Y | Stephan (2020) [32] | 10/66 Study | Puerto Rico (No) | 724 | As above | As above | Full (6) | As above | As above | As above | 16.6% | As above | 0.71 (0.66-0.75) | As above |
| Y | Stephan (2020) [32] | 10/66 Study | Venezuela (No) | 394 | As above | As above | Full (6) | As above | As above | As above | 21.0% | As above | 0.74 (0.69-0.79) | As above |
|  | **Model Validated**  ANU-ADRI |  |  |  |  |  |  |  |  |  |  |  |  |  |
| N | Anstey (2014) [14] | CVHS | USA (No) | 2,496 | ≥62 | Median 6.0 | Partial (9 out of 15) | Age, sex, education, diabetes, smoking, alcohol, PA, fish intake & depression symptoms | BMI, cholesterol, pesticide exposure, TBI, cognitive activity & social engagement | All-cause (study-specific criteria) & AD (NINCDS-ADRDA) | 11.1% (AD) | Cox | **All-cause**  0.73 (0.70-0.75)  **AD**  0.74 (0.71-0.77) | NR |
| N | Anstey (2014) [14] | MAP | USA (No) | 903 | ≥54 | Mean 3.5 | Partial (10 out of 15) | Age, sex, education, diabetes, TBI, cognitive activity, social engagement, smoking, alcohol & PA | BMI, cholesterol, pesticide exposure, fish intake & depressive symptoms | All-cause & AD (NINCDS-ADRA) | 17·5% (AD) | Cox | **All-cause**  0.72 (0.68-0.76)  **AD**  0.73 (0.69-0.78) | NR |
| N | Anstey (2014) [14] | KP | Sweden (No) | 905 | ≥74 | Mean 6.0 | Partial (8 out of 15) | Age, sex, education, diabetes, TBI, social engagement, smoking & alcohol | BMI, cholesterol, pesticide exposure, cognitive activity, PA, fish intake & depressive symptoms | All-cause & AD (DSM-III-R) | 20·0% (AD) | Cox | **All-cause**  0.65 (0.62-0.69)  **AD**  0.64 (0.60-0.68) | NR |
| N | Anstey (2014) [14] | CVHS | USA (No) | 2496 | ≥62 | Median 6.0 | Partial (6 out of 15) | Age, sex, education, diabetes, smoking & alcohol | BMI, cholesterol, pesticide exposure, TBI, social engagement, cognitive activity, PA, fish intake & depressive symptoms | All-cause (study-specific criteria) & AD (NINCDS-ADRDA) | 11.1% (AD) | Cox | **All-cause**  0.72 (0.70-0.75)  **AD**  0.73 (0.71–0.76) | NR |
| N | Anstey (2014) [14] | MAP | USA (No) | 903 | ≥54 | Mean 3.5 | As above | As above | As above | All-cause & AD (NINCDS-ADRA) | 17.5% (AD) | Cox | **All-cause**  0.68 (0.64-0.72)  **AD**  0.69 (0.65-0.73) | NR |
| N | Anstey (2014) [14] | KP | Sweden (No) | 905 | ≥74 | Mean 6.0 | As above | As above | As above | All-cause & AD (DSM-III-R) | 20.0% (AD) | Cox | **All-cause**  0.68 (0.64–0.71)  **AD**  0.67 (0.63-0.70) | NR |
| N | Anaturk (2023) [33] | UKB – Training dataset | UK (No) | 176,611 | ≥50 | 14 | Partial (14 out of 15) | Age, sex, education, BMI, diabetes,  TBI, depressive symptoms, cholesterol, social engagement,  smoking, alcohol, PA, fish intake & pesticide exposure | Cognitive activity levels | All-cause (ICD-9, ICD-10, self-reported, primary/secondary cause of death or use of Donepezil,  Rivastigmine, Galantamine,  Modafinil or Memantine) | 1.7% (n=3,813) [total sample] | Fine Gray | 0.57 (0.57-0.57) | NR |
| N | Anaturk (2023) [33] | UKB – Testing dataset | UK (No) | 44,151 | ≥50 | 14 | As above | As above | As above | As above | As above | As above | 0.57 (0.54-0.59) | NR |
| N | Anaturk (2023) [33] | UKB – Testing dataset | UK (No) | 44,151 | As above | 1-5 | As above | As above | As above | As above | n=99 | As above | 0.54 (0.48- 0.59) | NR |
| N | Anaturk (2023) [33] | UKB – Testing dataset | UK (No) | 44,151 | As above | 5-10 | As above | As above | As above | As above | n=401 | As above | 0.58 (0.55-0.60) | NR |
| N | Anaturk (2023) [33] | Whitehall II | UK (No) | 2,934 | Median 57 (IQR 10) | 14 | Partial (13 out of 15) | Age, sex, education, BMI, diabetes,  depressive symptoms, cholesterol, cognitive activity levels, social engagement, smoking, alcohol, PA & fish intake | TBI & pesticide exposure | All-cause (self-report & hospital in-patient records: ICD-9 & ICD-10) | 3.2% (n=93) | As above | 0.52 (0.45-0.58) | Not undertaken due to insufficient data |
| N | Dhana (2024) [42] | CHAP | USA (No) | 2,130 (1,159 Black or African American and 971 White) | ≥65 | 5, 6, 10, 15 | Partial | Age, education, diabetes, symptoms of depression, TBI, smoking, alcohol intake, social engagement, PA, cognitive activity, and fish intake | Cholesterol, BMI, pesticide exposure | AD (NINCDS-ADRDA) | Not reported | Linear predictor | **Black or African American**  5Y: 0.86 (0.81–0.91)  6Y: 0.78 (0.74–0.83)  10Y: 0.71 (0.68–0.75)  15Y: 0.70 (0.67–0.74)  **White**  5Y: 0.86 (0.81–0.91)  6Y: 0.75 (0.7–0.81)  10Y: 0.74 (0.7–0.79)  15Y: 0.74 (0.69–0.78) | Not reported |
| N | Huque (2023) [9] | MAP | USA (No) | 843 (All-cause) & 848 (AD) | ≥60 | Median 5.0 (IQR: 1.0-2.0) | Partial (13 out of 15) | Age, sex, education, obesity (BMI), diabetes, depression, TBI, smoking, social engagement, PA, cognitive activity, alcohol intake & fish intake | Cholesterol, fish intake, & pesticide exposure | All-cause & AD (Computer algorithm) | NR | ROC curves | **All-cause**  0.65 (0.61-0.69)  **AD**  0.65 (0.61-0.69) | NR |
| N | Huque (2023) [9] | MAP | USA (No) | 1,472 | ≥60 | Median 5.0 (IQR: 1.0-2.0) | Partial (12 out of 15) | Age, sex, education, obesity (BMI), diabetes, depression, TBI, smoking, social engagement, PA, cognitive activity & alcohol intake | Cholesterol, fish intake & pesticide exposure | All-cause & AD (Computer algorithm) | NR | ROC curves | **All-cause**  0.66 (0.63-0.69) | NR |
| N | Huque (2023) [9] | CHS-CS | USA (No) | 3,097 (All-cause) & 1,692 (AD) | ≥65 | Median 6.0 (IQR 0.2-7.7) | Partial (9 out of 15) | Age, sex, education, diabetes, depression, smoking, alcohol intake, PA & fish intake | BMI, TBI, social engagement, cognitive activity, cholesterol & pesticide exposure | All-cause & AD (Consensus) | NR | ROC curves | **All-cause**  0.69 (0.66-0.72)  **AD**  0.71 (0.68-0.74) | NR |
| N | Huque (2023) [9] | CHS-CS | USA (No) | 3,273 | ≥65 | Median 6.0 (IQR 0.2-7.7) | Partial (8 out of 15) | Age, sex, education, depression, smoking, alcohol intake, PA & fish intake | BMI, TBI, diabetes, social engagement, cognitive activity, cholesterol & pesticide exposure | All-cause & AD (Consensus) | NR | ROC curves | **All-cause**  0.69 (0.66-0.71) | NR |
| N | Huque (2023) [9] | HRS-ADAMS | USA (No) | 421 (All-cause) & 424 (AD) | ≥70 | Median 5.0 (IQR: 2.0-6.0) | Partial (9 out of 15) | Age, sex, education, diabetes, depression, TBI, smoking, cognitive activity & alcohol intake | BMI, fish intake, social engagement, PA, cholesterol & pesticide exposure | All-cause & AD (DSM-III-R & DSM-IV) | NR | ROC curves | **All-cause**  0.66 (0.59-0.73)  **AD**  0.66 (0.58-0.75) | NR |
| N | Huque (2023) [9] | HRS-ADAMS | USA (No) | 432 | ≥70 | Median 5.0 (IQR: 2.0-6.0) | Partial (8 out of 15) | Age, sex, education, diabetes, depression, TBI, smoking & cognitive activity | BMI, fish intake, alcohol intake, social engagement, PA, cholesterol & pesticide exposure | All-cause & AD (DSM-III-R & DSM-IV) | NR | ROC curves | **All-cause**  0.66 (0.59-0.73) | NR |
| N | Kivimäki (2023) [36] | UKB | UK (No) | 465,929 | 38-73 | 10 | Full (15) | Age, sex, education, BMI, diabetes, depression, cholesterol, TBI, smoking, alcohol, social engagement, PA, cognitive activity, fish intake, and pesticide exposure | None | All-cause, AD, VaD, FTD & PPD (ICD-10) | n=3,421 | Cox | **All-cause**  0.59 (0.58-0.60)  **AD**  0.57 (0.55-0.59)  **VaD**  0.64 (0.61-0.67)  **FTD**  0.54 (0.47-0.61)  **PPD**  0.55 (0.49-0.60) | NR |
| N | Licher (2018) [27] | Rotterdam Study | Netherlands (No) | 6,667 | ≥55 | 15 | Partial (13 out of 15) | Age, sex, education, BMI, diabetes, depression, cholesterol, TBI, smoking, alcohol, social engagement, PA & fish intake | Cognitive activity & pesticide exposure | All-cause (DSM-III-R) and AD (NINCDS-ADRDA) | n=867 (All-cause) n=696 (AD) | Cox and LR | **All-cause**  **2yrs**  0.81 (0.77–0.86)  **5yrs**  0.78 (0.76–0.81)  **10yrs**  0.75 (0.74–0.77)  **15yrs**  0.70 (0.69–0.72)  **AD**  **2yrs**  0.80 (0.75-0.85)  **5yrs**  0.78 (0.75-0.80)  **10yrs**  0.75 (0.73-0.77)  **15yrs**  0.69 (0.68-0.71) | NR |
| N | Stephan (2020) [32] | 10/66 Study | China | 1,784 | ≥65 | 3-5 | Partial (12 out of 15) | Age, sex, education, BMI, diabetes, depression, TBI, smoking, alcohol, social engagement, PA & fish intake | Cognitive activity, pesticide exposure & cholesterol | All-cause (10/66 Algorithm) | 11.0% | Cox | 0.69 (0.65-0.72) | Good |
| N | Stephan (2020) [32] | As above | Cuba | 1,681 | As above | 3-5 | Partial (13 out of 15) | Age, sex, education, BMI, diabetes, depression, TBI, smoking, alcohol, social engagement, PA, fish intake & cholesterol | Cognitive activity & pesticide exposure | As above | 8.6% | As above | 0.67 (0.63-0.71) | Good |
| N | Stephan (2020) [32] | As above | Dominican Republic | 1,031 | As above | 3-5 | As above | As above | As above | As above | 11.2% | As above | 0.66 (0.61-0.71) | Good |
| N | Stephan (2020) [32] | As above | Mexico | 1,263 | As above | 3-5 | As above | As above | As above | As above | 8.5% | As above | 0.71 (0.67-0.76) | Good |
| N | Stephan (2020) [32] | As above | Peru | 512 | As above | 3-5 | As above | As above | As above | As above | 4.3% | As above | 0.78 (0.68-0.88) | Good |
| N | Stephan (2020) [32] | As above | Puerto Rico | 1,085 | As above | 3-5 | As above | As above | As above | As above | 10.6% | As above | 0.69 (0.64-0.74) | Poor |
| N | Stephan (2020) [32] | As above | Venezuela | 440 | As above | 3-5 | As above | As above | As above | As above | 11.4% | As above | 0.72 (0.65-0.79) | Good |
| N | Vonk (2021) [29] | AGES-RS | Iceland (No) | 5,343 | 66-98 | 5-6 | Partial (12 out of 15) | Age, sex, education, diabetes, head trauma, depressive symptoms, alcohol, smoking, fish intake, PA, mental leisure activity & social leisure activity | BMI, cholesterol & pesticide exposure | All-cause & AD (consensus) | 20.6% | Cox | **All-cause**  0.73 (0.71-0.76)  **AD**  0.68 (0.64-0.71) | Unable to calibrate |
| N | Vonk (2021) [29] | AGES-RS | Iceland (No) | 5,343 | 66-98 | 5-6 | Partial (6 out of 15) | Age, sex, education, diabetes, alcohol & smoking | BMI, depression, cholesterol, TBI, social engagement, PA, cognitive activity, fish intake & pesticide exposure | As above | As above | As above | **All-cause**  0.71 (0.69-0.74)  **AD**  0.67 (0.63-0.70) | As above |
|  | **Model Validated**  Basic-DRM |  |  |  |  |  |  |  |  |  |  |  |  |  |
| Y | Licher (2019) [26] | EPOZ | Netherlands (No) | 514 | 60-90 | Median 9.5 (IQR: 7.6-11.4) | Full (4) | Age, stroke, subjective memory decline & IADL (needs help with finances or medication) | None | All-cause (DSM-III-R) | n=36 | Fine and Gray | 0.75 (0.67-0.82) | Well calibrated |
| Y | Stephan (2020) [32] | 10/66 Study | China | 1,813 | ≥65 | 3-5 (Mean 4.4 [1.2]) | As above | As above | As above | All-cause (10/66 Algorithm) | 11.3% | Cox | 0.71 (0.67-0.74) | Poor |
| Y | Stephan (2020) [32] | 10/66 Study | Cuba | 2,252 | ≥65 | 3-5 (Mean 4.0 [1.3]) | As above | As above | As above | As above | 7.7% | As above | 0.70 (0.66-0.74) | Poor |
| Y | Stephan (2020) [32] | 10/66 Study | Dominican Republic | 1,433 | ≥65 | 3-5 (Mean 4.3 [1.5]) | As above | As above | As above | As above | 11.2% | As above | 0.66 (0.62-0.70) | Poor |
| Y | Stephan (2020) [32] | 10/66 Study | Mexico | 1,516 | ≥65 | 3-5 (Mean 2.8 [0.6]) | As above | As above | As above | As above | 8.4% | As above | 0.71 (0.66-0.75) | Poor |
| Y | Stephan (2020) [32] | 10/66 Study | Peru | 1,307 | ≥65 | 3-5 (Mean 3.0 [0.8]) | As above | As above | As above | As above | 5.7% | As above | 0.79 (0.74-0.83) | Poor |
| Y | Stephan (2020) [32] | 10/66 Study | Puerto Rico | 1,363 | ≥65 | 3-5 (Mean 4.0 [1.2]) | As above | As above | As above | As above | 11.1% | As above | 0.72 (0.68-0.76) | Poor |
| Y | Stephan (2020) [32] | 10/66 Study | Venezuela | 1,331 | ≥65 | 3-5 (Mean 3.9 [1.1]) | As above | As above | As above | As above | 11.3% | As above | 0.74 (0.70-0.78) | Poor |
| Y | Vonk (2021) [29] | AGES-RS | Iceland (No) | 5,343 | 66-98 | 5-6 & 10 | As above | As above | As above | All-cause (consensus) | 20.6% | Fine and Gray | **5yrs**  0.75 (0.73-0.77)  **10yrs**  0.74 (0.72-0.76) | **5yrs** Good  **10yrs** Overestimated risk |
|  | **Model Validated**  Basic-DRM (Extended) |  |  |  |  |  |  |  |  |  |  |  |  |  |
| N | Licher (2019) [26] | EPOZ | Netherlands (No) | 514 | 60-90 | Median 9.5 (IQR: 7.6-11.4) | Full (12) | Age, age-squared, stroke, subjective memory decline & IADL (needs help with finances or medication) + Word Fluency Test (Words), Letter Digit Substitution Test (Letters), Delayed Word Learning Test (Words), APOE, total brain volume, hippocampal volume & total white matter hyperintensity volume | None | All-cause (DSM-III-R) | n=36 | Fine and Gray | 0.81 (0.74-0.88) | Well calibrated |
|  | **Model Validated**  BDSI |  |  |  |  |  |  |  |  |  |  |  |  |  |
| Y | Capuano (2022) [17] | Combined data: MAP, ROS & MARS | USA (No) | 2,357 | <80 | 3 | Full (7) | Age, education, underweight (BMI), diabetes, stroke, IADL (needs help with money or medications) & composite depressive symptom variable (antidepressant medication use or depressive symptoms) | As above | All-cause (NINDS & ADRDA) | NR | Fine-Gray | 0.72 (0.67-0.77)  (sample age <80 years) | Good fit |
| Y – 6Y Black and White Results | Dhana (2024) [42] | CHAP | USA (No) | 2,130 (1,159 Black or African American and 971 White) | 65-79  (6Y) or ≥65 (other follow-ups) | 5, 6, 10, 15 | Full | Age, education, BMI, diabetes, stroke, needs help with money and medications & depressive symptoms | None | AD (NINCDS-ADRDA) | Not reported | Linear predictor | **Black or African American**  5Y: 0.81 (0.76–0.85)  6Y: 0.79 (0.74–0.84)  10Y: 0.74 (0.71–0.77)  15Y: 0.73 (0.70–0.76)  **White**  5Y: 0.79 (0.74–0.85)  6Y: 0.77 (0.70–0.85)  10Y: 0.79 (0.75–0.82)  15Y: 0.78 (0.74–0.81) | Not reported |
| N | Downer (2016) [21] | MHAS | Mexico (Yes) | 3,002 | ≥60 | 11 | Partial | Age, education, diabetes, stroke, IADL (needs help with money or medications) & composite depressive symptom variable (antidepressant medication use or depressive symptoms) | Underweight (BMI) | All-cause (NIA-AA and CCCE) | NR | LR | 0.72 (0.69-0.77) | NR |
| Y | Kivimäki (2023) [36] | UKB | UK (No) | 465,929 | 38-73 | 10 | Full | Age, education, underweight (BMI), diabetes, stroke, IADL (needs help with money or medications) & composite depressive symptom variable (antidepressant medication use or depressive symptoms) | As above | All-cause, AD, VaD, FTD & PDD (ICD-10) | n=3,421 | Cox | **All-cause**  0.68 (0.67-0.69)  **AD**  0.68 (0.66-0.69)  **VaD**  0.73 (0.71-0.75)  **FTD**  0.59 (0.54-0.65)  **PPD**  0.66 (0.61-0.70) | NR |
| Y | Licher (2018) [27] | Rotterdam Study | Netherlands (No) | 6,667 | ≥55 | 15 | As above | As above | As above | All-cause (DSM-III-R) and AD (NINCDS-ADRDA) | n=867 (All-cause) n=696 (AD) | Cox and LR | **All-cause**  **2yrs**  0.83 (0.75–0.90)  **5yrs**  0.80 (0.76–0.84)  **10yrs**  0.78 (0.76–0.81)  **15yrs**  0.76 (0.74–0.78)  **AD**  **2yrs**  0.83 (0.75-0.90)  **5yrs**  0.81 (0.76-0.85)  **10yrs**  0.80 (0.77-0.83)  **15yrs**  0.78 (0.75-0.80) | NR |
| Y | Stephan (2020) [32] | 10/66 Study | China | 1,564 | ≥65 | 3-5 | As above | As above | As above | All-cause (10/66 Algorithm) | 9.0% | Cox | 0.67 (0.62-0.71) | Good |
| Y | Stephan (2020) [32] | 10/66 Study | Cuba | 1,751 | As above | 3-5 | As above | As above | As above | As above | 6.3% | As above | 0.66 (0.62-0.71) | Good |
| Y | Stephan (2020) [32] | 10/66 Study | Dominican Republic | 1,074 | As above | 3-5 | As above | As above | As above | As above | 9.1% | As above | 0.62 (0.57-0.68) | Good |
| Y | Stephan (2020) [32] | 10/66 Study | Mexico | 1,197 | As above | 3-5 | As above | As above | As above | As above | 6.6% | As above | 0.71 (0.65-0.76) | Good |
| Y | Stephan (2020) [32] | 10/66 Study | Peru | 994 | As above | 3-5 | As above | As above | As above | As above | 2.9% | As above | 0.78 (0.71-0.86) | Good |
| Y | Stephan (2020) [32] | 10/66 Study | Puerto Rico | 866 | As above | 3-5 | As above | As above | As above | As above | 7.4% | As above | 0.68 (0.62-0.74) | Poor |
| Y | Stephan (2020) [32] | 10/66 Study | Venezuela | 914 | As above | 3-5 | As above | As above | As above | As above | 8.8% | As above | 0.68 (0.62-0.74) | Good |
| Y | Vonk (2021) [29] | AGES-RS | Iceland (No) | 5,343 | 66-98 | 5-6 | As above | As above | As above | All-cause (consensus) | 20.6% | As above | 0.72 (0.70-0.75) | Overestimated for the highest group |
|  | **Model Validated**  CAIDE |  |  |  |  |  |  |  |  |  |  |  |  |  |
| N | Anstey (2014) [14] | CVHS | USA (No) | 2496 | ≥62 | Median 6.0 | Not clear | Not clear | Not clear | All-cause (study-specific criteria) & AD (NINCDS-ADRDA) | 11.1% (AD) | Cox | **Any Dementia**  **Overall**  0.57 (0.54-0.60)  **No BMI**  0.59 (0.56-0.62)  **No BMI/Cholesterol** 0.60 (0.57-0.63)  **AD**  **Overall**  0.57 (0.54-0.60)  **No BMI**  0.58 (0.55-0.61)  **No BMI/Cholesterol** 0.58 (0.55-0.62) | NR |
| N | Anstey (2014) [14] | MAP | USA (No) | 903 | ≥54 | Mean 3.5 | Not clear | Not clear | Not clear | All-cause & AD (NINCDS-ADRA) | 17·5% (AD) | As above | **Any Dementia**  **Overall**  0.49 (0.43-0.55)  **No BMI**  0.54 (0.48-0.61)  **No BMI/Cholesterol** 0.55 (0.51-0.59)  **AD**  **Overall**  0.49 (0.43-0.55)  **No BMI**  0.54 (0.48-0.60)  **No BMI/Cholesterol**  0.55 (0.51-0.59) | NR |
| N | Anstey (2014) [14] | KP | Sweden (No) | 905 | ≥74 | Mean 6.0 | Not clear | Not clear | Not clear | All-cause & AD (DSM-III-R) | 20·0% (AD) | Cox | **Any Dementia**  **Overall**  0.54 (0.50-0.58)  **No BMI**  0.54 (0.50-0.57)  **AD**  **Overall**  0.53 (0.49-0.58)  **No BMI**  0.53 (0.49-0.57) | NR |
| No use results from Kivimi (UKB) and Fayoose from WHII | Anaturk (2023) [33] | UKB – Training dataset | UK (No) | 176,611 | ≥50 | 14 | Full | Age, sex, education, PA, SBP, BMI & total cholesterol | None | All-cause (ICD-9, ICD-10, self-reported, primary/secondary cause of death or use of Donepezil,  Rivastigmine, Galantamine,  Modafinil or Memantine) | 1.7% (n=3,813) [total sample] | Fine Gray | 0.60 (0.60-0.60) | NR |
| N | Anaturk (2023) [33] | UKB – Testing dataset | UK (No) | 44,151 | Median 57 (IQR 10) | 14 | As above | As above | As above | As above | As above | As above | 0.60 (0.58-0.63) | NR |
| N | Anaturk (2023) [33] | UKB – Testing dataset | UK (No) | As above | As above | 1-5 | As above | As above | As above | As above | n=99 | As above | 0.56 (0.51-0.61) | NR |
| N | Anaturk (2023) [33] | UKB – Testing dataset | UK (No) | As above | As above | 5-10 | As above | As above | As above | As above | n=401 | As above | 0.58 (0.55-0.60) | NR |
| N | Anaturk (2023) [33] | Whitehall II | UK (No) | 2,934 | Median 57 (IQR 10) | 14 | As above | As above | As above | All-cause (self-report & hospital in-patient records: ICD-9 & ICD-10) | 3.2% (n=93) | As above | 0.69 (0.64-0.74) | NR |
| Y – 20Y follow-up only for Black and White | Dhana (2024) [42] | CHAP | USA (No) | 2,130 (1,159 Black or African American and 971 White) | ≥65 | 5, 10, 15, 20 | Full | As above | None | AD (NINCDS-ADRDA) | Not reported | Linear predictor | **Black or African American**  5Y: 0.58 (0.51–0.65)  10Y: 0.56 (0.52–0.61)  15Y: 0.56 (0.52–0.59)  20Y: 0.55 (0.51–0.58)  **White**  5Y: 0.55 (0.48–0.63)  10Y: 0.54 (0.48–0.59)  15Y: 0.53 (0.48–0.57)  20Y: 0.53 (0.48–0.57) | Poor |
| N | Exalto (2014) [15] | KPNC | USA (No) | 9,480 | 40-55 | Mean 36.1 | Partial (6 out of 7) | Age, sex, education, cholesterol, BMI & SBP | PA | All-cause (ICD-9), AD (ICD-9) a& VaD (ICD-9) | 25% | Cox and LR | **Specialist confirmed [Cox]**  0.75  **Specialist confirmed [LR]** 0.67  **Any dementia** 0.67 | Poor |
| Y | Fayosse (2020) [22] | Whitehall II | UK (No) | 7,553 | 39-63 | Mean 23.5 (4.0) | Full | Age, sex, education, PA, SBP, BMI & total cholesterol | None | All-cause (ICD-10) and hospital record | n=318 | Cox and LR | **LR**  0.80 (0.78-0.82)  **Cox**  0.71 (0.69-0.74) | Poor |
| N | Huque (2023) [9] | CHS-CS | USA (No) | 3,097 | ≥65 | Median 6.0 (IQR 0.2-7.7) | Partial (4 out of 7) | Age, sex, education & PA | BMI, total cholesterol & SBP | All-cause (Consensus) | NR for analytical sample | ROC curves | 0.57 (0.52-0.61) | NR |
| Y – but not all cause only subtypes | Kivimäki (2023) [36] | UKB | UK (No) | 465,929 | 38-73 | 10 | Full | Age, sex, education, PA, SBP, BMI & total cholesterol | None | All-cause, AD, VaD, FTD & PPD (ICD-10) | n=3,421 | Cox | **All-cause**  0.66 (0.65-0.67)  **AD**  0.66 (0.65-0.67)  **VaD**  0.70 (0.68-0.72)  **FTD**  0.61 (0.57-0.66)  **PDD**  0.63 (0.60-0.67) | NR |
| N (use Fayosse) | Kivimäki (2023) [36] | Whitehall II | UK (No) | 4,865 | 45-69 | 20 | As above | As above | As above | All-cause | n=202 | As above | 0.65 (0.61-0.68) | NR |
| Y | Licher (2018) [27] | Rotterdam Study | Netherlands (No) | 6,667 | ≥55 | 15 | As above | As above | As above | All-cause (DSM-III-R) and AD (NINCDS-ADRDA) | n=867 (All-cause) n=696 (AD) | Cox and LR | **All-cause**  **2yrs**  0.49 (0.42–0.56)  **5yrs**  0.54 (0.50–0.58)  **10yrs**  0.55 (0.53–0.58)  **15yrs**  0.55 (0.53–0.57)  **AD**  **2yrs**  0.51 (0.43-0.59)  **5yrs**  0.54 (0.50-0.58)  **10yrs**  0.55 (0.53-0.58)  **15yrs**  0.55 (0.53-0.57) | Underestimated risk |
| N – same as data below, just different sample size | Trares (2024) [10] | ESTHER Study | Germany (Yes) | 1,918 | 50-75 | 20 (Median 16.3; IQR (13.5–17.0) | As above | As above | As above | All-cause, AD and VaD (GP diagnosis) | n=562 (173 AD & 199 VaD) | LR | **TOTAL COHORT**  **All-cause**  0.70 (0.67-0.73)  **AD**  0.70 (0.65-0.75)  **VaD**  0.70 (0.65-0.75)  **MID-LIFE (50-64)**  **All-cause** 0.70 (0.65-0.74)  **AD** 0.70 (0.62-0.78)  **VaD** 0.67 (0.59-0.74)  **LATE-LIFE (65-75)**  **All-cause** 0.58 (0.54-0.63)  **AD** 0.58 (0.47-0.66)  **VaD** 0.56 (0.47-0.64) | NR |
| Y | Trares (2024) [10] | ESTHER Study | Germany (Yes) | 5,360 | 50-75 | 17 (Mean 14.8; SD 3.5) | Full | Age, sex, education, systolic blood pressure, BMI, total cholesterol & PA | None | All-cause, AD and VaD (GP diagnosis) | n=410 (139 AD & 152 VaD) | Cox | **TOTAL COHORT**  **All-cause**  0.79  (0.77–0.81)  **AD**  0.79  (0.76–0.83)  **VaD**  0.79  (0.76–0.82)  **MID-LIFE (50-64)**  **All-cause** 0.73 (0.69-0.77)  **AD** 0.77 (0.71-0.83)  **VaD** 0.76 (0.70-0.81)  **LATE-LIFE (65-75)**  **All-cause** 0.67 (0.64-0.7)  **AD** 0.69 (0.63-0.75)  **VaD** 0.67 (0.62-0.72) | Good (all models) |
| Y | Shang (2022) [38] | UKB | UK (No) | 471,485 | 38-73 | Median11.9 (IQR 11.2-12·6) | As above | As above | As above | All-cause (ICD-9, ICD-10 and self-reported fields) | n=6,189 | Cox | 0.70 (0.70-0.71) | NR |
| N | Stephan (2020) [32] | 10/66 Study | China (No) | 1,822 | ≥65 | 3-5 | Partial (6 out of 7) | Age, sex, education, systolic blood pressure, BMI & PA | Total cholesterol | All-cause (10/66 Algorithm) | 11.2% | Cox | 0.55 (0.51-0.59) | Good |
| Y | Stephan (2020) [32] | 10/66 Study | Cuba (No) | 1,798 | As above | 3-5 | Full (7) | Age, sex, education, systolic blood pressure, BMI, total cholesterol & PA | None | As above | 8.6% | As above | 0.57 (0.52-0.61) | Good |
| Y | Stephan (2020) [32] | 10/66 Study | Dominican Republic (No) | 1,064 | As above | 3-5 | As above | As above | As above | As above | 11.3% | As above | 0.52 (0.47-0.57) | Good |
| Y | Stephan (2020) [32] | 10/66 Study | Mexico (No) | 1,281 | As above | 3-5 | As above | As above | As above | As above | 8.6% | As above | 0.58 (0.53-0.63) | Good |
| Y | Stephan (2020) [32] | 10/66 Study | Peru (No) | 542 | As above | 3-5 | As above | As above | As above | As above | 4.0% | As above | 0.63 (0.50-0.75) | Good |
| Y | Stephan (2020) [32] | 10/66 Study | Puerto Rico (No) | 1,089 | As above | 3-5 | As above | As above | As above | As above | 10.6% | As above | 0.55 (0.50-0.61) | Good |
| Y | Stephan (2020) [32] | 10/66 Study | Venezuela (No) | 763 | As above | 3-5 | As above | As above | As above | As above | 12.2% | As above | 0.53 (0.48-0.59) | Good |
| N | Zheng (2023) [41] | UKB | UK (No) | 429,033 | 38-79 | Median 12.8 | Full | Age, sex, smoking, SBP, diabetes, total cholesterol and, HDL cholesterol (and interactions between risk factors and age) | None | All-cause (n=6,477), AD (n=2,726) & VaD (1,439) IICD-9 & ICD-10) | **All-cause** n=6,477  **AD** n=2,726  **VaD** n=1,439 | Cox | **All-cause**  0.66 (0.65-0.66)  **AD**  0.66 (0.65-0.67)  **VaD**  0.70 (0.68-0.71) | NR |
|  | **Model Validated**  CAIDE + APOE |  |  |  |  |  |  |  |  |  |  |  |  |  |
| Y – 20Y only Black and White results | Dhana (2024) [42] | CHAP | USA (No) | 2,130 (1,159 Black or African American and 971 White) | ≥65 | 5, 10, 15, 20 | Full | Age, sex, education, SBP, BMI, total cholesterol, PA & APOE | None | AD (NINCDS-ADRDA) | Not reported | Linear predictor | **Black or African American**  5Y: 0.58 (0.52–0.65)  10Y: 0.58 (0.54–0.63)  15Y: 0.58 (0.54–0.62)  20Y: 0.57 (0.54–0.61)  **White**  5Y: 0.61 (0.53–0.69)  10Y: 0.60 (0.55–0.65)  15Y: 0.59 (0.54–0.64)  20Y: 0.59 (0.54–0.63) | Not reported |
| Y | Kivimäki (2023) [36] | UKB | UK (No) | 465,929 | 38-73 | 10 | Full | As above | As above | All-cause, AD, VaD, FTD & PDD (ICD-10) | n=3,421 | Cox | **All-cause**  0.73 (0.72-0.73)  **AD**  0.74 (0.73-0.76)  **VaD**  0.76 (0.74-0.77) **FTD**  0.64 (0.58-0.69)  **PDD**  0.69 (0.65-0.73) | NR |
| Y | Kivimäki (2023) [36] | Whitehall II | UK (No) | 4,865 | 45-69 | 20 | As above | As above | As above | All-cause | n=202 | As above | 0.74 (0.70-0.78) | NR |
| N – same as data below (just different sample size) | Trares (2024) | ESTHER Study | Germany (Yes) | 1,918 | 50-75 | 20 | As above | As above | As above | All-cause, AD and VaD (GP diagnosis) | n=562 (173 AD & 199 VaD) | LR | **TOTAL COHORT**  **All-cause**  0.73 (0.70-0.76)  **AD**  0.75 (0.70-0.80)  **VaD**  0.71 (0.66-0.75)  **MID-LIFE (50-64)**  **All-cause** 0.72 (0.67-0.77)  **AD** 0.75 (0.68-0.83**)**  **VaD** 0.67 (0.59-0.75)  **LATE-LIFE (65-75)**  **All-cause** 0.62 (0.57-0.68)  **AD** 0.65 (0.57-0.72)  **VaD** 0.58 (0.49-0.67) | NR |
| Y | Trares (2024) [10] | ESTHER Study | Germany (Yes) | 5,360 | 50-75 | Mean 14.8 (SD 3.5) | As above | As above | As above | All-cause, AD and VaD (GP diagnosis) | n=410 (139 AD & 152 VaD) | Cox | **TOTAL COHORT All-cause**  0.80 (0.78–0.82)  **AD**  0.83 (0.80–0.86)  **VaD**  0.79 (0.76–0.83)  **MID-LIFE (50-64)**  **All-cause** 0.76 (0.72-0.80)  **AD** 0.81 (0.74-0.88)  **VaD** 0.76 (0.71-0.82)  **LATE-LIFE (65-75)**  **All-cause** 0.70 (0.67-0.73)  **AD** 0.74 (0.69-0.79)  **VaD** 0.68 (0.63-0.74) | Good (all models) |
|  | **Model Validated**  CARS |  |  |  |  |  |  |  |  |  |  |  |  |  |
| N | Fung (2024) [43] | HK-MAPS | Hong Kong (No) | 383 | Mean 69.9 (SD 6.4) | Mean 5.4 (SD 0.3) | Full | Age, sex, education, poorly controlled diabetes, prolonged sleep latency, mind body exercise, light exercise, loneliness & APOE | None | All-cause (cognitive z-score criteria) | n=70 (18.3%) | LR | 0.71 (0.60-0.80) | NR |
|  | **Model Validated**  CLHLS Risk Model |  |  |  |  |  |  |  |  |  |  |  |  |  |
| N | Hu (2022) [24]  NOTE Used temporal validation | CLHLS (2002-2008 cohort) | China (Yes) | 9,240 | ≥65 | Median 5.7 | Full | Age, sex, education, marital status, activity duration, leisure activity (i.e., playing cards or mah-jongg, watching TV or listening to the radio), stroke & cardiovascular disease | None | Chinese MMSE≤17 | NR | Cox & Fine-Gray | **Cox**  0.69 (0.67-0.70)  **Fine-Gray**  0.67 (0.65-0.69) | NR |
|  | **Model Validated**  CLHLS Risk Model-Illiterate Women |  |  |  |  |  |  |  |  |  |  |  |  |  |
| N | Chen (2023) [34] | CLHLS (2014-2018 cohort) | China (Yes) | 1,060 | ≥60 (women & illiterate) | Median 4.1 (IQR 3.5-4.3) | Full (7) | Age, MMSE, waist-to-height ratio, psychological score, ADL, IADL and frequency of tooth brushing | None | Chine MMSE<18 | 24.8% (n=263) | ROC curve | 0.74 | NR |
|  | **Model Validated**  CogDrisk |  |  |  |  |  |  |  |  |  |  |  |  |  |
| N | Huque (2023) [9] | RMAP | USA (No) | 843 | ≥60 | Median 5.0 (IQR: 1.0-2.0) | Partial (14 out of 17) | Age, sex, education, obesity, diabetes, depression, TBI, smoking, loneliness, PA, cognitive activity, fish intake, stroke & hypertension | Cholesterol, AF & insomnia | All-cause & AD (Computer algorithm) | NR | ROC curves | **All-cause**  0.65 (0.61-0.69) | NR |
| N | Huque (2023) [9] | RMAP | USA (No) | 843 | ≥60 | Median 5.0 (IQR: 1.0-2.0) | Partial (13 out of 17) | Age, sex, education, obesity, diabetes, depression, TBI, smoking, loneliness, PA, cognitive activity, stroke & hypertension | Cholesterol, fish intake, AF & insomnia | All-cause & AD (Computer algorithm) | NR | ROC curves | **All-cause**  0.66 (0.63-0.69) | NR |
| N | As above | CHS-CS | USA (No) | 3,097 | ≥65 | Median 6.0 (IQR 0.2-7.7) | Partial (12 out of 17) | Age, sex, education, diabetes, depression, smoking, PA, loneliness, insomnia, stroke, AF & fish intake | Hypertension, obesity, cholesterol, TBI & cognitive engagement | All-cause & AD (Consensus) | NR | ROC curves | **All-cause**  0.70 (0.67-0.72) | NR |
| N | As above | CHS-CS | USA (No) | 3,273 | ≥65 | Median 6.0 (IQR 0.2-7.7) | Partial (11 out of 17) | Age, sex, education, depression, smoking, PA, loneliness, insomnia, stroke, AF & fish intake | Hypertension, diabetes, obesity, cholesterol, TBI & cognitive engagement | All-cause & AD (Consensus) | NR | ROC curves | **All-cause**  0.70 (0.67-0.72) | NR |
| N | As above | HRS-ADAMS | USA (No) | 421 | ≥70 | Median (5.0 (IQR: 2.0-6.0) | Partial (10 out of 17) | Age, sex, education, diabetes, depression, TBI, smoking, cognitive activity, insomnia & stroke | Hypertension, obesity, cholesterol, PA, AF, social engagement & fish Consumption (diet) | All-cause & AD (DSM-III-R & DSM-IV) | NR | ROC curves | **All-cause**  0.65 (0.58-0.72) | NR |
| N | As above | HRS-ADAMS | USA (No) | 432 | ≥70 | Median (5.0 (IQR: 2.0-6.0) | Partial (8 out of 17) | Age, sex, education, diabetes, depression, TBI, smoking & cognitive activity | Hypertension, obesity, insomnia, stroke, cholesterol, PA, AF, social engagement & fish Consumption (diet) | All-cause & AD (DSM-III-R & DSM-IV) | NR | ROC curves | **All-cause**  0.64 (0.57-0.7) | NR |
| N | Kootar (2023) [37] | SNAC-K | Sweden (No) | 1,473 | 60-104 | 2001-04 to 2018 | Partial (15 out of 17) | Age, sex, education, hypertension, diabetes, PA, depression, TBI, atrial fibrillation, smoking, social engagement, cognitive engagement, fish consumption (diet), stroke & insomnia | Midlife obesity & midlife high cholesterol | All-cause | 8.2% | AUC | 0.77 (0.57-0.97) | NR |
| N | Kootar (2023) [37] | SNAC-K | Sweden (No) | 1,987 | As above | As above | Partial (13 out of 17) | Age, sex, education, diabetes, stroke, hypertension, smoking, TBI, AF, depression, social engagement, cognitive activity & fish intake | Midlife obesity, midlife high cholesterol, PA & insomnia | As above | As above | As above | 0·76 (0·63-0·88) | NR |
| N | Kootar (2023) [37] | SNAC-K | Sweden (No) | 584 | As above | As above | Partial (16 out of 17) | Age, sex, education, hypertension, midlife obesity, diabetes, PA, depression, TBI, AF, smoking, social engagement, cognitive engagement, fish consumption (diet), stroke, and insomnia | Midlife high cholesterol | As above | As above | As above | 0.51 (0.27-0.75) | NR |
| N | Kootar (2023) [37] | HRS-ADAMS | USA (No) | 229 | 70-110 | 2001-2003 to 2008-2009 | Partial (11 out of 17) | Age, sex, education, diabetes, depression, TBI, AF, smoking, cognitive engagement, stroke & insomnia | Hypertension, PA, midlife obesity, midlife high cholesterol, social engagement & fish consumption (diet) | All-cause | 51.6% | As above | 0.76 (0.70-0.83) | NR |
| N | Kootar (2023) [37] | HRS-ADAMS | USA (No) | 586 | As above | As above | Partial (9 out of 17) | Age, sex, education, diabetes, stroke, smoking, TBI, depression & cognitive activity | Hypertension, midlife obesity, midlife high cholesterol, PA, AF, social engagement, fish consumption (diet) & insomnia | As above | As above | As above | 0·75 (0·71-0·79) | NR |
| N | Kootar (2023) [37] | CHS-CS | Canada | 3226 | 65-97 | 1991-1994 to 1998-1999 | Partial (12 out of 17) | Age, sex, education, diabetes, depression, AF, smoking, social engagement, fish consumption (diet), stroke, PA & insomnia | Hypertension, midlife obesity, midlife high cholesterol, TBI & cognitive engagement | All-cause | 14.2% | As above | 0.70 (0.67-0.72) | NR |
| N | Kootar (2023) [37] | CHS-CS | Canada | 3,295 | As above | As above | Partial (11 out of 17) | Age, sex, education, stroke, smoking, AF, insomnia, depression, social engagement, PA & fish intake | Hypertension, diabetes, midlife obesity, midlife high cholesterol, TBI & cognitive engagement | As above | As above | As above | 0·70 (0·67-0·72) | NR |
| N | Kootar (2023) [37] | MAP | USA | 850 | 54-100 | 1997-1998 to 2020 | Partial (14 out of 17) | Age, sex, education, hypertension, midlife obesity, diabetes, PA, depression, TBI, smoking, social engagement, cognitive engagement, fish consumption (diet) & stroke | Midlife high cholesterol, AF & insomnia | All-cause | 27.0% | As above | 0.66 (0.62-0.70) | NR |
| N | Kootar (2023) [37] | MAP | USA | 1,478 | As above | As above | Partial (13 out of 17) | Age, sex, education, obesity, diabetes, stroke, TBI, hypertension, smoking, depression, social engagement, PA & cognitive activity | Midlife high cholesterol, AF, fish consumption (diet) & insomnia | As above | As above | As above | 0·67 (0·64-0·70) | NR |
|  | **Model Validated**  CogDrisk-AD |  |  |  |  |  |  |  |  |  |  |  |  |  |
| N | Huque (2023) [9] | RMAP | USA (No) | 843 | ≥60 | Median 5.0 (IQR: 1.0-2.0) | Partial (14 out of 16) | Age, sex, education, obesity, diabetes, depression, TBI, smoking, loneliness, PA, cognitive activity, fish intake, stroke & hypertension | Midlife high cholesterol & pesticide exposure | AD (Computer algorithm) | NR | ROC curves | 0.65 (0.61- 0.69) | NR |
| N | Huque (2023) [9] | HRS-ADAMS | USA (No) | 424 | ≥70 | Median (5.0 (IQR: 2.0-6.0) | Partial (9 out of 16) | Age, sex, education, diabetes, depression, TBI, smoking, cognitive activity & stroke | Hypertension, midlife obesity, midlife high cholesterol, PA, social engagement, fish intake & pesticide exposure | AD (DSM-III-R & DSM-IV) | NR | ROC curves | 0.67 (0.59-0.75) | NR |
| N | Huque (2023) [9] | CHS-CS | USA (No) | 2,692 | ≥65 | Median 6.0 (IQR 0.2-7.7) | Partial (10 out of 16) | Age, sex, education, diabetes, depression, smoking, PA, loneliness, stroke & fish intake | Hypertension, midlife obesity, midlife high cholesterol, social engagement, cognitive activity & pesticide exposure | AD (DSM-III-R & DSM-IV) | NR | ROC curves | 0.72 (0.69-0.75) | NR |
| N | Kootar (2023) [37] | SNAC-K | Sweden (No) | 1,579 | 60-104 | 2001-04 to 2018 | Partial (13 out of 16) | Age, sex, education, diabetes, stroke, hyperten­sion, smoking, TBI, depres­sion, social engagement, PA, cognitive activity & fish intake | Midlife obesity, midlife high cholesterol & pesticide exposure | AD | NR | AUC | 0.69 (0.60-0.78) | NR |
| N | Kootar (2023) [37] | As above | As above | 2,137 | As above | As above | Partial (12 out of 16) | Age, sex, education, diabetes, stroke, hyperten­sion, smoking, TBI, depres­sion, social engagement, cognitive activity & fish intake | Midlife obesity, midlife high cholesterol, PA & pesticide exposure | As above | NR | As above | 0.69 (0.61-0.76) | NR |
| N | Kootar (2023) [37] | HRS ADAMS | USA (No) | 594 | 70-110 | 2001-2003 to 2008-2009 | Partial (9 out of 16) | Age, sex, education, diabetes, stroke, smoking, TBI, depression & cognitive activity | Hypertension, midlife obesity, midlife high cholesterol, PA, social engagement, fish intake & pesticide exposure | As above | NR | As above | 0.72 (0.68-0.77) | NR |
| N | Kootar (2023) [37] | HRS ADAMS | USA (No) | 671 | As above | As above | Partial (8 out of 16) | Age, sex, education, diabetes, stroke, smoking, TBI & depression | Hypertension, midlife obesity, midlife high cholesterol, cognitive activity, PA, social engagement, fish intake & pesticide exposure | As above | NR | As above | 0.71 (0.67-0.75) | NR |
| N | Kootar (2023) [37] | CHS-CH | Canada | 2,701 | 65-97 | 1991-1994 to 1998-1999 | Partial (10 out of 16) | Age, sex, education, diabetes, stroke, smoking, de­pression, social engagement, PA & fish intake | Hypertension, midlife obesity, midlife high cholesterol, TBI, cognitive engagement & pesticide exposure | As above | NR | As above | 0.72 (0.69-0.75) | NR |
| N | Kootar (2023) [37] | As above | As above | 2,757 | As above | As above | Partial (9 out of 16) | Age, sex, education, stroke, smoking, depression, social engagement, PA & fish intake | Hypertension, midlife obesity, midlife high cholesterol, diabetes, TBI, cognitive engagement & pesticide exposure | As above | NR | As above | 0.72 (0.69-0.74) | NR |
| N | Kootar (2023) [37] | MAP | USA | 850 | 54-100 | 1997-1998 to 2020 | Partial (13 out of 16) | Age, sex, educa­tion, diabetes, stroke, TBI, hypertension, smoking, de­pression, social engagement, PA, cognitive activity & fish intake | Midlife obesity, midlife high cholesterol & pesticide exposure | As above | NR | As above | 0.66 (0.62-0.70) | NR |
| N | Kootar (2023) [37] | MAP | USA | 1,511 | As above | As above | Partial (12 out of 16) | Age, sex, educa­tion, diabetes, stroke, TBI, hypertension, smoking, de­pression, social engagement, PA & cognitive activity | Midlife obesity, midlife high cholesterol, fish intake & pesticide exposure | As above | NR | As above | 0.67 (0.64-0.70) | NR |
|  | **Model Validated**  CSHA Derived Score [Hogan (2000) |  |  |  |  |  |  |  |  |  |  |  |  |  |
| N | Vonk (2021) [29] | AGES-RS | Iceland (No) | 5,343 | 66-98 | 5-6 | Full | Age, subjective memory concern & MMSE/3MS | None | All-cause (consensus) | 20.6% | LR | 0.80 (0.78-0.82) | Good |
|  | **Model Validated**  CTDS |  |  |  |  |  |  |  |  |  |  |  |  |  |
| N | Ben-Hassen (2022) [16] | 3-City Study | France (No) | 3,953 | Mean 73.2 (SD 5.0) | Median 9.7 (IQR: 5.6-15.4) | Full | IST, MMSE, and IADL (using the phone, using means of transport, taking medication as prescribed, and managing money) | None | All-cause (DSM-IV-R) | 15.2% | Joint models (Mixed-model and proportional hazards) | Range 0.85-0.96 (over 10-year prediction frame) | Well calibrated at 5-years |
|  | **Model Validated** Demographic + APOE |  |  |  |  |  |  |  |  |  |  |  |  |  |
| y | Chouraki (2016) [19] | 3-City Study | France (No) | 6,079 | ≥65 (Mean 74.2; SD 5.5) | 5-8 | Full | Age, sex, education & APOE status | None | DSM-IV | 7.7% | Cox | **5yrs**  0.76 (0.73-0.79)  **6yrs**  0.74 (0.72-0.77)  **7yrs**  0.75 (0.72-0.77)  **8yrs**  0.75 (0.73-0.77) | Calibration of the models at 7 years of follow-up was good across studies |
| y | Chouraki (2016) [19] | ACT Study | USA (No) | 2,110 | ≥65 (Mean (75.6; SD 6.5) | 5-8 | As above | As above | As above | As above | 19.8% | Cox | **5yrs**  0.82 (0.79-0.85)  **6yrs**  0.82 (0.79-0.85)  **7yrs**  0.79 (0.76-0.82)  **8yrs**  0.78 (0.75-0.81) | See above |
| y | Chouraki (2016) [19] | AGES-RS | Iceland (No) | 2,553 | ≥65 (Mean (75.5; SD 5.1) | 5-8 | As above | As above | As above | As above | 11.0% | Cox | **5yrs**  0.79 (0.72-0.85)  **6yrs**  0.73 (0.67-0.78)  **7yrs**  0.69 (0.61-0.74)  **8yrs**  0.67 (0.64-0.72) | See above |
| y | Chouraki (2016) [19] | CHS | USA (No) | 1,998 | ≥65 (Mean 74.8; SD 4.7) | 5-8 | As above | As above | As above | As above | 18.4% | Cox | **5yrs**  0.75 (0.71-0.78)  **6yrs**  0.75 (0.72-0.78)  **7yrs**  0.74 (0.71-0.78)  **8yrs**  0.71 (0.67-0.75) | See above |
| y | Chouraki (2016) [19] | FHS | USA (No) | 1,757 | ≥65 (Mean 76.1; SD 7.4) | 5-8 | As above | As above | As above | As above | 13.1% | Cox | **5yrs**  0.84 (0.81-0.86)  **6yrs**  0.82 (0.79-0.84)  **7yrs**  0.80 (0.78-0.83)  **8yrs**  0.80 (0.77-0.83) | See above |
| y | Chouraki (2016) [19] | ROSMAP | USA (No) | 1,262 | ≥65 (Mean 78.4; SD 7.1) | 5-8 | As above | As above | As above | As above | 28.4% | Cox | **5yrs**  0.77 (0.73-0.80)  **6yrs**  0.76 (0.73-0.79)  **7yrs**  0.76 (0.73-0.78)  **8yrs**  0.76 (0.74-0.79) | See above |
| y | Chouraki (2016) [19] | Rotterdam Study | Netherlands (No) | 3,334 | ≥65 (Mean 74.0; SD 6.5) | 5-8 | As above | As above | As above | As above | 18.2% | Cox | **5yrs**  0.78 (0.75-0.81)  **6yrs**  0.78 (0.75-0.81)  **7yrs**  0.78 (0.75-0.81)  **8yrs**  0.77 (0.74-0.80) | See above |
| y | Chouraki (2016) [19] | WHICAP | USA (No) | 594 | ≥65 (Mean 76.7; SD 6.8) | 5-8 | As above | As above | As above | As above | 8.8% | Cox | **5yrs**  0.77 (0.67-0.87)  **6yrs**  0.80 (0.71-0.88)  **7yrs**  0.78 (0.69-0.87)  **8yrs**  0.78 (0.69-0.86) | See above |
|  | **Model Validated**  Demographic-Cognition Model_4 |  |  |  |  |  |  |  |  |  |  |  |  |  |
| N | Vonk (2021) [29] | AGES-RS | Iceland (No) | 5,343 | 66-98 | 5-6 | Full | Age, sex, education & CVLT (Delayed memory recall) | None | AD (consensus) | 10.4% | LR | 0.81 (0.78-0.84) | Overestimated risk |
|  | **Model Validated**  DemPoRT |  |  |  |  |  |  |  |  |  |  |  |  |  |
| N | Fisher (2021) [23]  **temporal validation ** | CCHS (Ontario respondents 2009/2010 & 2011/2012) | Canada (No) | 27,721 | ≥55 | 5 | Full | Age, sex, education, ethnicity, marital status, neighbourhood deprivation, immigrant status, multilingualism, stress, sense of belonging, stress, self-rated health, smoking, alcohol (former drinker), fruit and vegetable consumption, potato consumption, juice consumption, leisure PA, function (combined score including: personal hygiene and care, locomotion in home, meal preparation, running errands, ordinary housework, finances), heart disease, stroke, diabetes, mood disorder, high blood pressure, COPD, epilepsy, BMI & survey year + interaction terms (n=24) | None | All-cause (Physician billing, hospitalisation & drug dispensing data) | n=1,714 | Fine-Gray | **Men**  0.83 (0.81-0.85)  **Women**  0.83 (0.81-0.85) | Underestimate risk at older ages, those who need help with daily activities and those with a history of stroke (both men and women) |
|  | **Model Validated**  DemRisk-young |  |  |  |  |  |  |  |  |  |  |  |  |  |
| N | Reeves (2024) [45] | CPRD Gold (158 GP practices) | UK (Yes) | 419,126 | 60-79 | 5 | Full | Age, age-squared, deprivation, depression, stroke, diabetes, epilepsy, gait problems, major head injury, selective serotonin reuptake inhibitor use, tricyclic antidepressant use, other antidepressant use, mood stabilisers, NSAIDs (excluding aspirin), anticholinergic burden over last year (square-root), ever received social services, number of A&E visits in last year (square root), number of GP consultations involving third party in last year (square root), number of GP home visits in last year (square root), number of missed GP appointments in last year (square root), most recent BMI (square root), and most recent mean systolic blood pressure value/20 | None | All-cause (Primary Care record or ICD-10) | n=7,425 | Cox | 0.78 (0.78-0.79) | Good |
|  | **Model Validated**  DemRisk-old |  |  |  |  |  |  |  |  |  |  |  |  |  |
| N | Reeves (2024) [45] | CPRD Gold (158 GP practices) | UK (Yes) | 118,717 | 80-89 | 5 | Full | Sex, age-80, age-80 squared, deprivation, stroke, diabetes, gait problems, major head injury, selective serotonin reuptake inhibitor use, tricyclic antidepressant use, other antidepressant use, NSAIDs (excluding aspirin), anticholinergic burden over last year (square-root), ever received social services, number of A&E visits in last year (square root), number of GP consultations involving third party in last year (square root), number of GP home visits in last year (square root), number of missed GP appointments in last year (square root), most recent BMI (square root), and most recent mean systolic blood pressure value/20 | None | All-cause (Primary Care record or ICD-10) | n=11,078 | Cox | 0.64 (0.63-0.64) | Good |
|  | **Model Validated**  DRS_young (60-79) |  |  |  |  |  |  |  |  |  |  |  |  |  |
| y | Anatürk (2023) [33] | UKB – Training dataset | UK (No) | 176,611 | ≥50 | 14 | Full | Age, age-squared, sex, calendar year, social deprivation, BMI, BMI-squared, current anti-hypertensive use, smoking status, alcohol misuse, depression/anti-depressant use, aspirin use, history of diabetes, history of stroke or TIA, & history of AF | None | All-cause (ICD-9, ICD-10, self-reported, primary/secondary cause of death or use of Donepezil,  Rivastigmine, Galantamine,  Modafinil or Memantine) | 1.7% (n=3,813) [total sample] | Fine Gray | 0.76 (0.76-0.76) | NR |
| N | Anatürk (2023) [33] | UKB – Testing dataset | UK (No) | 44,151 | Median 57 (IQR 10) | 14 | As above | As above | As above | As above | As above | As above | 0.77 (0.76-0.79) | NR |
| N | Anatürk (2023) [33] | UKB – Testing dataset | UK (No) | As above | As above | 1-5 | As above | As above | As above | As above | n=99 | As above | 0.73 (0.69-0.77) | NR |
| N | Anatürk (2023) [33] | UKB – Testing dataset | UK (No) | As above | As above | 5-10 | As above | As above | As above | As above | n=401 | As above | 0.73 (0.71-0.75) | NR |
| Y | Anatürk (2023) [33] | Whitehall II | UK (No) | 2,934 | Median 57 (IQR 10) | 14 | As above | As above | As above | All-cause (self-report & hospital in-patient records: ICD-9 & ICD-10) | 3.2% (n=93) | As above | 0.74 (0.69-0.78) | NR |
| N (NOTE: It is not clear if DRS-young or DRS-old was mapped) | Dhana (2024) [42] | CHAP | USA (No) | 2,130 (1,159 Black or African American and 971 White) | Not clear | 5 | Not clear | Not clear | Not clear | AD (NINCDS-ADRDA) | Not reported | Linear predictor | **Black or African American**  5Y: 0.75 (0.69–0.81)  **White**  5Y: 0.66 (0.58–0.75) | Not clear which DRS model calibrated (i.e., young vs. old) |
| N | John (2022) [25] | MDCR | USA (No) | 10M | 60-79 | 5 | Partial (14 out of 15) | Age, age-squared, sex, calendar year, BMI, BMI-squared, current anti-hypertensive use, smoking status, alcohol misuse, depression/anti-depressant use, aspirin use, history of diabetes, history of stroke or TIA, & history of AF | Social deprivation | NR | NR | pROC R-package | 0.69 (0.69–0.69) | Calibration E_avg_=0.060 |
| N | John (2022) [25] | IQGE | Germany (No) | 30M | 60-79 | 5 | Partial (14 out of 15) | As above | As above | NR | NR | pROC R-package | 0.75 (0.75–0.75) | Calibration E_avg_=0.025 |
| N | John (2022) [25] | OPSES | USA (No) | 85M | 60-79 | 5 | Partial (14 out of 15) | As above | As above | NR | NR | pROC R-package | 0.74 (0.74–0.74) | Calibration E_avg_=0.064 |
| N | John (2022) [25] | OPEHR | USA (No) | 94M | 60-79 | 5 | Partial (14 out of 15) | As above | As above | NR | NR | pROC R-package | 0.73 (0.73–0.73) | Calibration E_avg_=0.057 |
| N | John (2022) [25] | CPRD | UK (No) | 13M | 60-79 | 5 | Partial (14 out of 15) | As above | As above | NR | NR | pROC R-package | 0.67 (0.66–0.67) | Calibration E_avg_=0.073 |
| N | John (2022) [25] | IPCI | Netherlands (No) | 2.5M | 60-79 | 5 | Partial (14 out of 15) | As above | As above | NR | NR | pROC R-package | 0.76 (0.75–0.77) | Calibration E_avg_=0.024 |
| N | John (2022) [25] | IMRD | UK (No) | 18M | 60-79 | 5 | Partial (14 out of 15) | As above | As above | NR | NR | pROC R-package | 0.68 (0.68–0.69) | Calibration E_avg_=0.065 |
| Y | Reeves (2024) [45] | CPRD Gold (158 GP practices) | UK (Yes) | 419,126 | 60-79 | 5 | Full (but variables adapted to fit CPRD and recalibrated the model) | Age, age-squared, sex, calendar year, social deprivation, BMI, BMI-squared, current anti-hypertensive use, smoking status, alcohol misuse, depression/anti-depressant use, aspirin use, history of diabetes, history of stroke or TIA, & history of AF | None | All-cause (Primary Care record or ICD-10) | n=7,425 | Cox | 0.77 (0.77-0.78) | Not reported |
| Y | Licher (2018) [27] | Rotterdam Study | Netherlands (No) | 5,019 | 60-79 | 15 | Full (but adapted variables for the Townsend deprivation index & anxiety symptoms) | Age, age-squared, sex, calendar year, local area deprivation, BMI, BMI-squared, current anti-hypertensive use, smoking status, alcohol use, diabetes, current depression/anti-depressants, stroke/TIA, AF & aspirin use | Not applicable | All-cause (DSM-III-R) | All-cause  **2yrs** 34  **5yrs** 141  **10yrs** 345  **15yrs** 644 | Cox and LR | **2yrs**  0.80 (0.70-0.89)  **5yrs**  0.77 (0.72-0.81)  **10yrs**  0.75 (0.72-0.78)  **15yrs**  0.73 (0.71-0.75) | Predictions too extreme, particularly for those at high predicted risk |
| y | Walters (2016) [31] | THIN | UK (No) | 226,140 | 60-79 | 5 | Full | Age, age-squared, sex, calendar year, local area deprivation, BMI, BMI-squared, current anti-hypertensive use, smoking status, history of an alcohol problem, history of diabetes, current depression/use of anti-depressant, current aspirin use, history of stroke or TIA & history of AF | None | All-cause including AD, vascular dementia, and unspecified or mixed dementia (ICD-10) | n=1,699 | Cox  * Reports Uno’s c index | 0.84 (0.81-0.87) | Good |
|  | **Model Validated**  DRS-old (80-95) |  |  |  |  |  |  |  |  |  |  |  |  |  |
| N (NOTE: It is not clear if DRS-young or DRS-old was mapped) | Dhana (2024) [42] | CHAP | USA (No) | 2,130 (1,159 Black or African American and 971 White) | 80-95 | Not clear | Not clear | Not clear | Not clear | AD (NINCDS-ADRDA) | Not reported | Linear predictor | **Black or African American**  5Y: 0.61 (0.48–0.73)  **White**  5Y: 0.55 (0.44–0.65) | Not clear which DRS model calibrated (i.e., young vs. old) |
| Y | Licher (2018) [27] | Rotterdam Study | Netherlands (No) | 709 | 80-95 | 15 | Full (but adapted variables for the Townsend deprivation index & anxiety symptoms) | Age, age-squared, sex, calendar year, BMI, current anti-hypertensive use, SBP, lipid ratio, smoking status, alcohol use, diabetes, stroke/TIA, AF, depression, anxiolytic drug use, current aspirin use & NSAID use | Not applicable | All-cause (DSM-III-R) and AD (NINCDS-ADRDA) | **2yrs** 29  **5yrs** 91  **10yrs** 167  **15yrs** 192 | Cox and LR | **2yrs** 0.60 (0.49-0.71)  **5yrs** 0.56 (0.50-62)  **10yrs** 0.56 (0.51-0.61)  **15yrs** 0.56 (0.51-0.60) | Predictions too extreme, particularly for those at high predicted risk |
| Y | Reeves (2024) [45] | CPRD Gold (158 GP practices) | UK (Yes) | 118,717 | 80-89 | 5 | Full (but variables adapted to fit CPRD and recalibrated the model) | Sex, age-80, age-80 squared, deprivation, stroke, diabetes, gait problems, major head injury, selective serotonin reuptake inhibitor use, tricyclic antidepressant use, other antidepressant use, NSAIDs (excluding aspirin), anticholinergic burden over last year (square-root), ever received social services, number of A&E visits in last year (square root), number of GP consultations involving third party in last year (square root), number of GP home visits in last year (square root), number of missed GP appointments in last year (square root), most recent BMI (square root), and most recent mean systolic blood pressure value/20 | None | All-cause (Primary Care record or ICD-10) | n=11,078 | Cox | 0.61 (0.60-0.61) | Not reported |
| Y | Walters (2016) [31] | THIN | UK (No) | 38,084 | 80-95 | 5 | Full | Age, age-squared, sex, calendar year, BMI, current anti-hypertensive use, SBP, lipid ratio, smoking status, history of alcohol problem, history of diabetes, history of stroke or TIA, history of AF, current depression/anti-depressants, current anxiety/anxiolytics, current aspirin use & current NSAID use (not aspirin) | None | All cause including AD, vascular dementia, and unspecified or mixed dementia (ICD-10) | n=1,923 | Cox  * Reports Uno’s c index | 0.56 (0.55-0.58) | Poor |
|  | **Model Validated**  eRADAR (Full model) |  |  |  |  |  |  |  |  |  |  |  |  |  |
| Y | Coley (2023) [35] | KPWA (2010-2020) | USA (No) | 129,315 | ≥65 | 1 & 1.5yrs | Full | Age, sex, congestive heart failure, cerebrovascular disease, diabetes (complex), diabetes (any), chronic pulmonary disease, hypothyroidism, renal failure, lymphoma, solid tumour without metastases, rheumatoid arthritis, weight loss, fluid and electrocyte disorders, blood loss anaemia, psychoses (& bipolar disorder), depression, TBI, tobacco use, AF, gait abnormality, underweight, obese, high blood pressure, ≥1 outpatient visit, ≥ emergency department visit, ≥1 physical therapy visit, ≥1 speech, language and learning visit, home health services & medication (antidepressants & sleep aids) | None | All-cause (notes, ICD-9, ICD-10 & medication data) | n=7,631 | ROC Curves | **1yr**  0.84 (0.84-0.85)  **1.5yrs**  0.83 (0.83-0.84) | NR |
| Y | Coley (2023) [35] | UCSF (2014-2019) | USA (No) | 13,444 | ≥65 | As above | As above | As above | None | As above | n=216 | As above | **1yr**  0.79 (0.76-0.82)  **1.5yrs** 0.78 (0.75 - 0.80) | NR |
|  | **Model Validated**  FDRS |  |  |  |  |  |  |  |  |  |  |  |  |  |
| N | Vonk (2021) [29] | AGES-RS | Iceland (No) | 5,343 | 66-98 | 5-6 & 10 | Full | Age, diabetes, stroke, TIA/Mini-stroke, cancer, BMI & marital status | None | All-cause (consensus) | 20.6% | Cox | **5-6yrs**  0.71 (0.69-0.73)  **10yrs**  0.70 (0.68-0.72) | **5-6yrs**  Good  **10yrs** Overestimated risk |
|  | **Model Validated**  FINDRISC |  |  |  |  |  |  |  |  |  |  |  |  |  |
| N | Fayosse (2020) [22] | Whitehall II | UK (No) | 7,553 | 39-63 | Mean 23.5 (4.0) | Full | Age, BMI, waist circumference, PA, daily fruit/vegetable intake, history of HBP medication, history of high blood glucose & family history of diabetes | None | All-cause (ICD-10) & hospital record | n=318 | Cox | 0.63 (0.60-0.66) | Poor |
|  | **Model Validated**  FRS |  |  |  |  |  |  |  |  |  |  |  |  |  |
| Y | Fayosse (2020) [22] | Whitehall II | UK (No) | 7,553 | 39-63 | Mean 23.5 (4.0) | Full | Age, smoking, diabetes, SPB, PB treatment, HDL & total cholesterol [Scores stratified by sex] | None | All-cause (ICD-10) & hospital record | n=318 | Cox | 0.72 (0.69-0.75) | Good |
| Y | Shang (2022) [38] | UKB | UK (No) | 471,485 | 38-73 | Median11.9 (IQR 11·2-12·6) | Full | As above | None | All-cause (ICD-9, ICD-10 and self-reported fields) | n=6,189 | Cox | 0.72 (0.71-0.72) | NR |
|  | **Model Validated**  GRS-19 |  |  |  |  |  |  |  |  |  |  |  |  |  |
| Y | Chouraki (2016) [19] | 3 City Study | France (No) | 3,079 | ≥65 | 5-8 | Full | GRS-19 + age, sex, education & APOE e4 status | None | DSM-IV | 7.7% | Cox | **5yrs**  0.77 (0.74-0.80)  **6yrs**  0.75 (0.72-0.77)  **7yrs**  0.75 (0.73-0.77)  **8yrs**  0.75 (0.73-0.78) | 7yrs good |
| Y | As above | ACT Study | USA (No) | 2,110 | ≥65 | As above | As above | As above | As above | As above | 19.8% | Cox | **5yrs**  0.82 (0.79-0.86)  **6yrs**  0.83 (0.79-0.86)  **7yrs**  0.79 (0.76-0.82)  **8yrs**  0.80 (0.76-0.82) |  |
| Y | As above | AGES-RS | Iceland (No) | 2,553 | ≥65 | As above | As above | As above | As above | As above | 11.0% | Cox | **5yrs**  0.79 (0.73-0.85)  **6yrs**  0.73 (0.68-0.78)  **7yrs**  0.70 (0.65-0.74)  **8yrs**  0.68 (0.64-0.72) |  |
| Y | As above | CHS | USA (No) | 1,998 | ≥65 | As above | As above | As above | As above | As above | 18.4% | Cox | **5yrs**  0.75 (0.71-0.79)  **6yrs**  0.75 (0.72-0.79)  **7yrs**  0.74 (0.71-0.78)  **8yrs**  0.72 (0.68-0.76) |  |
| N | As above | FHS | USA (No) | 1,757 | ≥65 | As above | Partial (missing SNP rs9271192) | GRS-19 (18 SNPs only) + age, sex, education & APOE e4 status | SNP rs9271192 | As above | 13.1% | Cox | **5yrs**  0.84 (0.81-0.87)  **6yrs**  0.82 (0.79-0.85)  **7yrs**  0.81 (0.79-0.83)  **8yrs**  0.80 (0.78-0.83) |  |
| Y | As above | ROSMAP | USA (No) | 1,262 | ≥65 | As above | Full | GRS-19 + age, sex, education & APOE e4 status | None | As above | 28.4% | Cox | **5yrs**  0.77 (0.74-0.80)  **6yrs**  0.77 (0.74-0.80)  **7yrs**  0.76 (0.74-0.79)  **8yrs**  0.77 (0.74-0.79) |  |
| N | As above | Rotterdam Study | Netherlands (No) | 3,334 | ≥65 | As above | Partial (missing SNP rs9271192) | GRS-19 (18 SNPs only) + age, sex, education & APOE e4 status | SNP rs9271192 | As above | 18.2% | Cox | **5yrs**  0.79 (0.76-0.82)  **6yrs**  0.79 (0.76-0.82)  **7yrs**  0.79 (0.76-0.82)  **8yrs**  0.78 (0.75-0.80) |  |
| N | As above | WHICAP | USA (No) | 594 | ≥65 | As above | Partial (missing SNP rs9271192) | GRS-19 (18 SNPs only) + age, sex, education & APOE e4 status | SNP rs9271192 | As above | 8.8% | Cox | **5yrs**  0.77 (0.68-0.87)  **6yrs**  0.81 (0.72-0.89)  **7yrs**  0.79 (0.71-0.88)  **8yrs**  0.79 (0.70-0.87) |  |
|  | **Model Validated**  HAAS models |  |  |  |  |  |  |  |  |  |  |  |  |  |
| N | Stephan (2023) [39] - NIA-Reagan model | AGES-RS (Men & women) | Iceland (No) | 4,733 | 33-80 | 26-4,499 days (Mean 2,484; SD 992) | Full | Age, hypertension treatment, smoking and APOE | None | All-cause, AD, VaD (consensus diagnosis) | 17% (All) | LR | **All-cause**  0.64 (0.61-0.66)  **AD**  0.63 (0.61-0.65)  **VaD**  0.61 (0.55-0.66) | **All-cause**  Poor  **AD**  Poor  **VaD**  Good |
| N | Stephan (2023) [39] – NP model | AGES-RS (Men & women) | Iceland (No) | 4,733 | As above | As above | As above | Age, smoking and APOE | As above | As above | As above | LR | **All-cause**  0.64 (0.62-0.66)  **AD**  0.64 (0.61-0.66)  **VaD**  0.60 (0.55-0.65) | **All-cause**  Poor  **AD**  Poor  **VaD**  Good |
| N | Stephan (2023) [39] – NFT model | AGES-RS (Men & women) | Iceland (No) | 4,347 | As above | As above | As above | Age, stroke, alcohol use and APOE | As above | As above | As above | LR | **All-cause**  0.65 (0.63-0.67)  **AD**  0.63 (0.61-0.66)  **VaD**  0.70 (0.64-0.76) | **All-cause**  Good  **AD**  Good  **VaD**  Poor |
| N | Stephan (2023) [39] – MVL model | AGES-RS (Men & women) | Iceland (No) | 4,708 | As above | As above | As above | Age, education, SBP, stroke and APOE | As above | As above | As above | LR | **All-cause**  0.58 (0.56-0.60)  **AD**  0.56 (0.53-0.58)  **VaD**  0.71 (0.65-0.77) | **All-cause**  Poor  **AD**  Poor  **VaD**  Poor |
| N | Stephan (2023) [39] – LI model | AGES-RS (Men & women) | Iceland (No) | 4,726 | As above | As above | As above | Age, cholesterol treatment, SBP & diabetes | As above | As above | As above | LR | **All-cause**  0.54 (0.52-0.56)  **AD**  0.53 (0.51-0.55)  **VaD**  0.58 (0.53-0.63) | **All-cause**  Poor  **AD**  Poor  **VaD**  Poor |
| N | Stephan (2023) [39] – MIXED PATHOLOGY model | AGES-RS (Men & women) | Iceland (No) | 4,733 | As above | As above | As above | Age, SBP & APOE | As above | As above | As above | LR | **All-cause**  0.63 (0.61-0.65)  **AD**  0.62 (0.60-0.65)  **VaD**  0.61(0.56-0.66) | **All-cause**  Poor  **AD**  Good  **VaD**  Good |
|  | **Model Validated**  LIBRA |  |  |  |  |  |  |  |  |  |  |  |  |  |
| N | Deckers (2020) [20] | CAIDE Study | Finland (No) | 1,024 | 65-79 | Mean 20.9 (4.9) | Partial (11 out of 12) | CHD, diabetes, hypercholesterolemia,  hypertension, depression, obesity, smoking, PA, renal disease, low‐to‐moderate alcohol use & Mediterranean diet | Cognitive activity | All-cause (DSM-IV & NINCDS-ADRDA) | n=84 | Cox | **Unadjusted**  0.65 (no diet)  **Adjusted (No diet) [Age, sex]** 0.67  **Unadjusted + diet**  0.68  **Adjusted + diet** 0.75 | NR |
| N | Huque (2023) [9] | RMAP | USA (No) | 843 | ≥60 | Median 5.0 (IQR: 1.0-2.0) | Partial (9 out of 12) | Obesity, diabetes, depression, smoking, PA, cognitive activity, alcohol intake, hypertension & CHD | Renal dysfunction, cholesterol & Mediterranean diet | All-cause & AD (Computer algorithm) | NR for analytical sample | ROC curves | 0.53 (0.48-0.57) | NR |
| N | Huque (2023) [9] | CHS-CS | USA (No) | 3,097 | ≥65 | Median 6.0 (IQR 0.2-7.7) | Partial (7 out of 12) | Diabetes, depression, smoking, alcohol intake, PA, CHD & kidney dysfunction | Cholesterol, obesity, hypertension, cognitive activity & Mediterranean diet | All-cause & AD (Consensus) | As above | ROC curves | 0.51 (0.48-0.54) | NR |
| N | Huque (2023) [9] | CHS-CS | USA (No) | 3,273 | ≥65 | Median 6.0 (IQR 0.2-7.7) | Partial (5 out of 12) | Depression, smoking, alcohol intake,  PA & CHD | Cholesterol, diabetes, obesity, hypertension, kidney dysfunction, cognitive activity & Mediterranean diet | All-cause & AD (Consensus) | As above | ROC curves | 0.51 (0.48-0.54) | NR |
| N | Huque (2023) [9] | HRS-ADAMS | USA (No) | 421 | ≥70 | Median (5.0 (IQR: 2.0-6.0) | Partial (7 out of 12) | Diabetes, depression,  smoking, cognitive activity, alcohol intake, hypertension & CHD | PA, renal dysfunction, cholesterol, obesity & Mediterranean diet | All-cause & AD (DSM-III-R & DSM-IV) | As above | ROC curves | 0.52 (0.45-0.59) | NR |
| N | Huque (2023) [9] | HRS-ADAMS | USA (No) | 432 | ≥70 | Median (5.0 (IQR: 2.0-6.0) | Partial (5 out of 12) | Diabetes, depression, smoking, cognitive activity & PA | Alcohol intake, hypertension, CHD, renal dysfunction, cholesterol, obesity & Mediterranean diet | All-cause & AD (DSM-III-R & DSM-IV) | As above | ROC curves | 0.53 (0.46-0.60) | NR |
| N | Schiepers (2018) [30] | Maastricht Ageing Study (MAAS) | Netherlands (No) | 949 | ≥50 | 12 | Partial (11 out of 12) | CHD, diabetes, hypercholesterolemia,  hypertension, depression, obesity, smoking, PA, renal disease, low‐to‐moderate alcohol use, & high cognitive activity | Mediterranean diet | All-cause (DSM-IV) | 6.4% (n=61) | Cox | 0.60 (0.53-0.67) | NR |
| N | Vos (2017) [30] | DESCRIPTA | Sweden, Italy, Netherlands & France | 9,387 | ≥55 | 16 | Partial (9 of 12) | CHD, diabetes, hypercholesterolemia,  hypertension, depression, obesity, smoking, PA & low-to‐moderate alcohol use | Renal dysfunction, cognitive activity & Mediterranean diet | Dementia (DSM-III-R) or impairment in multiple cognitive domains (for LASA) | 16.8 per 1000 person-years | Cox | **55-69yrs**  0.57 (SE=0.03)  **70-79yrs**  0.50 (SE=0.01)  **80-97yrs**  0.54 (SE=0.02) | NR |
|  | **Model Validated**  LIBRA-Modified |  |  |  |  |  |  |  |  |  |  |  |  |  |
| N | Huque (2023) [9] | RMAP | USA (No) | 843 | ≥60 | Median 5.0 (IQR: 1.0-2.0) | Partial (12 out of 15) | Age, sex, education, obesity, diabetes, depression, smoking,  PA, cognitive activity, alcohol intake, hypertension & CHD | Renal dysfunction, cholesterol & Mediterranean diet | All-cause & AD (Computer algorithm) | NR for analytical sample | ROC curves | 0.65 (0.61-0.69) | NR |
| N | Huque (2023) [9] | CHS-CS | USA (No) | 3,097 | ≥65 | Median 6.0 (IQR 0.2-7.7) | Partial (10 out of 15) | Age, sex, education, diabetes, depression, smoking, alcohol intake, PA, CHD & kidney dysfunction | Cholesterol, obesity, hypertension, cognitive activity & Mediterranean diet | All-cause & AD (Consensus) | NR for analytical sample | ROC curves | 0.70 (0.68-0.73) | NR |
| N | Huque (2023) [9] | CHS-CS | USA (No) | 3,273 | ≥65 | Median 6.0 (IQR 0.2-7.7) | Partial (8 out of 15) | Age, sex, education, depression, smoking, alcohol intake, PA & CHD | Cholesterol, diabetes, kidney dysfunction, obesity, hypertension, cognitive activity & Mediterranean diet | All-cause & AD (Consensus) | NR for analytical sample | ROC curves | 0.70 (0.68-0.73) | NR |
| N | Huque (2023) [9] | HRS-ADAMS | USA (No) | 421 | ≥70 | Median (5.0 (IQR: 2.0-6.0) | Partial (10 out of 15) | Age, sex, education, diabetes, depression, smoking, cognitive activity, alcohol intake, hypertension & CHD | PA, renal dysfunction, cholesterol, obesity & Mediterranean diet | All-cause & AD (DSM-III-R & DSM-IV) | NR | ROC curves | 0.67 (0.61-0.74) | NR |
| N | Huque (2023) [9] | HRS-ADAMS | USA (No) | 432 | ≥70 | Median (5.0 (IQR: 2.0-6.0) | Partial (8 out of 15) | Age, sex, education, diabetes, depression, smoking & cognitive activity | Alcohol intake, hypertension, CHD, PA, renal dysfunction, cholesterol, obesity & Mediterranean diet | All-cause & AD (DSM-III-R & DSM-IV) | NR | ROC curves | 0.67 (0.61-0.74) | NR |
|  | **Model Validated**  LLDRI |  |  |  |  |  |  |  |  |  |  |  |  |  |
| N | Vonk (2021) [29] | AGES-RS | Iceland (No) | 5,343 | 66-98 | 5-6 | Full | Age, coronary bypass surgery, APOE, white matter disease, ventricular enlargement, carotid intima-media thickness, BMI, MMSE/3MS, DSST total correct, difficulty to dress & alcohol | None | All-cause (consensus) | 20.6% | LR | 0.80 (0.78-0.82) | Overestimated risk |
|  | **Model Validated** MADeN |  |  |  |  |  |  |  |  |  |  |  |  |  |
| N | Vonk (2021) [29] | AGES-RS | Iceland (No) | 5,343 | 66-98 | 10 | Full | Age, sex, education, diabetes, depressive symptoms, pain walking/standing, difficulty to walk, IADL score, social leisure activity & not having friends | None | All-cause (consensus) | 20.6% | Fine-Gray | 0.72 (0.71-0 .74) | Overestimated risk |
|  | **Model Validated**  Nori-ADRD Score |  |  |  |  |  |  |  |  |  |  |  |  |  |
| Y | John (2022) [25] | MDCR | USA (NR) | 10M | NR | 5 | Full (50) | Demographic measures, medical diagnosis, prescription medication & medical procedures. **See Appendix 3 above for the full list of conditions.** | None | NR | NR | pROC R-package | 0.66 (0.66–0.67) | Calibration E_avg_=0.164 |
| Y | John (2022) [25] | IQGER | Germany (NR) | 30M | NR | 5 | Full (50) | As above | As above | NR | NR | As above | 0.67 (0.66–0.68) | Calibration E_avg_=0.142 |
| Y | John (2022) [25] | OPSES | USA (NR) | 85M | NR | 5 | Full (50) | As above | As above | NR | NR | As above | 0.67 (0.66–0.68) | Calibration E_avg_=0.258 |
| Y | John (2022) [25] | OPEHR | USA (NR) | 94M | NR | 5 | Full (50) | As above | As above | NR | NR | As above | 0.62 (0.62–0.63) | Calibration E_avg_=170 |
| Y | John (2022) [25] | CPRD | UK (NR) | 13M | NR | 5 | Full (50) | As above | As above | NR | NR | As above | 0.68 (0.67–0.69) | Calibration E_avg_=0.198 |
| Y | John (2022) [25] | IPCI | Netherlands (NR) | 2.5M | NR | 5 | Full (50) | As above | As above | NR | NR | As above | 0.64 (0.62–0.67) | Calibration E_avg_=0.790 |
| Y | John (2022) [25] | IMRD | UK (NR) | 18M | NR | 5 | Full (50) | As above | As above | NR | NR | As above | 0.68 (0.68–0.69 | Calibration E_avg_=0.190 |
|  | **Model Validated**  OPERH |  |  |  |  |  |  |  |  |  |  |  |  |  |
| N | John (2024) [44] | MDCR  IQGER  OPSES  IPCI | USA (NR)  Germany (NR)  USA (NR)  Netherlands (NR) | MDCR: 999,480  IQGER: 946,900  OPSES: 999,439  IPCI: 186,767 | 55-84 | 5 | Full | Age, sex, history of acetaminophen exposures, antiepileptic medication exposures, seizures, anemia, cancer, major depressive disorder, chronic obstructive pulmonary disease, heart failure, chronic kidney disease, hyperlipidemia, hypertension, obesity, alcoholism, anxiety, Type 2 diabetes, smoking, & urinary tract infections | None | All-cause (Concept codes from OMOP CDM) | MDCR: 44,800 (4.5%)  IQGER: 37,643 (4.0%)  OPSES: 47,764 (4.8%)  IPCI: 3,094 (1.7%) | LR (Regularization type: Broken Adaptive Ridge) | MDCR:0.72  IQGER: 0.77  OPSES: 0.80  IPCI: 0.83 | MDCR: E_ave_=0.0087  IQGER: E_ave_=0.0071  OPSES: E_ave_=0.0083  IPCI: E_ave_=0.0104 |
|  | **Model Validated**  Plasma phospholipids (10-metabolite panel) |  |  |  |  |  |  |  |  |  |  |  |  |  |
| Y | Casanova (2016) [18] | BLSA | USA (No) | 192 | Mean 77.2 (SD 6.6) | Mean 4.8 (1.2) | Full | 10-metabolite panel in plasma including Propionyl AC (C3), LysoPC a C18:2, PC aa C36:6, C16:1-OH, PC aa C38:0, PC aa C38:6, PC aa C40:1, PC aa C40:2, PC aa C40:6, and PC ae C40:6 | None | AD | n=93 | LR | 0.64 | NR |
| Y | Casanova (2016) [18] | AGES-RS | Iceland (No) | 200 | Mean 78.2 (SD 4.4) | Mean 2.6 (0.1) | As above | As above | None | AD | n=100 | As above | 0.40 | NR |
|  | **Model Validated**  **RADaR** |  |  |  |  |  |  |  |  |  |  |  |  |  |
| Y | Capuano (2022) [17] | ROS | USA (No) | 1,299 | ≥65 | Median 10 | Full | Age, memory complaints, difficulty with finances, orientation (month & room), & 3-word delayed recall | None | All-cause (NINDS & ADRDA) | NR | Fine-Gray | **3-yrs**  0.82 (0.78-0.86) | NR |
| Y | Capuano (2022) [17] | MARS | USA (No) | 679 | ≥65 | Median 8 | Full | As above | None | All-cause (NINDS & ADRDA) | NR | Fine-Gray | **3-yrs**  0.85 (0.80-0.90) | NR |
|  | **Model Validated**  RxDx-Dementia Risk Index |  |  |  |  |  |  |  |  |  |  |  |  |  |
| Y | John (2022) [25] | MDCR | USA (No) | 10M | NR | 5 | Full | 26 including demographic measures, medical diagnoses & medical procedures (specific variables NR) | None | NR | NR | pROC R-package | 0.69 (0.69–0.70) | Not possible to calculate |
| Y | John (2022) [25] | IQGE | Germany (No) | 30M | NR | 5 | As above | As above | As above | NR | NR | pROC R-package | 0.72 (0.71–0.72) | As above |
| Y | John (2022) [25] | OPSES | USA (No) | 85M | NR | 5 | As above | As above | As above | NR | NR | pROC R-package | 0.71 (0.70–0.71) | As above |
| Y | John (2022) [25] | OPEHR | USA (No) | 94M | NR | 5 | As above | As above | As above | NR | NR | pROC R-package | 0.73 (0.73–0.73) | As above |
| Y | John (2022) [25] | CPRD | UK (No) | 13M | NR | 5 | As above | As above | As above | NR | NR | pROC R-package | 0.79 (0.78–0.80) | As above |
| Y | John (2022) [25] | IPCI | Netherlands (No) | 2.5M | NR | 5 | As above | As above | As above | NR | NR | pROC R-package | 0.78 (0.76–0.80) | As above |
| Y | John (2022) [25] | IMRD | UK (No) | 18M | NR | 5 | As above | As above | As above | NR | NR | pROC R-package | 0.79 (0.78–0.80) | As above |
|  | **Model Validated**  SCORE |  |  |  |  |  |  |  |  |  |  |  |  |  |
| N | Zheng (2023) [41] | UKB | UK (No) | 429,033 | 38-79 | Median 12.8 | Full | Age, sex, smoking, SBP, diabetes, total cholesterol & HDL cholesterol + interactions between risk factors & age | None | All-cause, AD & VaD (ICD-9 & ICD-10) | **All-cause** n=6,477  **AD** n=2,726  **VaD** n=1,439 | Cox | **All-cause**  0.74 (0.73-0.74)  **AD**  0.74 (0.73-0.75)  **VaD**  0.77 (0.76-0.78) | NR |
|  | **Model Validated**  SCORE2/SCORE2-OP |  |  |  |  |  |  |  |  |  |  |  |  |  |
| N | Zheng (2023) [41] | UKB | UK (No) | 429,033 | 38-79 | Median 12.8 | Full | Age, sex, smoking, SBP, diabetes, total cholesterol & HDL cholesterol + interactions between risk factors & age | None | All-cause (n=6,477), AD (n=2,726) & VD (1,439) IICD-9 & ICD-10) | **All-cause** n=6,477  **AD**  n=2,726  **VaD** n=1,439 | Cox | **All-cause**  0.75 (0.75-0.76)  **AD**  0.75 (0.74-0.76)  **VaD**  0.80 (0.79-0.81) | NR |
|  | **Model Validated**  Tierney (2005) |  |  |  |  |  |  |  |  |  |  |  |  |  |
| N | Vonk (2021) [29] | AGES-RS | Iceland (No) | 5,343 | 66-98 | 10 | Full | Age, education & delayed memory recall | None | AD (consensus) | 10.4% | LR | 0.76 (0.74-0.78) | Overestimated risk |
|  | **Model Validated**  Tierney (2010) |  |  |  |  |  |  |  |  |  |  |  |  |  |
| N | Vonk (2021) [29] | AGES-RS | Iceland (No) | 5,343 | 66-98 | 10 | Full | Age, sex, education, Delayed Memory Recall & DSST total score | None | All-cause (consensus) | 20.6% | LR | 0.77 (0.76-0.79) | Underestimated risk |
|  | **Model Validated**  Verhaaren_2 (2013) |  |  |  |  |  |  |  |  |  |  |  |  |  |
| N | Vonk (2021) [29] | AGES-RS | Iceland (No) | 5,343 | 66-98 | 10 | Full (2 factor model) | Age & sex | None | AD (consensus) | 10.4% | Cox | 0.70 (0.68-0.72) | Overestimated risk |
|  | **Model Validated**  Verhaaren_3 (2013) [76] |  |  |  |  |  |  |  |  |  |  |  |  |  |
| N | Vonk (2021) [29] | AGES-RS | Iceland (No) | 5,343 | 66-98 | 10 | Full (3 factor model) | Age, sex & APOE | None | AD (consensus) | 10.4% | Cox | 0.73 (0.71-0.75) | Overestimated risk |
|  | **Model Validated** UKBDRS |  |  |  |  |  |  |  |  |  |  |  |  |  |
| N | Anatürk (2023) [33] | Whitehall II | UK (No) | 2,934 | Median 57 (IQR 10) | 17 | Full | Age, sex, education, parental history of dementia, material deprivation, diabetes, stroke, depression, hypertension, high cholesterol & household occupancy | None | All-cause (self-report & hospital in-patient records: ICD-9 & ICD-10) | 3.2% (n=93) | Fine Gray | 0.77 (0.72-0.81) | Not undertaken due to insufficient data |
|  | **Model Validated** UKBDRS+APOE |  |  |  |  |  |  |  |  |  |  |  |  |  |
| N | Anatürk (2023) [33] | Whitehall II | UK (No) | 2,315 | Median 57 (IQR 10) | 17 | Full | Age, sex, education, parental history of dementia, material deprivation, diabetes, stroke, depression, hypertension, high cholesterol & household occupancy + APOE | None | All-cause (self-report & hospital in-patient records: ICD-9 & ICD-10) | 3.2% (n=93) | Fine Gray | 0.80 (0.75-0.85) | Not undertaken due to insufficient data |
|  | **Model Validated**  Yang (2022) Models A to D |  |  |  |  |  |  |  |  |  |  |  |  |  |
| N | Yang (2022) [40] | ROS | USA (No) | 1,103 | NR | 3 & 5 | Full Model A | **Model A** 55 non-cognitive covariates | None | AD (NIA-AA criteria) | NR | Cox | **3yrs** 0.80  **5yrs** 0.79 | NR |
| N | Yang (2022) [40] | ROS | USA (No) | As above | As above | As above | Full Model B | **Model B** 55 non-cognitive covariates + MMSE | As above | As above | As above | As above | **3yrs** 0.84  **5yrs** 0.82 | NR |
| N | Yang (2022) [40] | ROS | USA (No) | As above | As above | As above | Full Model C | **Model C** 55 non-cognitive covariates+ MMSE+ Composite cognition | As above | As above | As above | As above | **3yrs** 0.92  **5yrs** 0.88 | NR |
| N | Yang (2022) [40] | ROS | USA (No) | As above | As above | 1, 3 & | Full Model D | **Model D** Composite cognition score only | As above | As above | As above | As above | **1yr** 0.95  **3yrs** 0.91  **5yrs** 0.86 | NR |

# Figure S2 Forest-Plots describing the performance (i.e., c-statistic) of fully externally validated risk models for predicting all-cause dementia


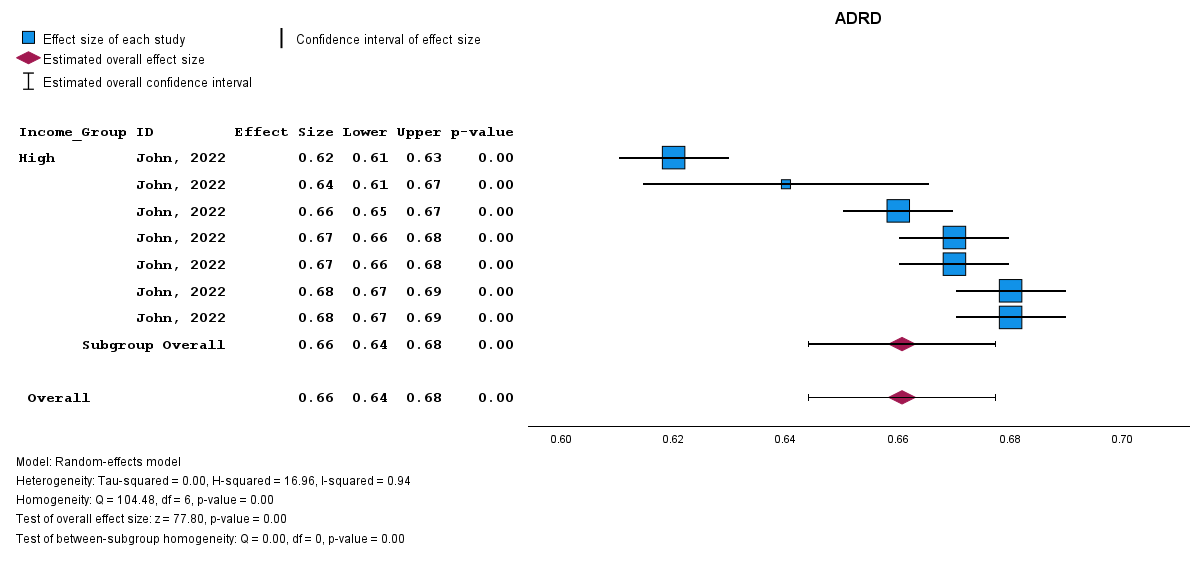


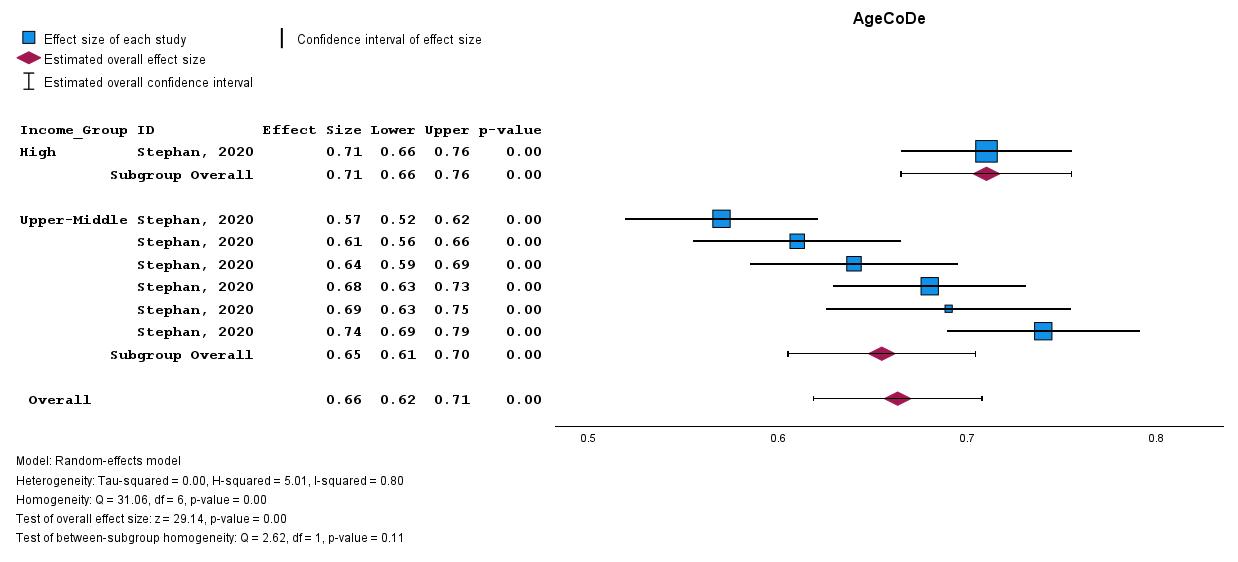


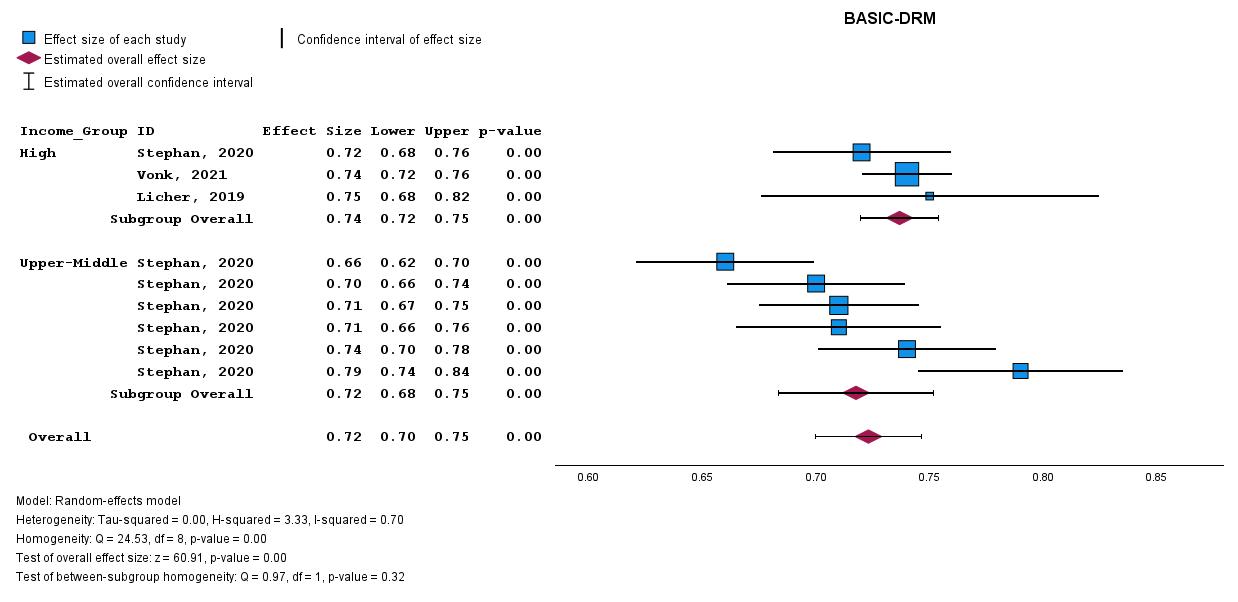


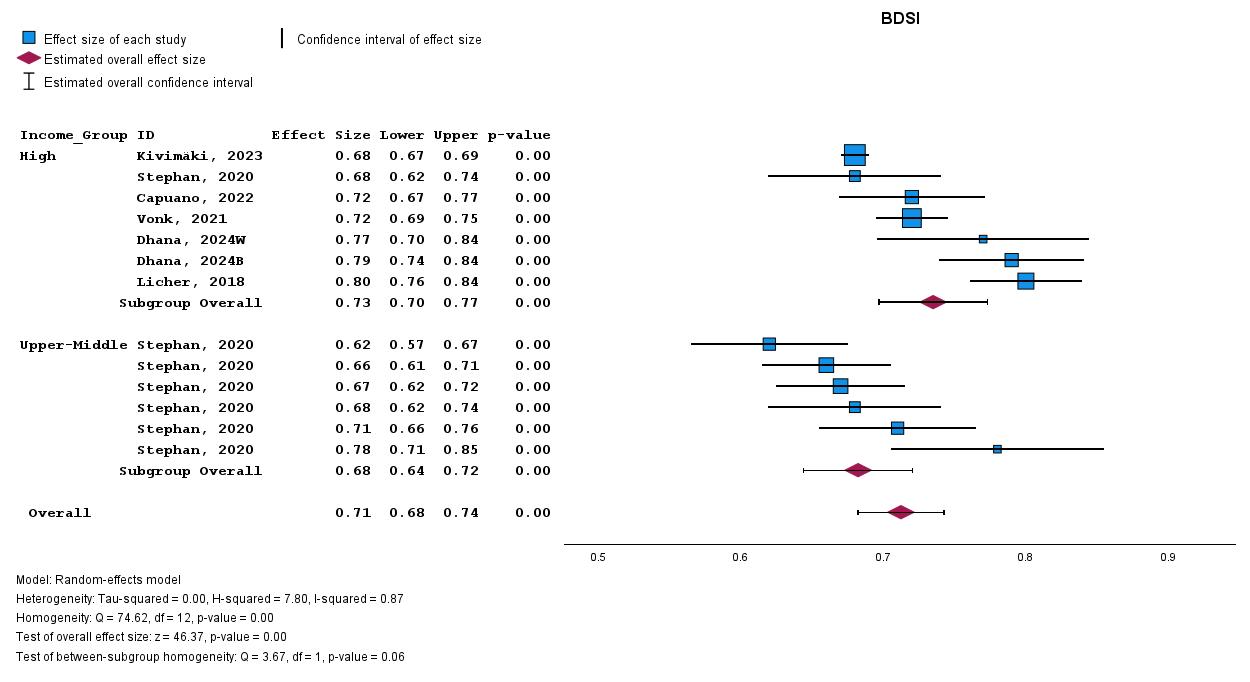


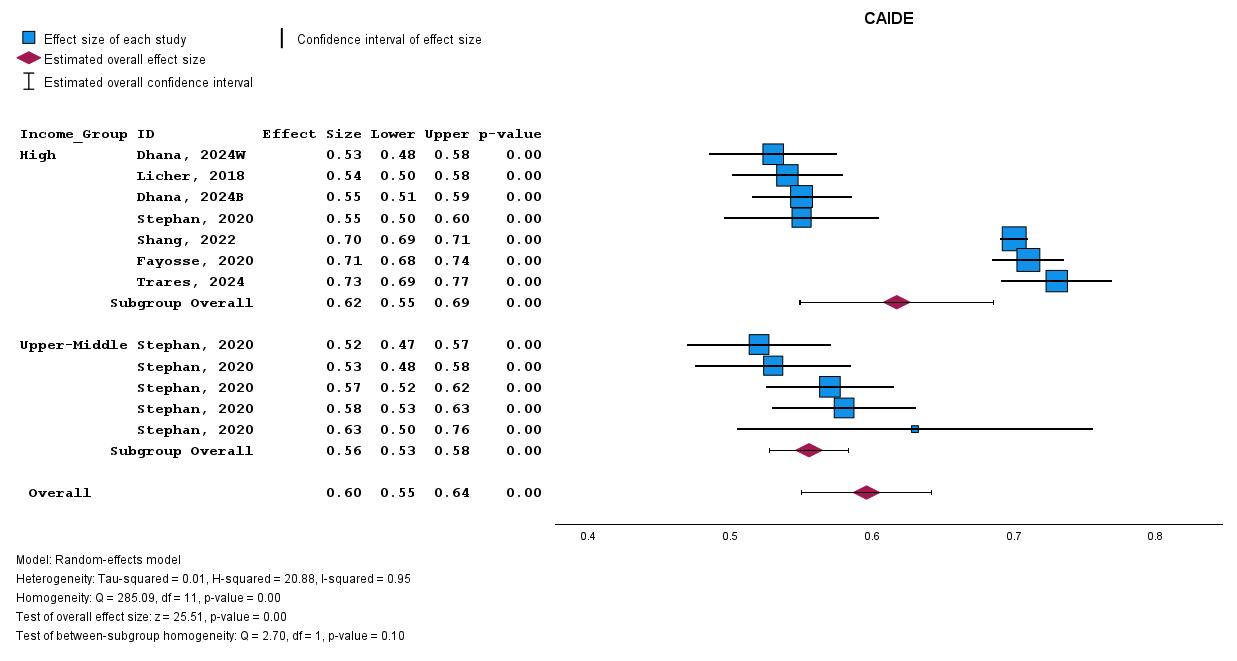


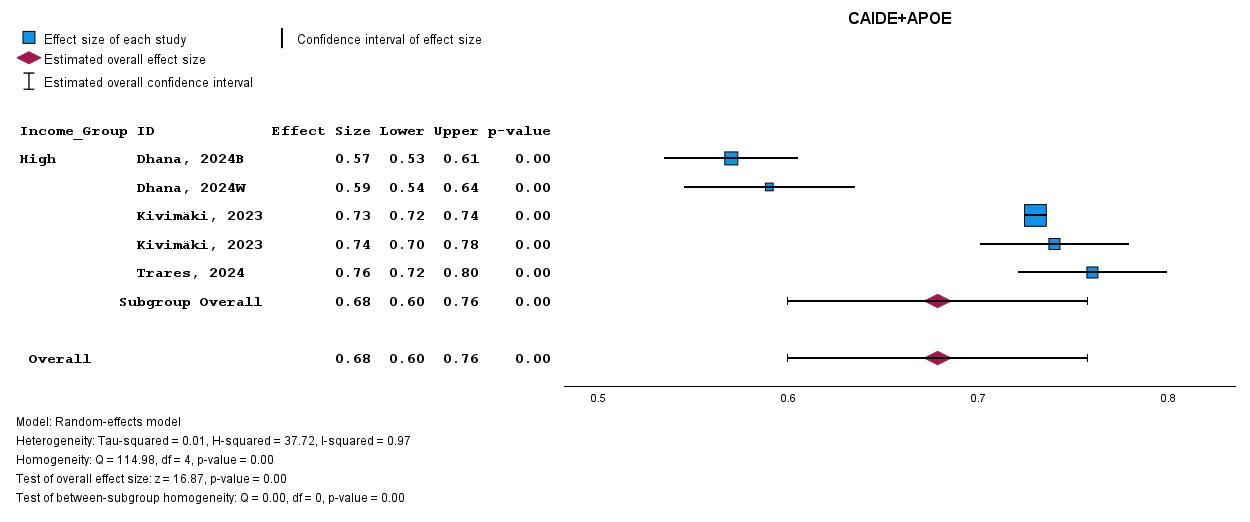


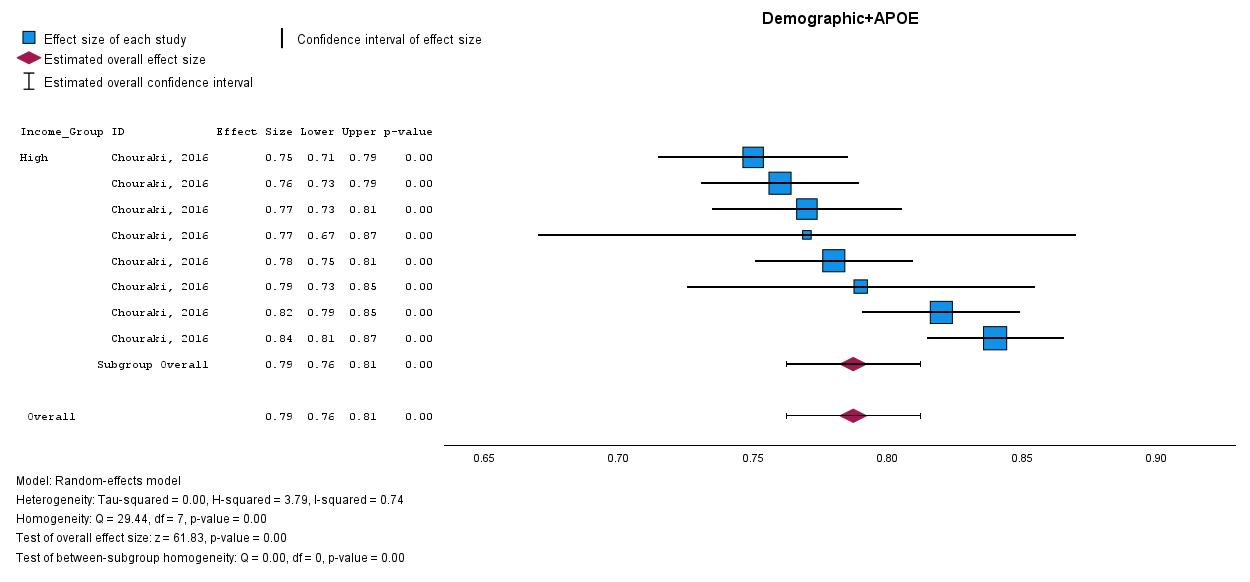


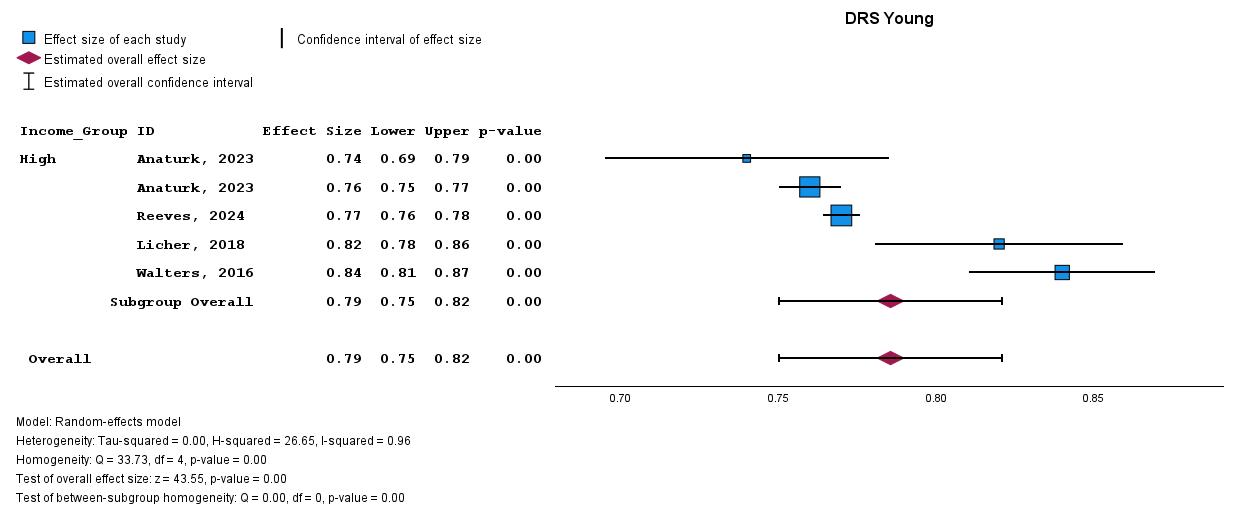


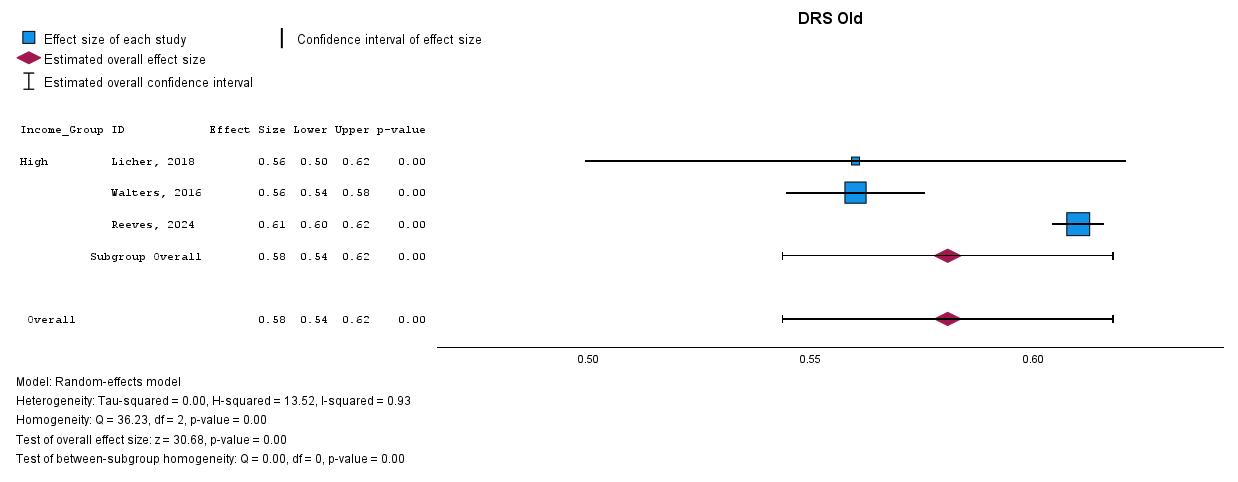


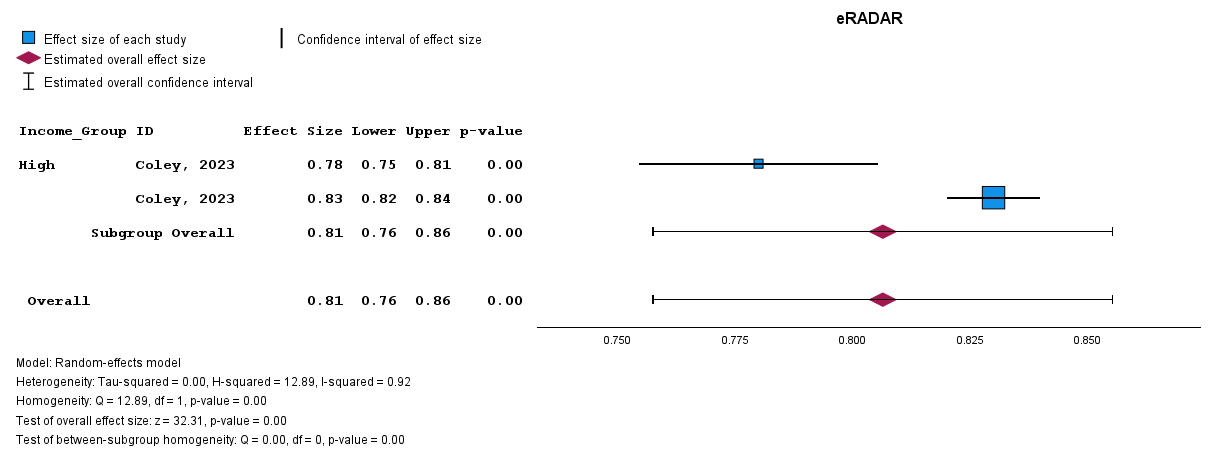


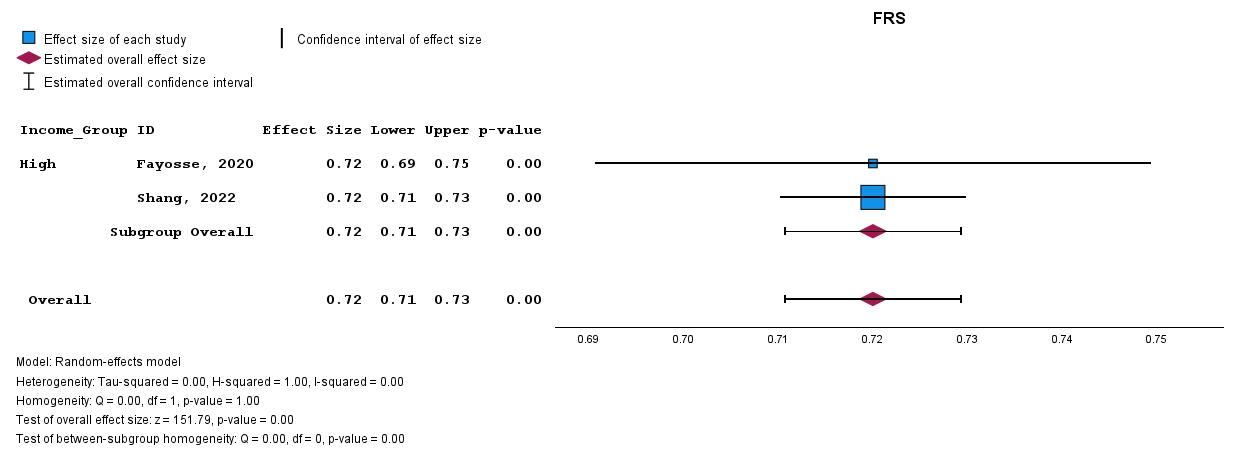


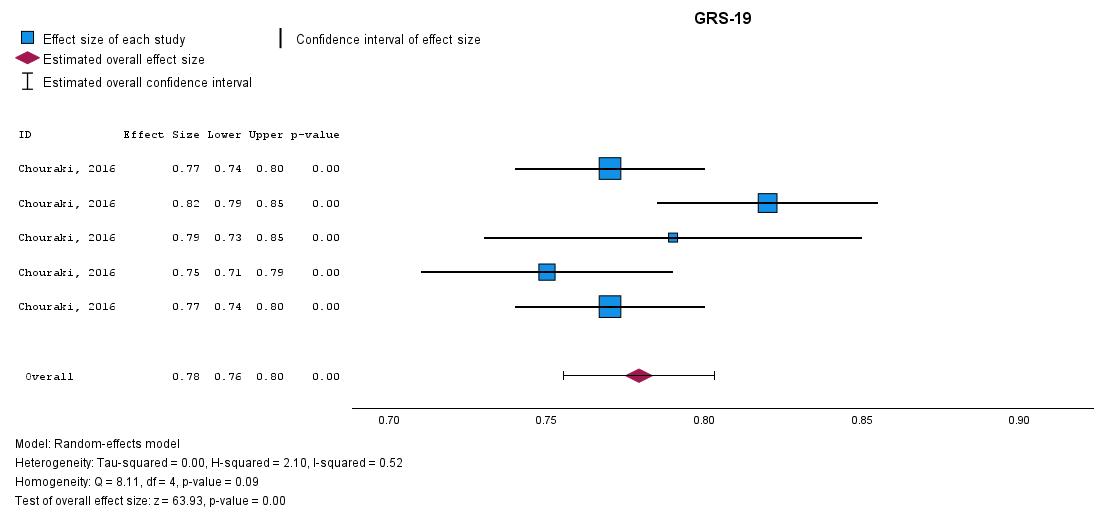


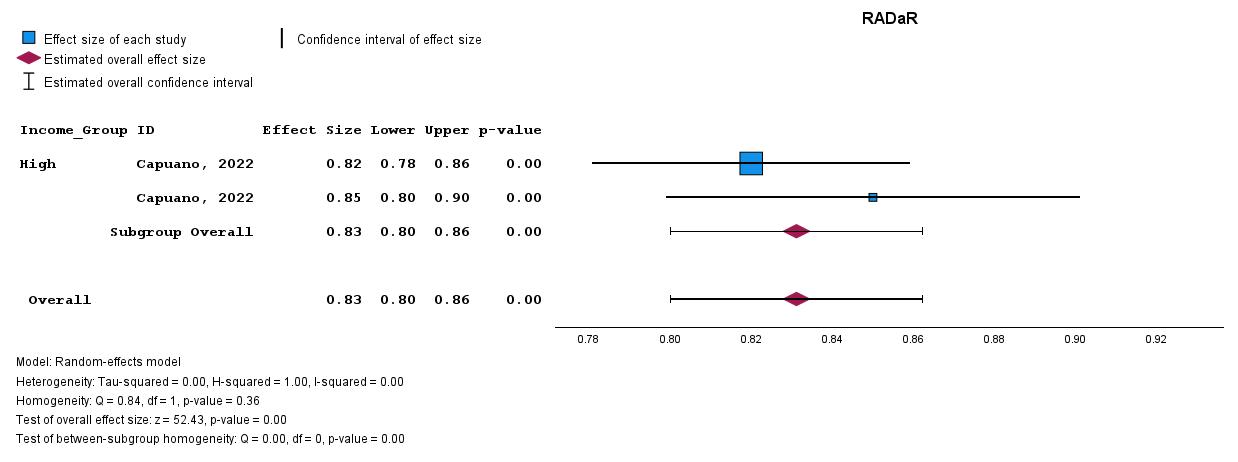


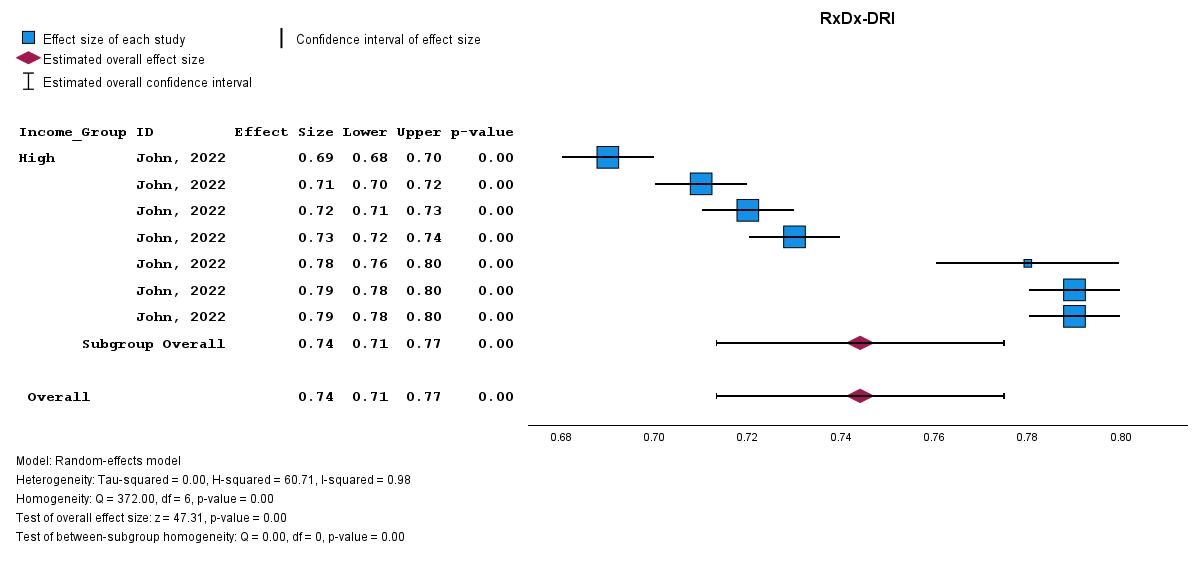


Acronyms: AD, Alzheimer’s Disease; N, Number of validations; 95% CI, 95% Confidence Intervals; AgeCoDe, Ageing, Cognition, and Dementia; ADRD, Alzheimer's Disease Related Dementias; BDSI, Brief Dementia Screening Index; CAIDE, Cardiovascular Risk Factors, Aging, and Incidence of Dementia; DRM, Dementia Risk Model; DRS, Dementia Risk Score; eRADaR, electronic Risk of Alzheimer's and Dementia Assessment Rule; GRS-19, Genetic Risk Score; RxDx-DRI, disease conditions (Dx), prescription drugs (Rx) Dementia Risk Index

#

# Figure S3 Forest-Plots describing the performance (i.e., c-statistic) of fully externally validated risk models for predicting Alzheimer’s disease and Vascular Dementia

**Alzheimer’s Disease**


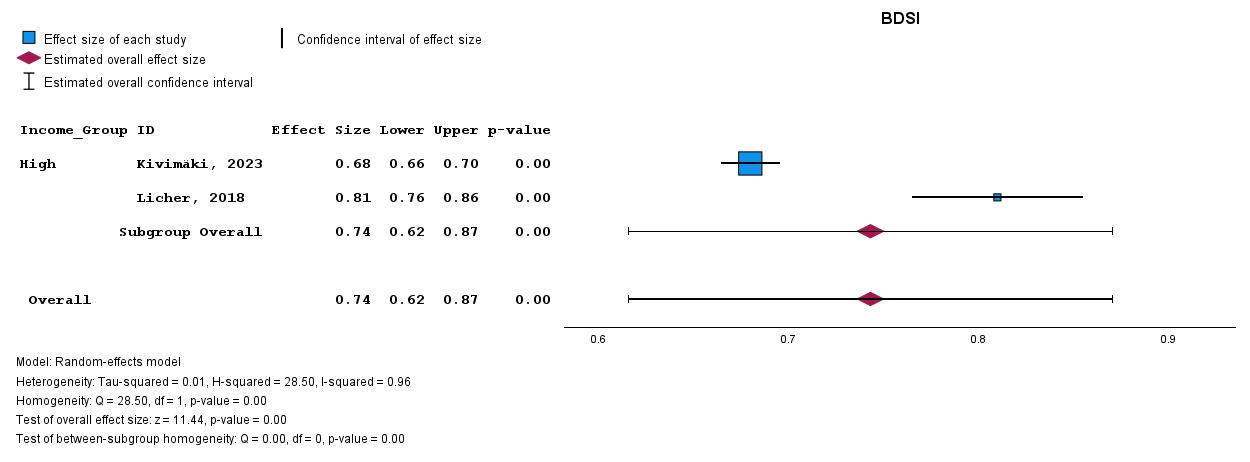


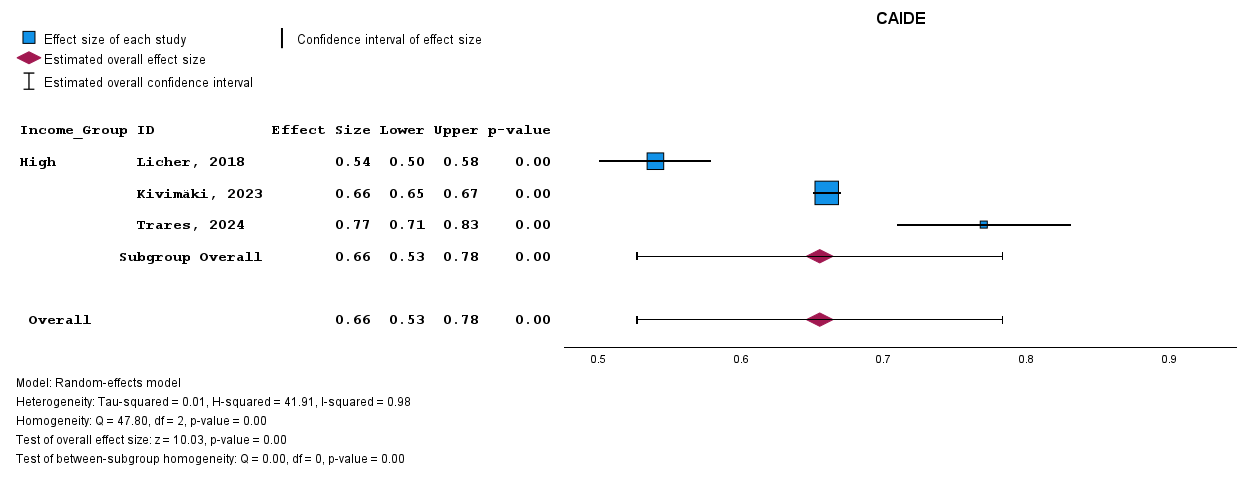


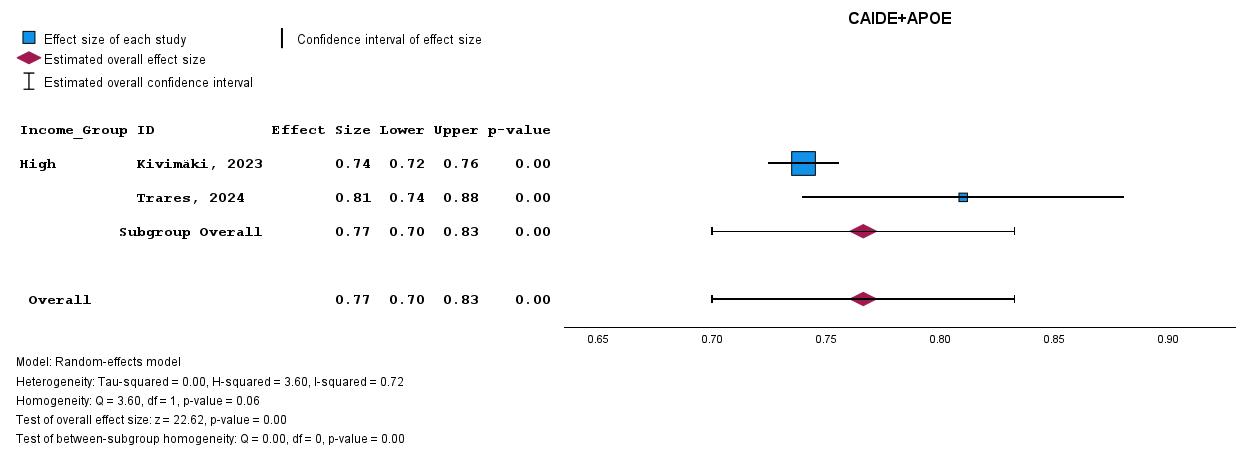


**Vascular Dementia**


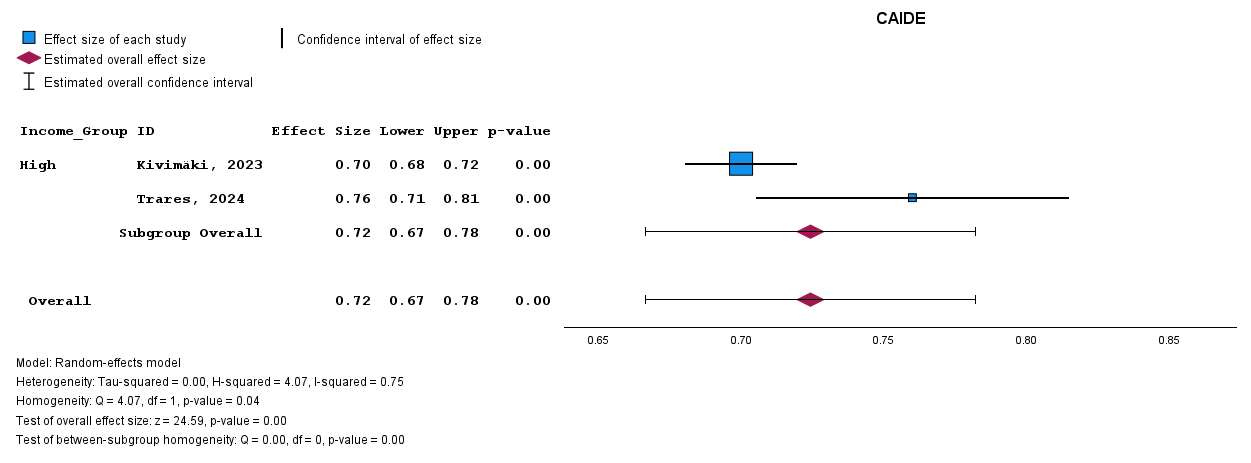


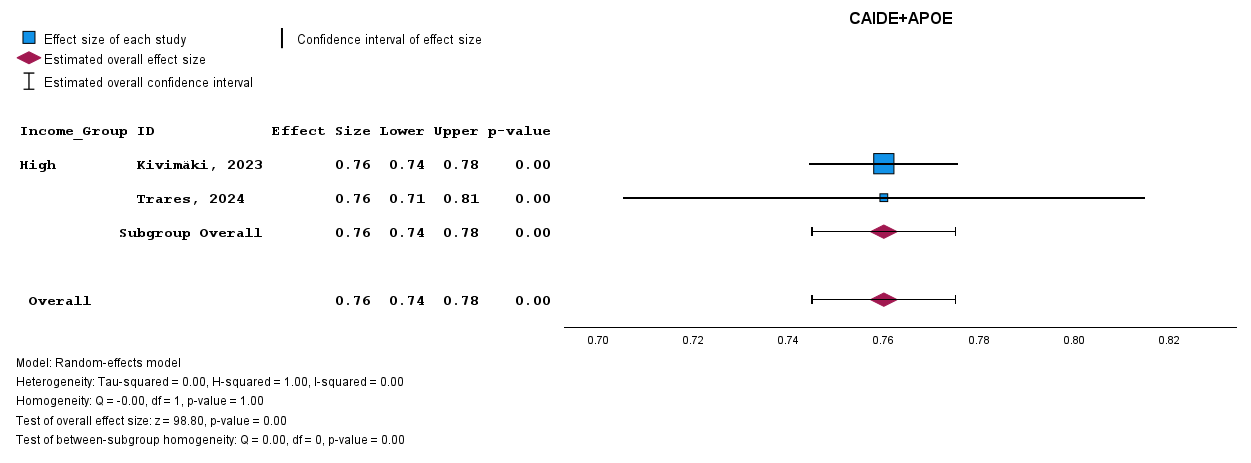


Note: Error bars are 95% Confidence Intervals.

Acronyms: BDSI, Brief Dementia Screening Index; CAIDE, Cardiovascular Risk Factors, Aging, and Incidence of Dementia.

# Figure S4 Publication Bias

Figures show the evaluation of publication bias in fully validated models to predict all cause dementia. Funnel plots are shown to describe the publication bias of the meta-analyses. The publication bias was statistically assessed using Egger’s regression test. Regression estimates were calculated for models that included a minimum of 3 studies or more.


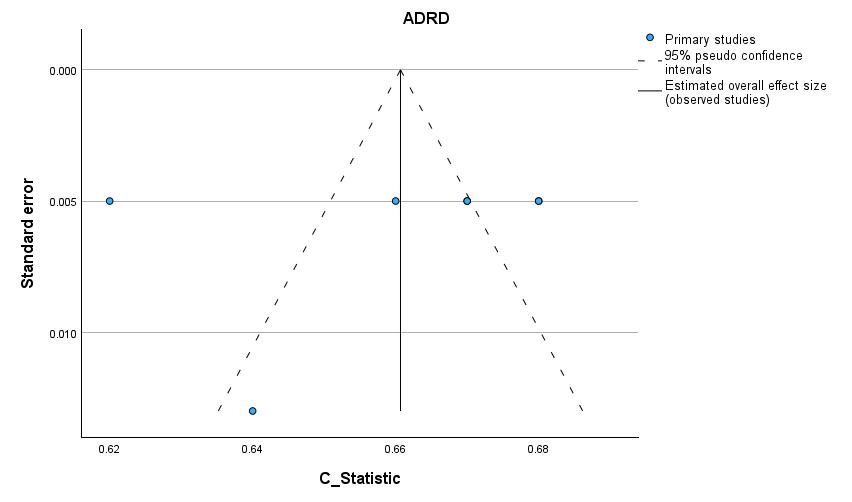


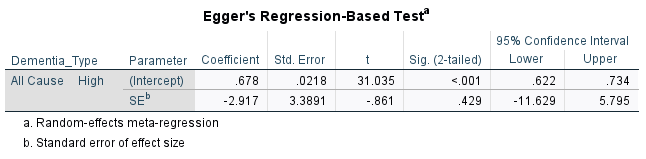


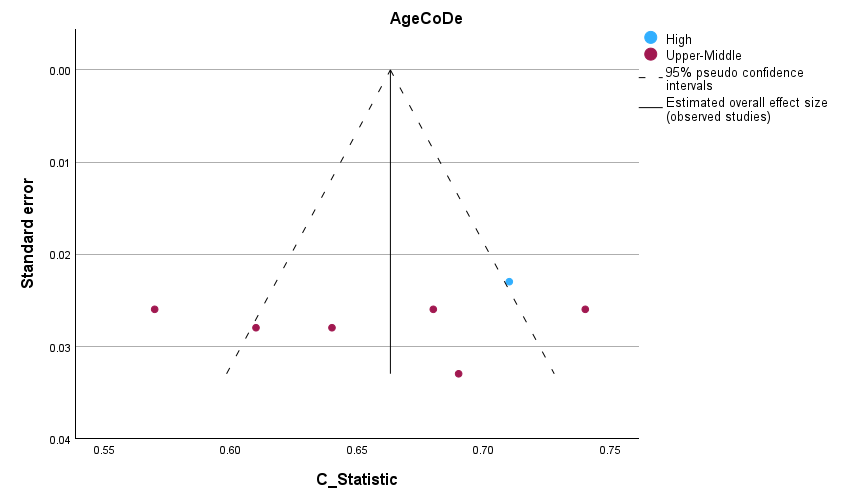


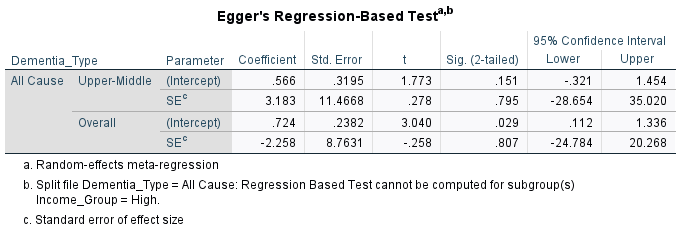


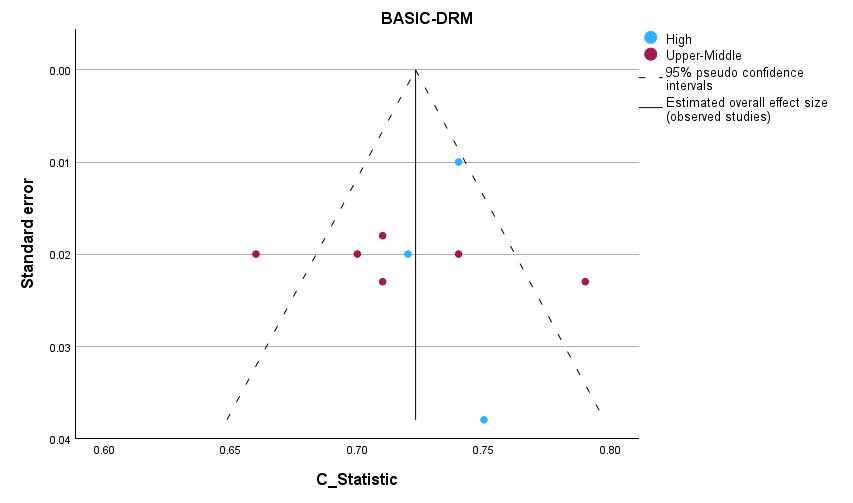


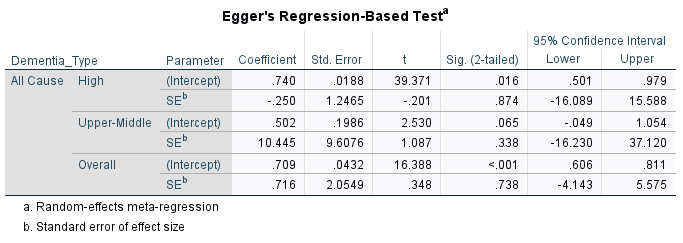


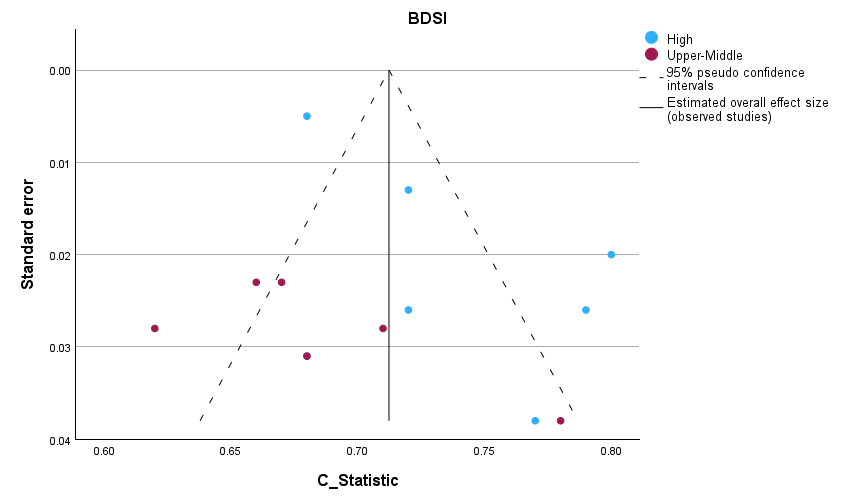


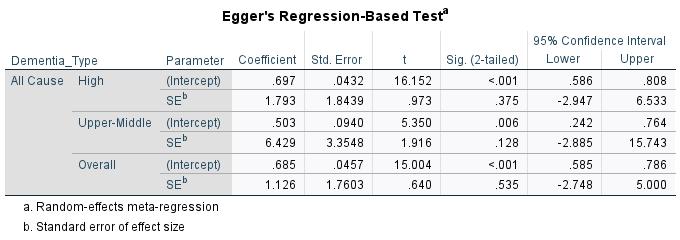


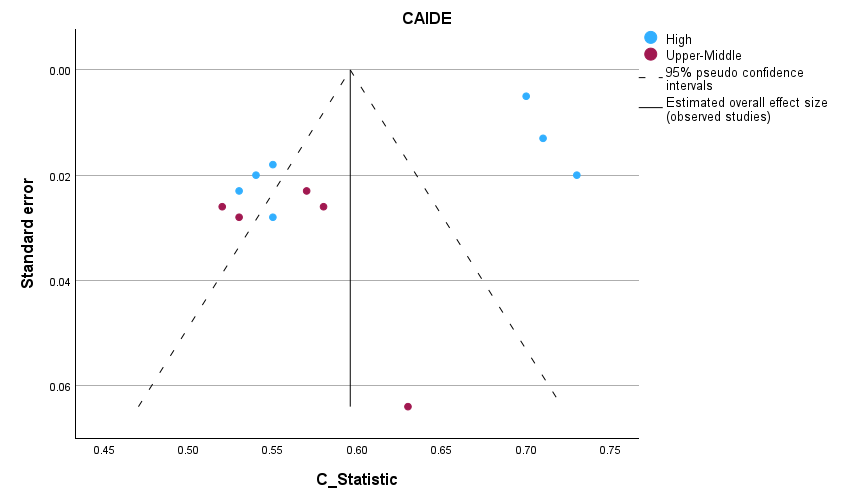


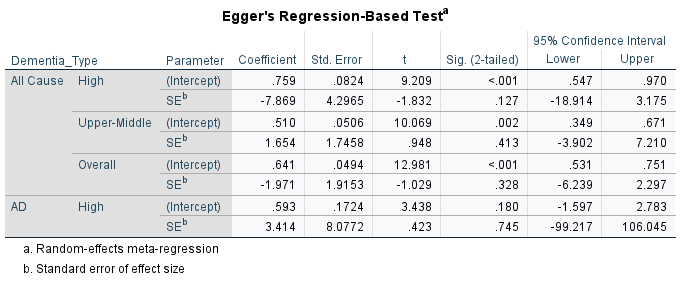


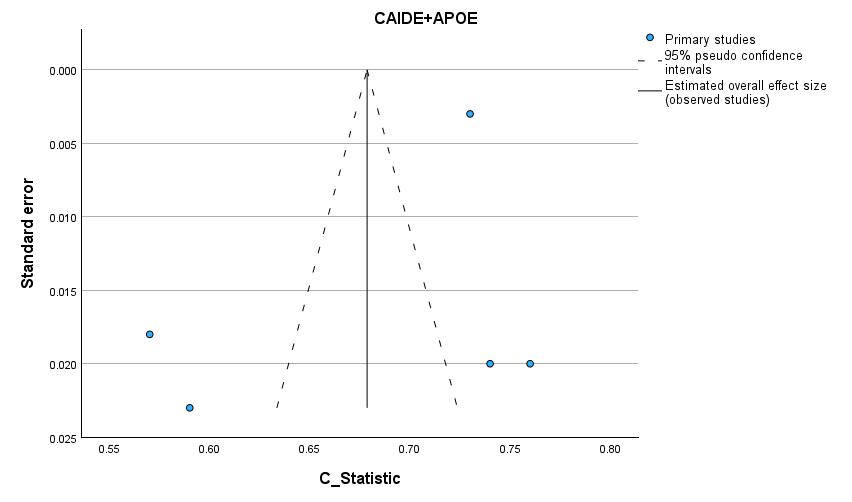


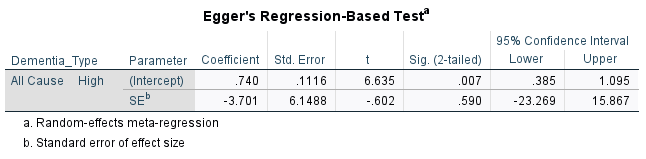


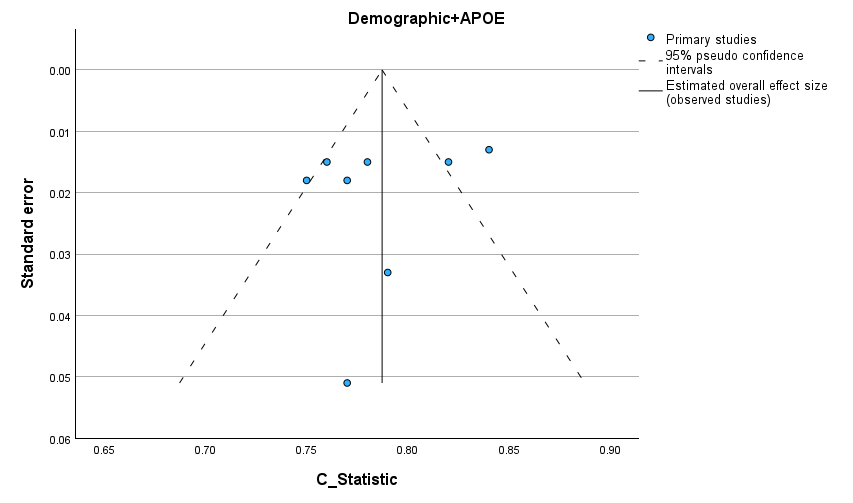


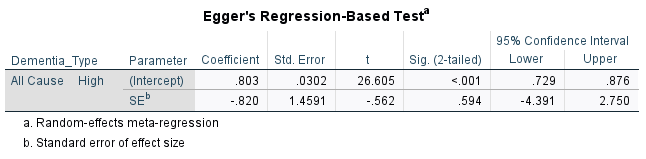


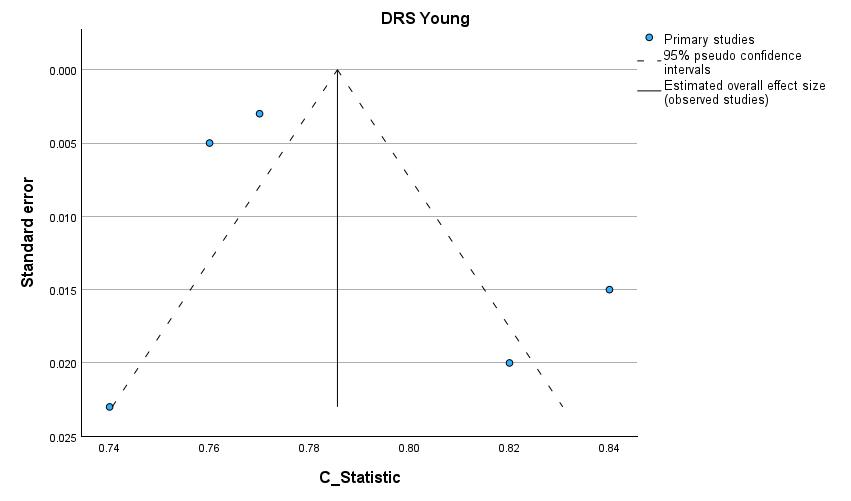


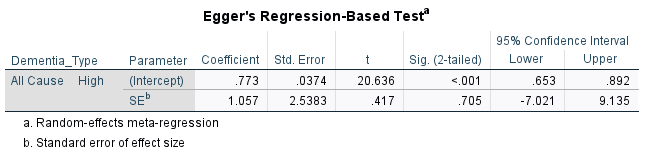


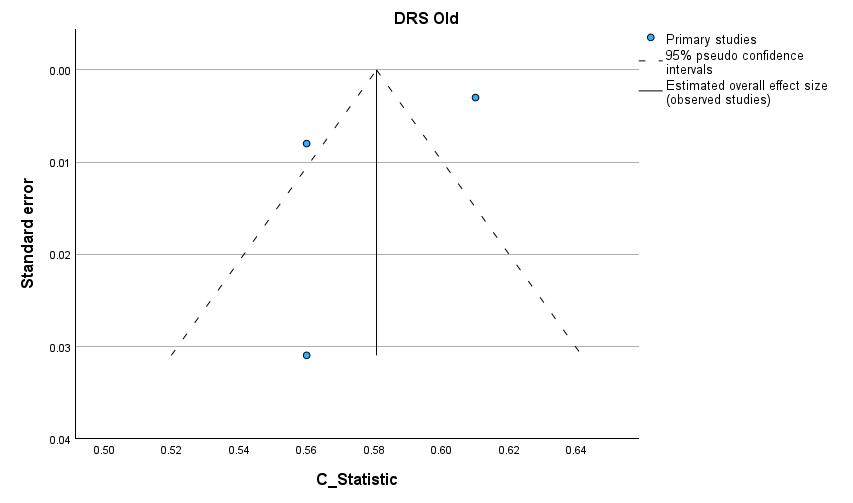


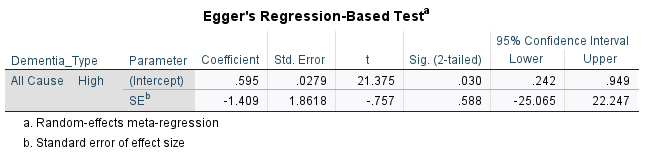


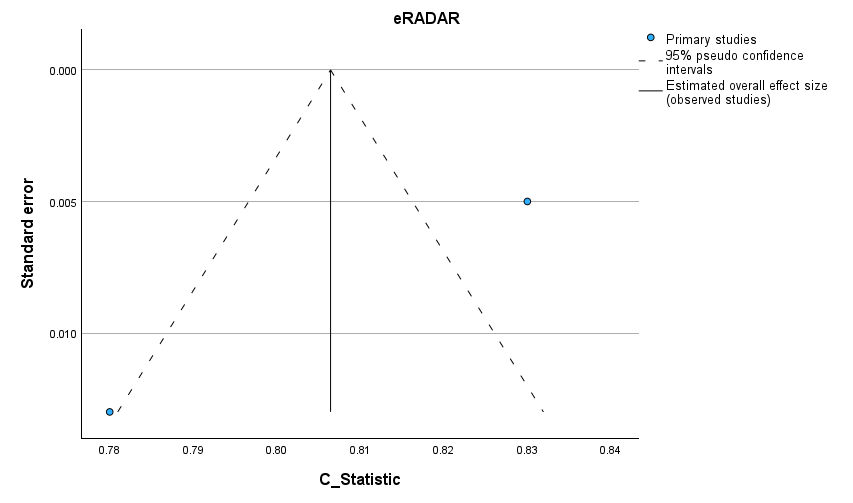


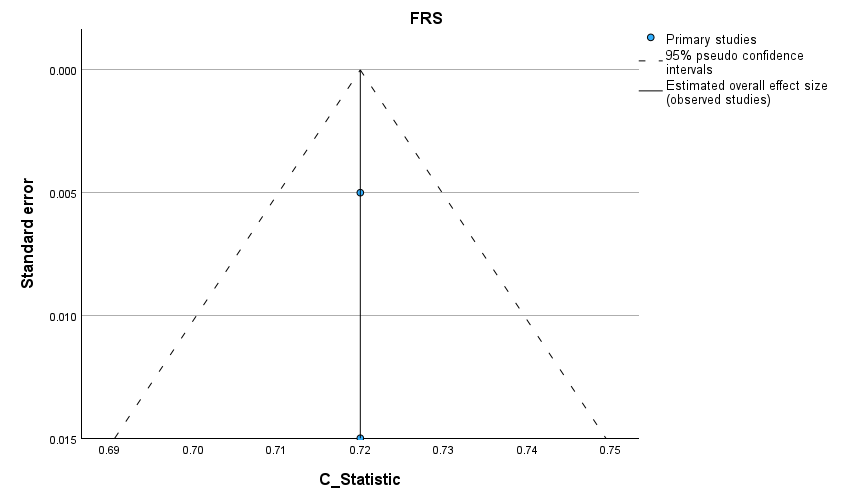


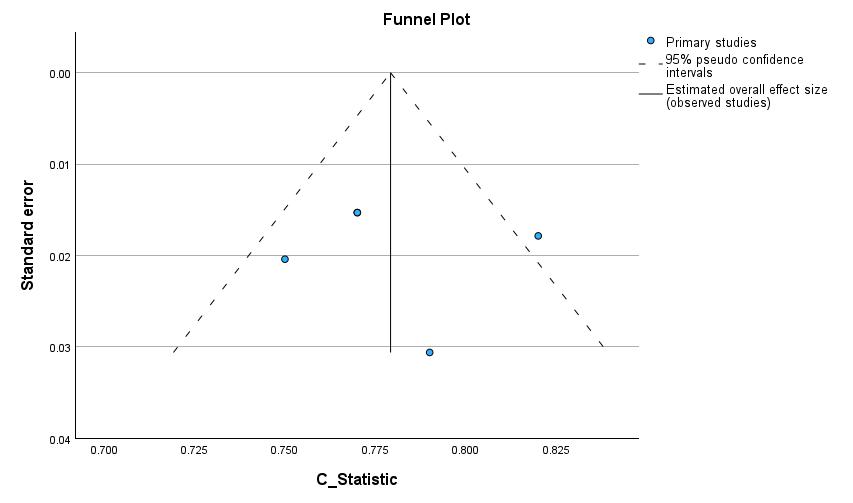


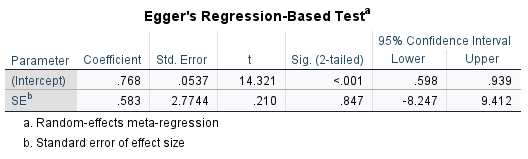


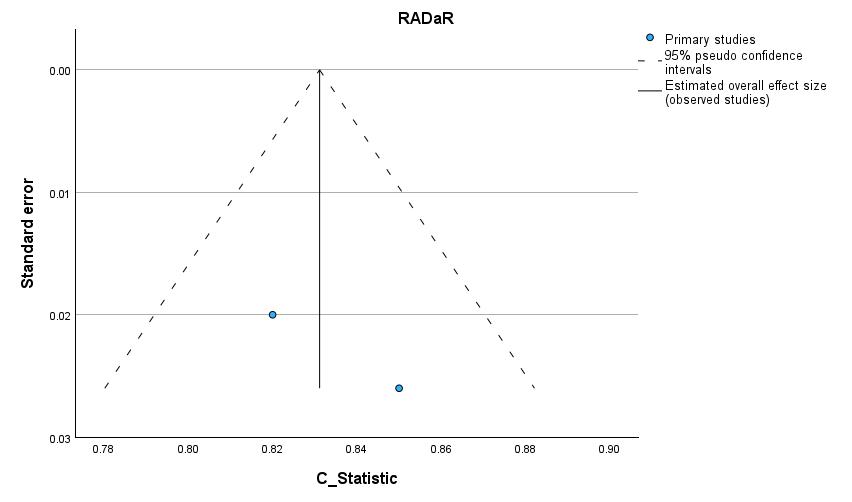


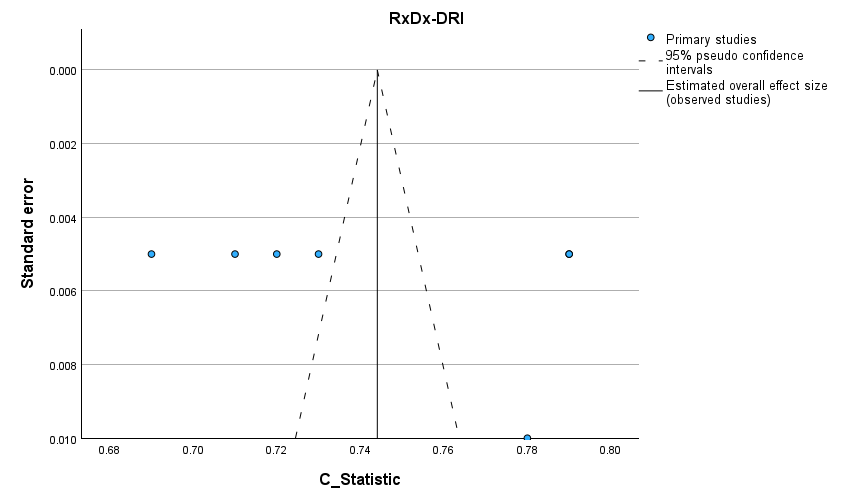


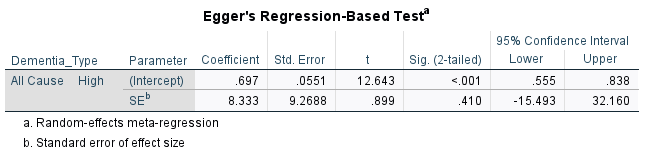


# Table S5 Comparison of the development and external validation study characteristics and methods for variable mapping

|  | No of Validations | Development Sample | Validation Sample 1 | Validation Sample 2 | Validation Sample 3 | Validation Sample 4 | Validation Sample 5 | Validation Sample 6 | Validation Sample 7 | Validation Sample 8 | Validation Sample 9 | Validation Sample 10 | Validation Sample 11 | Validation Sample 12 |
| --- | --- | --- | --- | --- | --- | --- | --- | --- | --- | --- | --- | --- | --- | --- |
| AgeCoDe [58] | 7 | Study: Ageing, Cognition and Dementia Study  N=3,055  Age range: ≥75  Follow-up = mean ​3.8 years (max 6.1 years)  Outcome = AD  Country = Germany​ | Reference: Stephan (2020)  Study: 10/66 Study  N= 637  Age range: ≥65​  Follow-up = 3-5 years​  Outcome = DEM​  Country = China  Substitutions N | Reference: Stephan (2020)  Study: 10/66 Study  N= 1,007  Age range: ≥65​  Follow-up = 3-5 years​  Outcome = DEM​  Country = Cuba  Substitutions N | Reference: Stephan (2020)  Study: 10/66 Study  N= 634  Age range: ≥65​  Follow-up = 3-5 years​  Outcome = DEM​  Country = Dominican Republic  Substitutions N | Reference: Stephan (2020)  Study: 10/66 Study  N= 610  Age range: ≥65​  Follow-up = 3-5 years​  Outcome = DEM​  Country = Dominican Republic  Substitutions N | Reference: Stephan (2020)  Study: 10/66 Study  N= 563  Age range: ≥65​  Follow-up = 3-5 years​  Outcome = DEM​  Country = Peru  Substitutions N | Reference: Stephan (2020)  Study: 10/66 Study  N= 724  Age range: ≥65​  Follow-up = 3-5 years​  Outcome = DEM  Country = Puerto Rico  Substitutions N | Reference: Stephan (2020)  Study: 10/66 Study  N= 394  Age range: ≥65​  Follow-up = 3-5 years​  Outcome = DEM  Country = Venezuela  Substitutions N |  |  |  |  |  |
| Basic-DRM [26] | 9 | Study: Rotterdam Study  N=2,710  Age range: 60-96  Follow-up = median 7 years  Outcome = DEM  Country = Netherlands | Reference: Licher (2019)  Study: EPOZ  N= 514  Age range: 60-90  Follow-up = median 9.5 years  Outcome = DEM  Country = Netherlands  Substitutions N | Reference: Stephan (2020)  Study: 10/66 Study  N= 1,813  Age range: ≥65​  Follow-up = 3-5 years​  Outcome = DEM  Country = China  Substitutions N | Reference: Stephan (2020)  Study: 10/66 Study  N= 2,252  Age range: ≥65​  Follow-up = 3-5 years​  Outcome = DEM  Country = Cuba  Substitutions N | Reference: Stephan (2020)  Study: 10/66 Study  N= 1,433  Age range: ≥65​  Follow-up = 3-5 years​  Outcome = DEM​  Country = Dominican Republic  Substitutions N | Reference: Stephan (2020)  Study: 10/66 Study  N= 1,516  Age range: ≥65  Follow-up = 3-5 years​  Outcome = DEM​  Country = Mexico  Substitutions N | Reference: Stephan (2020)  Study: 10/66 Study  N= 1,307  Age range: ≥65​  Follow-up = 3-5 years​  Outcome = DEM​  Country = Peru  Substitutions N | Reference: Stephan (2020)  Study: 10/66 Study  N= 1,363  Age range: ≥65​  Follow-up = 3-5 years​  Outcome = DEM​  Country = Puerto Rico  Substitutions N | Reference: Stephan (2020)  Study: 10/66 Study  N= 1,331  Age range: ≥65​  Follow-up = 3-5 years​  Outcome = DEM​  Country = Venezuela  Substitutions N | Reference: Vonk (2021)  Study: AGES-RS  N= 5,343  Age range: 66-98  Follow-up = 5-6 & 10  Outcome = DEM​  Country = Iceland  Substitutions N |  |  |  |
| BDSI [59] | 12 | Study: Combined data from: CHS, FHS, HRS, and SALSA  N= ranged 1,125 to 13,889  Age range: ≥65  Follow-up = 6 years  Outcome = DEM  Country = USA | Reference: Capuano (2022)  Study: Combined MPA, ROS & MARS  N= 2,357  Age range: <80  Follow-up = 3 years  Outcome = DEM  Country = USA  Substitutions N | Reference:^1^ Dhana (2024)  Study: CHAP  N= 2,130  Age range: 65-79  Follow-up = 5,6,10,15 years  Outcome = AD  Country = USA  Substitutions N | Reference: Kivimäki (2023)  Study: UKB  N= 465,929  Age range: 38-73  Follow-up = 10 years  Outcome = DEM  Country = UK  Substitutions N | Reference: Licher (2018)  Study: Rotterdam study  N= 6,667  Age range: ≥55  Follow-up = 15 years  Outcome = DEM  Country = Netherlands  Substitutions N | Reference: Stephan (2020)  Study: 10/66 Study  N= 1,564  Age range: ≥65​  Follow-up = 3-5 years​  Outcome = DEM  Country = China  Substitutions N | Reference: Stephan (2020)  Study: 10/66 Study  N= 1,751  Age range: ≥65​  Follow-up = 3-5 years​  Outcome = DEM  Country = Cuba  Substitutions N | Reference: Stephan (2020)  Study: 10/66 Study  N= 1,074  Age range: ≥65​  Follow-up = 3-5 years​  Outcome = DEM​  Country = Dominican Republic  Substitutions N | Reference: Stephan (2020)  Study: 10/66 Study  N= 1,197  Age range: ≥65  Follow-up = 3-5 years​  Outcome = DEM​  Country = Mexico  Substitutions N | Reference: Stephan (2020)  Study: 10/66 Study  N= 994  Age range: ≥65​  Follow-up = 3-5 years​  Outcome = DEM​  Country = Peru  Substitutions N | Reference: Stephan (2020)  Study: 10/66 Study  N= 866  Age range: ≥65​  Follow-up = 3-5 years​  Outcome = DEM​  Country = Puerto Rico  Substitutions N | Reference: Stephan (2020)  Study: 10/66 Study  N= 914  Age range: ≥65​  Follow-up = 3-5 years​  Outcome = DEM​  Country = Venezuela  Substitutions N | Reference: Vonk (2021)  Study: AGES-RS  N= 5,343  Age range: 66-98  Follow-up = 5-6 & 10  Outcome = DEM​  Country = Iceland  Substitutions N |
| CAIDE [57] | 12 | Study: Cardiovascular Risk Factors,  Aging, and Dementia Study  N= 1,409  Age range: 39-64  Follow-up = mean 20 years  Outcome = DMS-IV  Country = Finland | Reference: Dhana (2024)  Study: CHAP  N= 2,130  Age range: ≥65  Follow-up = 5,10,15,20 years  Outcome = AD  Country = USA  Substitutions N | Reference: Fayosse (2020)  Study: Whitehall II  N= 7,553  Age range: 39-63 years  Follow-up = mean 23.5 years  Outcome = DEM  Country = UK  Substitutions N | Reference: Kivimäki (2023)  Study: UKB  N= 465,929  Age range: 38-73  Follow-up = 10 years  Outcome = DEM  Country = UK  Substitutions N | Reference: Licher (2018)  Study: Rotterdam Study  N= 6,667  Age range: ≥55  Follow-up = 15 years  Outcome = DEM  Country = Netherlands  Substitutions N | Reference: Trares (2024)  Study: ESTHER Study  N= 5,360  Age range: 50-75  Follow-up = 17 mean years  Outcome = DEM  Country = Germany  Substitutions N | Reference: Shang (2022)  Study: UKB  N= 471,485  Age range: 38-73  Follow-up = 11.9 median years  Outcome = DEM  Country = Germany  Substitutions N | Reference: Stephan (2020)  Study: 10/66 Study  N= 1,798  Age range: ≥65​  Follow-up = 3-5 years​  Outcome = DEM  Country = Cuba  Substitutions N | Reference: Stephan (2020)  Study: 10/66 Study  N= 1,064  Age range: ≥65​  Follow-up = 3-5 years​  Outcome = DEM​  Country = Dominican Republic  Substitutions N | Reference: Stephan (2020)  Study: 10/66 Study  N= 1,281  Age range: ≥65  Follow-up = 3-5 years​  Outcome = DEM​  Country = Mexico  Substitutions N | Reference: Stephan (2020)  Study: 10/66 Study  N= 542  Age range: ≥65​  Follow-up = 3-5 years​  Outcome = DEM​  Country = Peru  Substitutions N | Reference: Stephan (2020)  Study: 10/66 Study  N= 1,089  Age range: ≥65​  Follow-up = 3-5 years​  Outcome = DEM​  Country = Puerto Rico  Substitutions N | Reference: Stephan (2020)  Study: 10/66 Study  N= 763  Age range: ≥65​  Follow-up = 3-5 years​  Outcome = DEM​  Country = Venezuela  Substitutions N |
| CAIDE+APOE [57] | 4 | Study: Cardiovascular Risk Factors,  Aging, and Dementia Study  N= 1,409  Age range: 39-64  Follow-up = mean 20 years  Outcome = DMS-IV  Country = Finland | Reference:^1^  Dhana (2024)  Study: CHAP  N= 2,130  Age range: ≥65  Follow-up = 5,10,15,20 years  Outcome = AD  Country = USA  Substitutions N | Reference: Kivimäki (2023)  Study: UKB  N= 465,929  Age range: 38-73  Follow-up = 10 years  Outcome = DEM  Country = UK  Substitutions N | Reference: Kivimäki (2023)  Study: UKB  N= 4,865  Age range: 45-69  Follow-up = 20 years  Outcome = DEM  Country = UK  Substitutions N | Reference: Trares (2024)  Study: ESTHER Study  N= 5,360  Age range: 50-75  Follow-up = 14.8 mean years  Outcome = DEM  Country = Germany  Substitutions N |  |  |  |  |  |  |  |  |
| Demographic+APOE [19] | 8 | Study: NA  N= NA  Age range: NA  Follow-up = NA  Outcome = DMS-IV  Country = NA | Reference: Chouraki (2016)  Study: 3 City Study  N= 6,079  Age range: ≥65  Follow-up = 5 - 8 years  Outcome = DEM  Country = France  Substitutions N | Reference: Chouraki (2016)  Study: ACT Study  N= 2,110  Age range: ≥65  Follow-up = 5 - 8 years  Outcome = DEM  Country = USA  Substitutions N | Reference: Chouraki (2016)  Study: AGES-RS  N= 2,553  Age range: ≥65  Follow-up = 5 - 8 years  Outcome = DEM  Country = Iceland  Substitutions N | Reference: Chouraki (2016)  Study: CHS  N= 1,998  Age range: ≥65  Follow-up = 5 - 8 years  Outcome = DEM  Country = USA  Substitutions N | Reference: Chouraki (2016)  Study: FHS  N= 1,757  Age range: ≥65  Follow-up = 5 - 8 years  Outcome = DEM  Country = USA  Substitutions N | Reference: Chouraki (2016)  Study: ROSMAP  N= 1,262  Age range: ≥65  Follow-up = 5 - 8 years  Outcome = DEM  Country = USA  Substitutions N | Reference: Chouraki (2016)  Study: Rotterdam Study  N= 3,334  Age range: ≥65  Follow-up = 5 - 8 years  Outcome = DEM  Country = Netherlands  Substitutions N | Reference: Chouraki (2016)  Study: WHICAP  N= 594  Age range: ≥65  Follow-up = 5 - 8 years  Outcome = DEM  Country = USA  Substitutions N |  |  |  |  |
| DRS-young (60-79) [31] | 5 | Study: THIN  N= 800,013  Age range: 60-79  Follow-up = 5  Outcome = DEM (ICD-10)  Country = UK | Reference: Anatürk (2023)  Study: UKB training dataset  N= 176.611  Age range: ≥50  Follow-up = 14 years  Outcome = DEM  Country = UK  Substitutions N | Reference: Anatürk (2023)  Study: UKB training dataset  N= 2,394  Age range: 57 median age  Follow-up = 14 years  Outcome = DEM  Country = UK  Substitutions N | Reference:  Reeves (2024)  Study: CPRD Gold  N= 419,126  Age range: 60-79  Follow-up = 5 years  Outcome = DEM  Country = UK  Substitutions Y | Reference: Licher (2018)  Study: Rotterdam Study  N= 5,019  Age range: 60-79  Follow-up = 15 years  Outcome = DEM  Country = Netherlands  Substitutions Y | Reference: Walters (2016)  Study: THIN  N= 226,140  Age range: 60-79  Follow-up = 5 years  Outcome = DEM  Country = UK  Substitutions N |  |  |  |  |  |  |  |
| DRS-old (80-95) [31] | 3 | Study: THIN  N= 130,382  Age range: 80-95  Follow-up = 5  Outcome = DEM (ICD-10)  Country = UK | Reference: Licher (2018)  Study: Rotterdam Study  N= 709  Age range: 80-95  Follow-up = 15 years  Outcome = DEM  Country = Netherlands  Substitutions Y | Reference:  Reeves (2024) Study: CPRD Gold  N= 118,717  Age range: 80-89  Follow-up = 5 years  Outcome = DEM  Country = UK  Substitutions Y | Reference: Walters (2016)  Study: THIN  N= 38,084  Age range: 80-95  Follow-up = 5 years  Outcome = DEM  Country = UK  Substitutions N |  |  |  |  |  |  |  |  |  |
| eRADAR [55] | 2 | Study: Kaiser Permanente Washington  N= 4,330  Age range: ≥65  Follow-up = Biennial  Outcome = DEM  Country = USA | Reference: Coley (2023)  Study: KPWA  N= 129,315  Age range: ≥65 (​M=73.5; SD=7.3)  Follow-up = 1 & 1.5​yrs  Outcome = DEM  Country = USA  Substitutions: N | Reference: Coley (2023)  Study: UCSF  N= 13,444  Age range: ≥65 (M=73.7; SD=7.2)  Follow-up = 1 & 1.5yrs  Outcome = DEM  Country = USA  Substitutions: N |  |  |  |  |  |  |  |  |  |  |
| FRS [49] | 2 | Study: Framingham Study  N= 8,491  Age range: 30-74  Follow-up = 12 years  Outcome = CVD risk  Country = USA | Reference: Fayosse (2020)  Study: Whitehall II  N= 7,553  Age range: 39-63  Follow-up = 23.5 median years  Outcome = DEM  Country = UK  Substitutions: N | Reference: Shang (2020)  Study: UKB  N= 471,485  Age range: 38-73  Follow-up = 11.9 median years  Outcome = DEM  Country = UK  Substitutions: N |  |  |  |  |  |  |  |  |  |  |
| GRS-19 [19] | 5 | Study: Kaiser Permanente Washington  N= 4,330  Age range: ≥65  Follow-up = Biennial  Outcome = DEM  Country = USA | Reference: Chouraki (2016)  Study: 3 City Study  N= 3,079  Age range: ≥65  Follow-up = 5 - 8 years  Outcome = DEM  Country = France  Substitutions N | Reference: Chouraki (2016)  Study: ACT Study  N= 2,110  Age range: ≥65  Follow-up = 5 - 8 years  Outcome = DEM  Country = USA  Substitutions N | Reference: Chouraki (2016)  Study: AGES-RS  N= 2,553  Age range: ≥65  Follow-up = 5 - 8 years  Outcome = DEM  Country = Iceland  Substitutions N | Reference: Chouraki (2016)  Study: CHS  N= 1,998  Age range: ≥65  Follow-up = 5 - 8 years  Outcome = DEM  Country = USA  Substitutions N | Reference: Chouraki (2016)  Study: ROSMAP  N= 1,262  Age range: ≥65  Follow-up = 5 - 8 years  Outcome = DEM  Country = USA  Substitutions N |  |  |  |  |  |  |  |
| Nori-ADRD [65] | 7 | Study: OLDW  N= 215,196  Age range: ≥45  Follow-up = 3-8 years  Outcome = ADRD & MCI combined  Country = USA | Reference: John (2022)  Study: MDCR  N= 10m  Age range: NR  Follow-up = 5 years  Outcome = NR  Country = USA  Substitutions N | Reference: John (2022)  Study: IQGER  N= 30m  Age range: NR  Follow-up = 5 years  Outcome = NR  Country = Germany  Substitutions N | Reference: John (2022)  Study: OPSES  N= 85m  Age range: NR  Follow-up = 5 years  Outcome = NR  Country = USA  Substitutions N | Reference: John (2022)  Study: OPEHR  N= 94m  Age range: NR  Follow-up = 5 years  Outcome = NR  Country = USA  Substitutions N | Reference: John (2022)  Study: CPRD  N= 13m  Age range: NR  Follow-up = 5 years  Outcome = NR  Country = UK  Substitutions N | Reference: John (2022)  Study: IPCI  N= 10m  Age range: NR  Follow-up = 5 years  Outcome = NR  Country = Netherlands  Substitutions N | Reference: John (2022)  Study: IMRD  N= 18m  Age range: NR  Follow-up = 5 years  Outcome = NR  Country = UK  Substitutions N |  |  |  |  |  |
| Plasma phospholipids (10-metabolite panel) [60] | 2 | Study: Rochester/Orange County Aging Study  N= 101  Age range: ≥70  Follow-up = 2-3 years  Outcome = aMCI/AD combined  Country = USA | Reference:^2^ Casanova (2016)  Study: BLSA  N= 195  Age range: mean age 77.2 (SD 6.6)  Follow-up = mean 4.8 years  Outcome = AD  Country = USA  Substitutions N | Reference:^2^  Casanova (2016)  Study: AGES-RS  N= 200  Age range: mean age 78.2 (SD 4.4)  Follow-up = mean 2.6 years  Outcome = AD  Country = Iceland  Substitutions N |  |  |  |  |  |  |  |  |  |  |
| RADaR [17] | 2 | Study: RMAP  N= 1,780  Age range: ≥65  Follow-up = median 9  Outcome = DEM  Country = USA | Reference: Capuano (2022)  Study: ROS  N= 1,299  Age range: ≥65  Follow-up = median 10  Outcome = DEM  Country = USA  Substitutions N | Reference: Capuano (2022)  Study: MARS  N= 379  Age range: ≥65  Follow-up = median 8  Outcome = DEM  Country = USA  Substitutions N |  |  |  |  |  |  |  |  |  |  |
| RxDx-DRI [56] | 7 | Study: CPRD (2003-2012)  N= 133,176  Age range: ≥60  Follow-up = Not clear  Outcome = DEM  Country = UK | Reference: John (2022)  Study: MDCR  N= 10m  Age range: NR  Follow-up = 5 years  Outcome = NR  Country = USA  Substitutions N | Reference: John (2022)  Study: IQGER  N= 30m  Age range: NR  Follow-up = 5 years  Outcome = NR  Country = Germany  Substitutions N | Reference: John (2022)  Study: OPSES  N= 85m  Age range: NR  Follow-up = 5 years  Outcome = NR  Country = USA  Substitutions N | Reference: John (2022)  Study: OPEHR  N= 94m  Age range: NR  Follow-up = 5 years  Outcome = NR  Country = USA  Substitutions N | Reference: John (2022)  Study: CPRD  N= 13m  Age range: NR  Follow-up = 5 years  Outcome = NR  Country = UK  Substitutions N | Reference: John (2022)  Study: IPCI  N= 10m  Age range: NR  Follow-up = 5 years  Outcome = NR  Country = Netherlands  Substitutions N | Reference: John (2022)  Study: IMRD  N= 18m  Age range: NR  Follow-up = 5 years  Outcome = NR  Country = UK  Substitutions N |  |  |  |  |  |

^1^Paper presented data stratified by ethnicity. Two separate analyses were entered into the meta-analysis.

^2^95%CI not reported and not included in meta-analysis

ACT = Adult Changes in Thought, DEM = All-cause dementia, KPWA = Kaiser Permanente Washington

M = Mean, SD = Standard deviation

UCSF = University of California San Francisco Health

Yrs = Years

CVD = cardiovascular disease
